# Critical evaluation of artificial intelligence as a digital twin of pathologists for prostate cancer pathology

**DOI:** 10.1038/s41598-024-55228-w

**Published:** 2024-03-04

**Authors:** Okyaz Eminaga, Mahmoud Abbas, Christian Kunder, Yuri Tolkach, Ryan Han, James D. Brooks, Rosalie Nolley, Axel Semjonow, Martin Boegemann, Robert West, Jin Long, Richard E. Fan, Olaf Bettendorf

**Affiliations:** 1 AI Vobis, Palo Alto, CA 94306 USA; 2 Department of Pathology, Prostate Center, University Hospital Muenster, Muenster, Germany; 3 Department of Pathology, Stanford University School of Medicine, Stanford, USA; 4 Department of Pathology, Cologne University Hospital, Cologne, Germany; 5 Department of Computer Science, Stanford University, Stanford, USA; 6 Department of Urology, Stanford University School of Medicine, Stanford, CA USA; 7 Department of Urology, Prostate Center, University Hospital Muenster, Muenster, Germany; 8 Department of Pediatrics, Stanford University School of Medicine, Stanford, USA; 9 Institute for Pathology and Cytology, Schuettorf, Germany

**Keywords:** Artificial intelligence, Prostate cancer, Gleason grading system, ISUP, Deep learning, Automation, Stress tests, Digital twin, Pathology, Prostate, Computational models, Image processing, Machine learning

## Abstract

Prostate cancer pathology plays a crucial role in clinical management but is time-consuming. Artificial intelligence (AI) shows promise in detecting prostate cancer and grading patterns. We tested an AI-based digital twin of a pathologist, vPatho, on 2603 histological images of prostate tissue stained with hematoxylin and eosin. We analyzed various factors influencing tumor grade discordance between the vPatho system and six human pathologists. Our results demonstrated that vPatho achieved comparable performance in prostate cancer detection and tumor volume estimation, as reported in the literature. The concordance levels between vPatho and human pathologists were examined. Notably, moderate to substantial agreement was observed in identifying complementary histological features such as ductal, cribriform, nerve, blood vessel, and lymphocyte infiltration. However, concordance in tumor grading decreased when applied to prostatectomy specimens (κ = 0.44) compared to biopsy cores (κ = 0.70). Adjusting the decision threshold for the secondary Gleason pattern from 5 to 10% improved the concordance level between pathologists and vPatho for tumor grading on prostatectomy specimens (κ from 0.44 to 0.64). Potential causes of grade discordance included the vertical extent of tumors toward the prostate boundary and the proportions of slides with prostate cancer. Gleason pattern 4 was particularly associated with this population. Notably, the grade according to vPatho was not specific to any of the six pathologists involved in routine clinical grading. In conclusion, our study highlights the potential utility of AI in developing a digital twin for a pathologist. This approach can help uncover limitations in AI adoption and the practical application of the current grading system for prostate cancer pathology.

## Introduction

Prostate cancer (PCa) is the most commonly diagnosed cancer in men and one of the most prevalent causes of cancer-related death^1^. PCa is usually diagnosed via prostate needle biopsy and may be followed by radical prostatectomy (total removal of the prostate, seminal vesicles, and surrounding tissues)^2^. The management of patients who undergo prostatectomy requires a reliable histopathological evaluation, including the determination of tumor extent and other cancer-related metrics (particularly grading, staging, and tumor volume)^3^. However, documenting the spatial distribution of PCa tissue remains a challenging task since manual segmentation of cancer tissue and grading on histological slides are time-consuming, particularly for prostatectomy specimens. Additionally, the pathological characterization of prostatectomy specimens or biopsy cores requires extensive histological sampling (e.g., embedding in multiple blocks) for accurate tumor grading, staging, and volume estimation^4,5^. The automated identification and delineation of PCa histology could drastically improve the speed of clinical workflows and provide accurate and detailed documentation for clinical and research usage.

Recent advances in artificial intelligence (AI), especially in digital pathology, have shown great potential for automated cancer detection and tumor grading from histology images^6–15^. Despite promising results, little is known about how far AI is utilized as a digital twin to accomplish tasks frequently occurring during clinical routine and research and to identify challenges in the current grading system. Therefore, we propose different test conditions to simulate these tasks to identify the utilization boundary of AI as a digital twin for managing PCa pathology.

## Results

**Figure 1:**
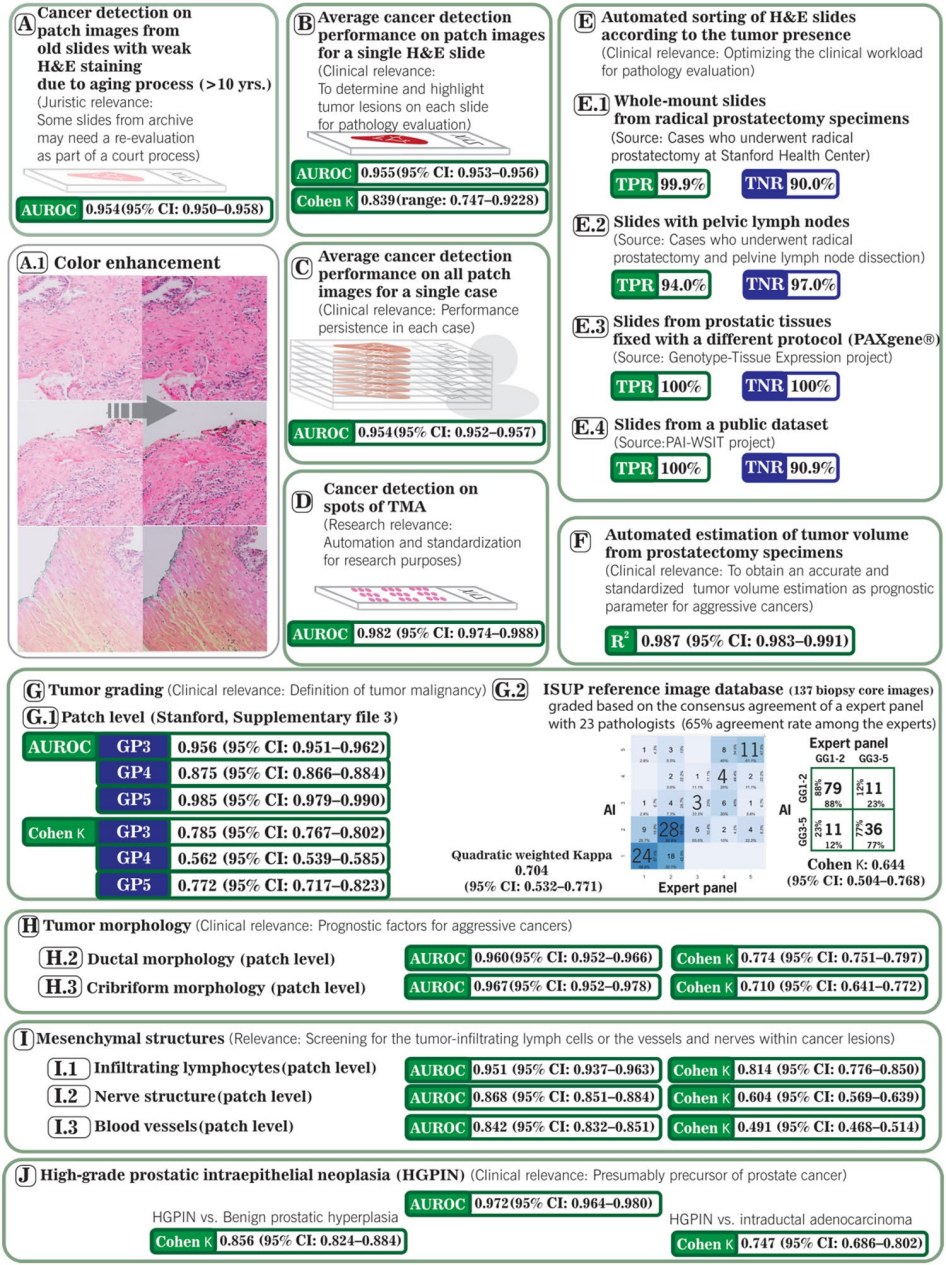
Performance of the digital twin under ten test conditions. (A-I) A.1 illustrates the results of the color optimization using a modified version of Macenko. (B) and (C) Whole-mount H&E-stained sections. (J) All H&E-stained images of prostatectomy specimens from 136 patients were subjected to this test. The relevance of each test is explained. TPR: true positive rate, TNR: true negative rate; AUC: area under the receiver operating characteristic curve; TMA: tissue microarray; H&E: hematoxylin and eosin staining. 95% confidence intervals (CIs) for uncertainty measurements. GTEx: The Genotype-Tissue Expression (GTEx) project (the images originated from tissues processed using alternative fixatives to formalin; only slides showing preserved glandular morphology were considered for the evaluation and included images with prostatitis). The 11th test condition is provided in the supplementary results section. The results are provided in more detail in the supplementary results section.

Figure 1 provides a summary of the evaluation results of the digital twin (vPatho) under ten test conditions. The detailed results and the results for test conditions targeting cancer morphologies (cribriform pattern and ductal morphology) and (mesenchymal tissue structure) and tumor precursors (HGPNs), as well as the results from the vPatho assessment integrated into the electronic pathology reports, are provided in the supplementary results section.

### Prostate cancer

The concordance level for the delineation of prostate cancer (PCa) areas (defined based on tiled patches) between the pathologist’s readings and vPatho was substantial, with a per-slide Cohen kappa score of 0.8385 (range: 0.7468–0.9284) and a per-slide area under the receiver operating characteristic (AUROC) curve of 0.955 (95% confidence interval (CI)-: 0.953–0.956).

The per-slide AUROC was further comparable to the per-case AUROC for PCa detection, emphasizing the consistency of the performance of each radical prostatectomy specimen for patchwise tumor detection when dividing its complete slides into patches.

When we considered whole-slide (WS) images with white H&E-stained sections archived for more than 20 years, we found that the PCa detection performance on 11,862 patches was comparable to the detection performance on the patches from the recent WS (AUROC: 0.95; 95% CI 0.950–0.958). Moreover, this comparable performance was achieved by the computationally improved staining conditions of these WS images.

Another test condition evaluated vPatho for its accuracy in sorting slides according to the presence of PCa in multiple external datasets. We found that this sorting algorithm delivered excellent sorting accuracy on slide images obtained by different studies, indicating the generalizability of vPatho (see Fig. 1E).

When we focused on images of the whole mount slides (WMs) used for pathology evaluation during routine clinical practice and sorted them according to the predicted cancer presence status, 99.0% (1018 of 1028) of the WM histology slides were correctly classified for the presence of PCa; the PPV (positive predictive value) was 99.14%, while the NPV (negative predictive value) was 98.75%. The TPR (true positive rate) was 99.9%, and the TNR (true negative rate) was 90.0% (Fig. 1E.1). Furthermore, a real pathologist reduced the number of normal tissue slides examined by 90.0% using vPatho, while only a single slide was missing from 1028 examined slides from 136 patients.

At the case level, we identified 7 erroneous cases per 100 cases. When false sorting occurred in patients with 8 whole-mount slides on average, at least one slide was falsely sorted. When we examined the causes of these errors, we found that one of the 7 patients had a false negative slide, whereas the remaining 6 patients had at least one false positive slide. The error rate for falsely sorted slides in affected patients was 14% (range: 11–29%), which was significantly greater than the overall error rate for examined slides (1%).

vPatho could detect lymph nodes with PCa metastases with a TPR of 89% and a TNR of 97%, although vPatho was trained neither on images from lymph nodes nor on PCa metastases.

The predicted tumor volumes were strongly correlated with the ground truth, with a coefficient of determination (R^2^) of 0.987 (95% CI 0.983–0.991) in 46 patients (368 WM histology images) whose histology images had complete and detailed annotations of the cancerous lesions (Fig. 1F). The supplementary Results section provides additional results concerning the presence of cancer via vPatho.

### Gleason patterns and ISUP grading

Under the test conditions for the identification of Gleason patterns on images with prostatic tissues in a dimension (~ 512 µm) suitable for laser microdissection, we determined that vPatho provided a substantial concordance level with the pathologist’s annotations for Gleason pattern 3 (Cohen κ: 0.785) and 5 (Cohen κ: 0.772). In contrast, moderate concordance was found for Gleason pattern 4 (Cohen κ: 0.562) between vPatho and the pathologists’ annotations (see Fig. 1G, I). In parallel, we found that Gleason pattern 5 was detected more accurately at 10 × objective magnification, whereas Gleason patterns 3 and 4 were detected better when lesions were generated at 20 × objective magnification, indicating that the magnification of the Gleason pattern was dependent on the quality of the magnification (see the supplementary results section).

The concordance level for the ISUP grade on 137 images from biopsy cores between vPatho and the expert panel with up to 23 pathologists was substantial (quadratic weighted κ: 0.70; 95% CI 0.53 0.77). When we binarized the ISUP grades into GG1-2 and GG3-5, the consensus rate for GG1-2 (78.2%; 95% CI 69.0–85.8%) between vPatho and the expert panel did not significantly differ from that for GG3-5 (62.1%; 95% CI 48.3–74.5) (*P* = 1.000), indicating that our AI algorithms did not significantly affect any of the subgroups that reflect the malignancy grades of PCa (low-grade vs. high-grade).

On 136 prostatectomy specimens, the concordance level between the pathology reports curated by six different genitourinary pathologists during the clinical routine and vPatho for ISUP grading was moderate (quadratic weighted κ = 0.44) before correcting the threshold (5%) for reporting the secondary Gleason pattern. Notably, the primary and secondary Gleason patterns are essential for defining the ISUP grade and represent the most frequent Gleason patterns in radical prostatectomy. The definition of the secondary Gleason pattern depends largely on the arbitrary threshold of 5%. If the second most common Gleason pattern falls below 5%, the primary Gleason pattern is also considered a secondary Gleason pattern, and the second most common Gleason pattern is considered a tertiary Gleason pattern.

**Figure 2:**
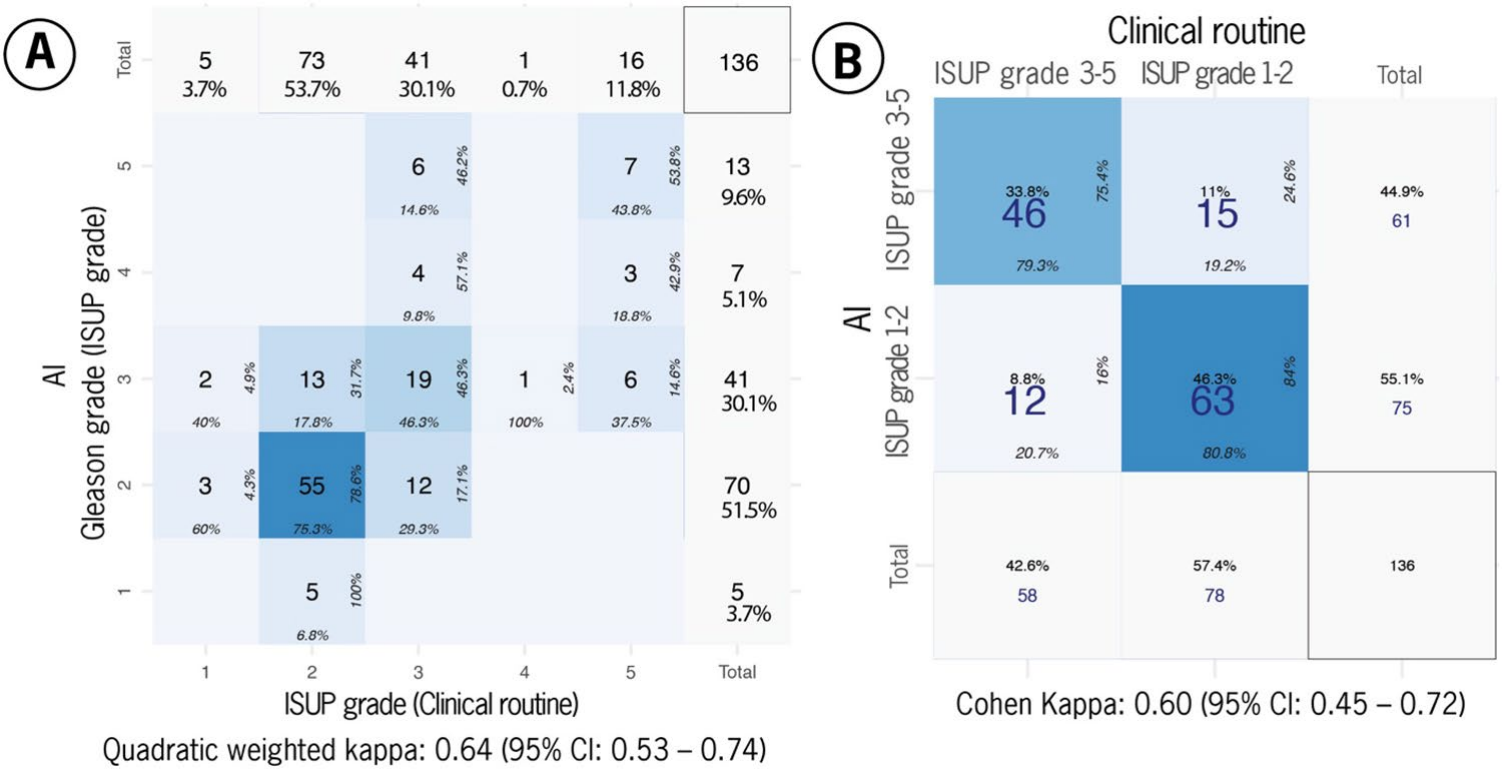
Confusion matrices for (**A**) ISUP grades and (**B**) binary grade groups (ISUP grade groups 1–2 vs. grade groups 3–5) on 136 radical prostatectomy specimens. Kappa values are provided for each endpoint.

Given that our previous study on an independent cohort with radical prostatectomy specimens revealed a pathologist-related underestimation of the tumor area (underestimation bias) by approximately half (50%) of the original tumor percentage^4^, we corrected this threshold to 10%. Correcting the threshold to 10% significantly improved the concordance level between vPatho and pathology reports from moderate to substantial (quadratic weighted κ: 0.64; 95% CI 0.54–0.74). Figure 2A shows the confusion matrix for ISUP grading. Here, we found a total of 81 consensus patients (60%) and 55 nonconsensus patients (40%).

When we divided the ISUP grades into GG1-2 and GG3-5 (Fig. 2B), the consensus rate for GG1-2 (70%; 95% CI 59–79%) between vPatho and pathology reports was comparable to that for GG3-5 (63%; 95% CI 51–74%), indicating no biased preference of vPatho toward any of these subgroups (*P* = 0.700). We found that 80% of the patients (n = 109) received the same binarized ISUP grade according to vPatho and other pathologists, while the remaining 20% of the patients did not (n = 27).

### Factors associated with the ISUP grade

We investigated the associations of histopathological factors with malignancy grade (i.e., mismatch in ISUP grade between the vPatho and pathology reports). This evaluation included the pathologists who performed the ISUP grading on the prostatectomy specimens and factors describing the number of slides per patient, the extent of tumor and tumor grade. The factors for tumor extent were the proportion of slides with PCa and the tumor volume as a percentage (TuVol%). Furthermore, the tumor grades defined by vPatho and pathologists were considered. Since the complementary version of the Gleason grading system was introduced in 2016 and its adaptation in clinical practice is sometimes needed, we also included the year of tumor grading. For each patient, the pathological tumor stage and surgical margin status were also collected and incorporated in our analyses. Finally, we conducted mediation analyses to investigate the interaction effects between the significant indicators and grade disagreement. Detailed statistical results can be found in the supplementary section (see the supplementary results section).

**Figure 3:**
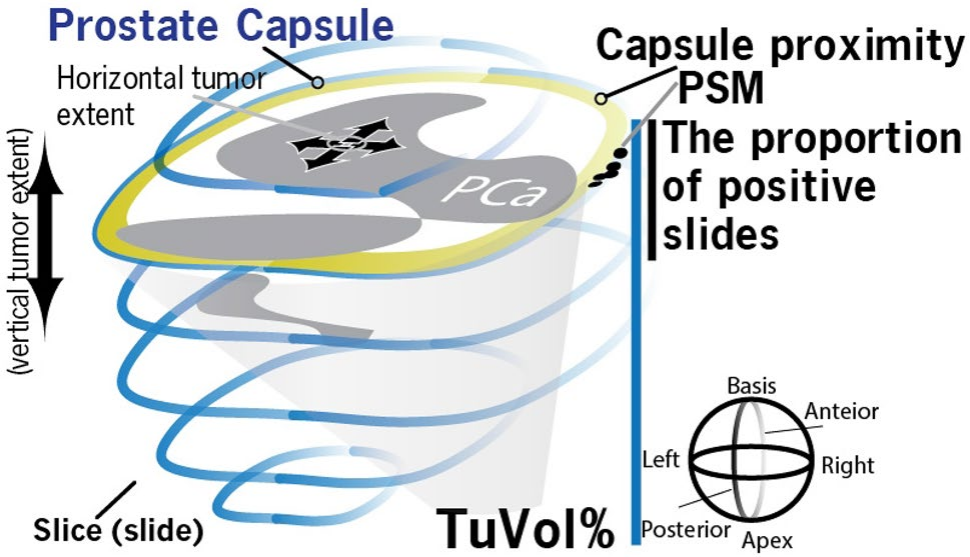
Shows a schematic 3D model of the prostate with the definition of a positive surgical margin (PSM), the proximity of the capsule to prostate cancer, the proportion of positive slides, the percentage of tumor volume (TuVol%) and the definitions of horizontal and vertical tumor extent. Each slice represents a single whole-mount slide. Our analyses with multiple mixed-effects regression models and mediation analyses revealed that the proportion of prostate cancer (PCa)-positive slides and the proximity of the prostate cancer capsule were indicative of the degree to which the tumor grade was concordant between the AI algorithms and pathology reports. While the capsule proximity status was indicative of grade, an increase in the proportion of positive slides was indicative of grade agreement. Because a single radical prostatectomy specimen cannot be embedded into a single block and investigated at once, this specimen was dissected into multiple slices (whole mount). PSM: Positive surgical margins.

Our analyses revealed that the ISUP grade determined by pathologists or by our AI algorithms was associated with grade disagreement when the tumor stage (pT), locoregional lymph node metastasis status (pN) and surgical margin (R) status were not considered for adjustment of the multivariable models. However, when the multivariable mixed-effects regression model included pT, pN and R status, the ISUP grade was no longer a significant indicator of grade discordance. Interestingly, the capsule proximity and the proportion of positive slides were indicative of grade disagreement, suggesting that other confounding factors defining the tumor extent direction (i.e., horizontal tumor extent3 toward the prostate boundary and the number of positive slides for vertical tumor extension) contributed to the grade concordance status between the AI algorithms and pathology reports (Fig. 3 and supplementary results section). We could not identify individual pathologists as a significant indicator of the grade discordance between the AI algorithm and the pathology report curated during routine clinical practice. The year of grading was weakly associated with the pathologists but not indicative of grade disagreement, emphasizing the lack of association between grade and the adaptation period for the recent ISUP grading system in clinical practice.

Although TuVol% was associated with the proportion of positive slides and was significantly twofold greater in patients with positive capsule proximity than in patients with negative capsule proximity, TuVol% was not indicative of the grade concordance between the AI algorithms and pathology reports, implying that the tumor expansion range (TuVol%) was not enough to trigger grade disagreement.

**Figure 4:**
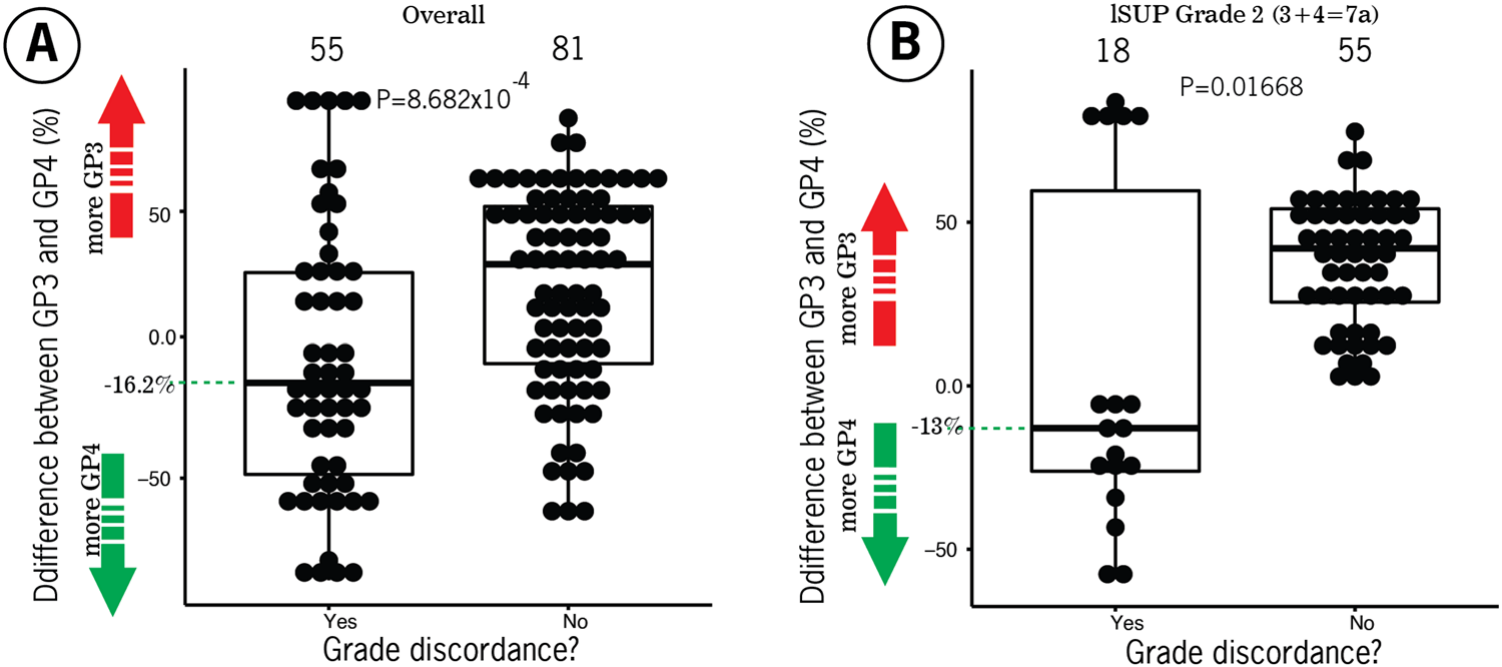
Box plots showing the differences in tumor incidence between patients with Gleason pattern 3 and 4 tumors stratified by tumor grade (**A**) and between patients with an ISUP grade 2 and patients in the pathology report (**B**).

**Figure 5:**
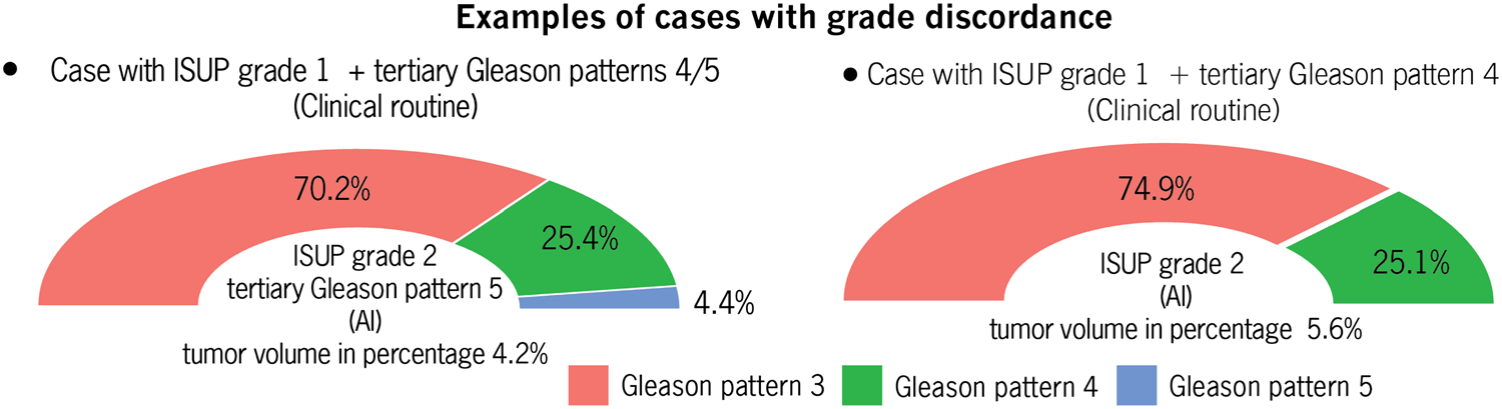
Two example cases with grades between vPatho and the pathology reports. The proportions were estimated by vPatho (AI), and the resulting ISUP grade and tertiary Gleason pattern were compared with pathology reports (clinical routine). The tumor volume as a percentage was calculated by vPatho. Although both the vPatho and pathology results revealed the right Gleason pattern in these patients, differences in the ISUP grade were observed. Although the Gleason pattern 4 detected by vPatho was affected by a greater false negative rate than was the other Gleason patterns 3 and 5, we found that the grade difference between vPatho and pathology reports was associated with a greater proportion of estimations by vPatho for Gleason pattern 4 than by that for Gleason pattern 3 (see Fig. 4). This finding revealed that the proportion of patients with a Gleason pattern marked as a tertiary Gleason pattern by pathologists is twice as high as the 10% threshold (a corrected threshold for determining the secondary Gleason pattern), indirectly highlighting the inaccurate size estimation (50% of the original size) made by human observers (pathologists). This result is in accordance with our previous study in which we showed that human observers (pathologists) significantly underestimate the size proportion (by 50% of the original tumor) compared to computer-assisted size estimation on an independent dataset with 255 prostatectomy specimens^4^.

Finally, we found that the absolute overall deviation between Gleason pattern 3 (GP3) and 4 (GP4) from the zero point (a point where the percentages of GP3 and GP4 are equal) was similar between patients with grade (34.1%) and those with grade concordance (35.6%), emphasizing that the absolute deviation from the zero point is not relevant for grade (*P* = 0.1521). In contrast, the median percentage difference between these Gleason patterns was − 16.2% (interquartile range, IQR: − 34.2– − 4.9%) (negative sign: more GP4) for patients with grade discordance and 25.8% (IQR -23.6–43.7%) (positive sign: more GP3) for patients with grade agreement, revealing that the percentage of patients with grade concordance was significantly greater overall (*P* = 0.0005) or in ISUP grade 2 (3 + 4) (*P* = 0.01668), as shown in Fig. 4. Importantly, the median difference between the GP3 and GP4 percentages deviated marginally (by 6.2%) from the 10% threshold for patients with grade disagreement, suggesting the existence of a gray zone (uncertainty range) for the definition of grade concordance between vPatho and pathology reports (or a pool of six clinical pathologists) in a subset of these patients. Figure 5 provides two example cases to stress the impact of the proportion determination in causing the grade discordance between vPatho and the human observer.

## Discussion

The current study evaluated the ability of the digital twin to accomplish tasks recurrently occurring in the clinical routine to evaluate histology slides with prostatic tissues for PCa. Our findings support the conclusion that building an artificial intelligence (AI) solution that acts as a digital twin is promising. Moreover, previous studies have shown a potential improvement in the concordance level between pathologists using AI^15,16^. However, major challenges remain in realizing such AI solutions in the clinical stage and are discussed here.

First, the current study reveals the need to develop guidelines for PCa pathology justified by the limitations and the utilization boundaries of AI algorithms. In addition to image quality assurance^17^, these guidelines incorporate test conditions to ensure the persistent efficacy of AI algorithms on different amounts of tissue ranging from microdissected tissues to radical prostatectomy specimens; additionally, they suggest strategies to overcome AI limitations and the feasibility of integrating the evaluation results from AI algorithms into an electronic pathology report.

Since the definition of an expert panel may occur in a closed circle and is subject to several biases (e.g., social network bias, implicit bias, situational bias and geographic bias), we also compared our conclusions with the conclusions of other geographically distinct studies, as listed in Table 1. Studies by Al Nemer et al.^18^ and Dere et al.^19^ assessed biopsy slides, revealing different levels of concordance among Western Asian pathologists on the ISUP grade group system: Al Nemer found substantial agreement, while Dere reported a moderate level. Egevad et al.^20^ observed a high level of consensus on the ISUP grade group among pathologists from Europe, North and South America, Eastern Asia, Australia, and New Zealand. In Southern Europe, Giunchi et al.^21^ examined various prostate samples, noting a substantial consensus in grading Prostate Cancer and identifying High-Grade Prostatic Intraepithelial Neoplasia (HGPIN). Van der Slot et al. identified substantial concordance among Western European pathologists on the ISUP grade group for prostatectomy specimens^22^. However, their focus on Gleason patterns 4, 5, and the Cribriform pattern showed a moderate agreement level, indicating a decrease in concordance for more complex pattern recognition compared to ISUP grading only^22^. The concordance levels for vPatho align with those reported in various studies for biopsies and radical prostatectomy specimens (Table 1), supporting the generalizability of our results. Furthermore, our findings of concordance for Gleason pattern 4 between vPatho and pathologists are corroborated by the work of Van der Slot et al.^22^.

**Table 1:** Lists the studies examining the recent version of the ISUP grade groups and other findings for their concordance level and comparison with our concordance levels.

Study description					Concordance level	
Publication	Geographic locations of pathologists	Metrics	Sample description (Geographic origin)	Finding	Study’s conclusion	Our conclusion
Al Nemer et al.^18^	Western Asia	Fleiss kappa	126 slides with biopsy cores (Western Asia)	ISUP grade group	Substantial	Substantial
Dere et al.^19^	Western Asia	Fleiss kappa	50 biopsy slides from 41 cases (Western Asia)	ISUP grade group	Moderate	Substantial
Egevad et al.^20^	Western Europe, North Europe, North America, South America, Eastern Asia, Australia, and New Zealand	Average weighted kappa	90 core needle biopsies (STHLM3^23^, North Europe)	ISUP grade group	Substantial	Substantial
Giunchi et al.^21^	Southern Europe	Cohen kappa	121 regions of interest from 61 slides covering biopsy, radical prostatectomy, and TUR (Southern Europe)	Prostate Cancer	Substantial	Substantial
				HGPIN	Substantial*	Substantial
van der Slot et al.^22^	Western Europe	Krippendorff’s α	80 radical prostatectomy specimens (Western Europe)	Cribriform pattern	Moderate	Substantial
				ISUP grade group	Substantial	Substantial
				Gleason pattern 4	Moderate	Moderate
				Gleason pattern 5	Moderate	Substantial

The geographic location is defined according to the United Nations geographic scheme. Most geographic regions are still underrepresented in terms of interobserver reproducibility in the recent version of the ISUP/2016 WHO grading system (e.g., Africa and central Asia), highlighting the significant regional disparity in evaluating tumor grade. *The concordance level among genitourinary pathologists is higher than that among general pathologists. HGPIN stands for high-grade prostatic intraepithelial neoplasia. The conclusions of the current study (7/9) are mostly in agreement with the conclusions of these studies investigating the concordance conditions between different pathologists.

Furthermore, using vPatho, we discovered that addressable challenges in the current grading system contributed to the grade in our radical prostatectomy samples, which are as follows:

1. The arbitrary decision threshold for the secondary Gleason pattern. The concordance level for radical prostatectomy between vPatho and pathologists (pathology report) was inferior to that for biopsy cores when the decision threshold for the secondary Gleason pattern was not adjusted for underestimation bias. When the threshold was corrected from 5 to 10% based on the findings of a previous study^4^, the concordance level for the ISUP grade groups significantly improved for radical prostatectomy and consequently eliminated the differences in the concordance levels of the ISUP grade groups between biopsy cores and radical prostatectomy specimens. These conclusive findings emphasize the importance of adjusting the decision thresholds for a better consensus level between artificial intelligence and a pool of pathologists or even between pathologists. While implementing a dynamic threshold could offer a more nuanced approach, it presents technical challenges. Such a threshold would need to be linked to a specific metric to be justifiable; this metric can be the tumor volume or the prostate volume in our case. However, future work is needed to develop and investigate such dynamic thresholding algorithms for tumor grading.
2. The distinction of pathologists, possibly by attention or cognitive bias, is not directly related to the ISUP grade (e.g., topographical tumor spread) due to the limited human capacity for perception^24^. In contrast, the distraction of AI is related to image quality and content (e.g., brightness and the epithelium of the seminal vesicle). Considering distracting factors during model training or image preprocessing can mitigate the effects of distraction while improving the accuracy.
3. The proportion of slides with PCa in a single patient can impact the grade, possibly due to cognitive bias by human observers^25^. vPatho mitigates cognitive bias by considering all slides tiled into small patches labeled with PCa for ISUP grading. Moreover, proportion estimation via computer-assisted planimetry is more accurate and objective than is human observer assessment^4^.
4. The gray zone for the ISUP grade groups, due to the degree of closeness, can lead to this. Since the computer-assisted planimetry of vPatho yields more accurate volume estimations than does the human observer, vPatho intuitively outperforms the human observer in managing borderline cases.

The existence of multiple ISUP grade groups is associated with an increasing likelihood of bias due to increasing grade complexity. In a simulation with an equal probability for each group, reducing the group number from 5 to 2 improved the concordance likelihood between two pathologists from 4 to 25% just by chance. Moreover, the current study revealed that the major driver of discordance was Gleason pattern 4, which was also found in two previous studies^4,13^. A possible strategy to increase the concordance level would be weighing the proportion of Gleason pattern 3 in the group definition (e.g., dominant vs nondominant/rare or absent Gleason pattern 3) instead of Gleason pattern 4, which covers more heterogeneous and diverse patterns with a complex and challenging boundary definition.

By analyzing the limitations of the grading system, we determined that these limitations are rooted in the practical recommendations of the current ISUP grading system, which heavily depend on the subjective estimation of Gleason patterns for ISUP grading^26^. Moreover, the current grading system is adapted to pathologists’ limitations by providing practical measurements for clinical implementation that are not time- or effort-intensive^27^; however, these measurements increase the likelihood of bias^28^. Measurements adjusted to the pathologists’ limitations are not always transferrable to AI given their distinctive nature. Therefore, developing a widely acceptable AI solution requires a specific version of the grading system or prostate cancer reporting system for AI.

Our study reveals the necessity and feasibility of developing standardized test conditions reflecting routine clinical practice. An example of this need is that choosing only one slide per case does not suffice to assert a clinical-grade evaluation for PCa detection, and we should consider more slides per case to accurately measure the true accuracy at the case level. This is because false negatives and positives were found randomly in each case, and not specific to certain cases. Falsely sorting a single slide leads to an error rate between 11 and 29% in a single case depending on the number of slides per case (see Table S25 in the addendum table of the supplementary results section). Therefore, it is clinically more important to report how many patients have falsely sorted slides per 100 patients examined. Furthermore, we emphasize that human examiners require additional efforts to screen for false negative slides (the human examiner needs to screen the whole slide for prostate cancer to confirm or exclude the existence of a false negative slide) than for false positive slides (we require only examining the regions demarcated by vPatho to determine false positive slides). We emphasize that having a negligible false negative rate is useful for directing the focus of pathologists to correct false positive slides with cancer areas demarcated by vPatho.

It is worth mentioning that the detection performance on patches (the deepest level of evaluation) from tissue microarray (ROCAUC: 0.982; 95% Confidence interval -CI- 0.974–0.988) was significantly higher than on patches from prostatectomy specimens (AUROC: 0.955, 95% CI 0.953–0.956) given that the histologic heterogeneity likelihood is higher in prostatectomy specimens and therefore more challenging compared to smaller tissues (i.e., laser microdissection, TMA spots); the curation of tissue microarrays (TMA) may rely on the random area selection, but it still follows a targeted tissue sampling that reduces the histologic heterogeneity spectrum. Our results also indicate that the detection performance for prostate cancer on TMA is not representative of the detection performance on slides of radical prostatectomy specimens at the patch level. Overall, the tissue dimension and tissue heterogeneity of prostate tissue samples impact detection performance. A variety of tissue dimensions should therefore be considered during prostate cancer detection performance evaluation to determine the generalizability of the clinical grade. Additionally, we highlight the importance of reporting the image preprocessing steps, as the definition of image preprocessing is strongly associated with the detection performance under test conditions and is therefore important for deep learning models.

Finally, we further emphasize the importance of designing the test conditions of datasets as completely disjointed from the development set^29^. The study included slides examined during routine clinical procedures to generate pathology reports. Our test design is inspired by previous studies investigating the concordance between pathologists for different pathological findings.

In our study, while the model was trained once and tested across various scenarios, we recognize the critical importance of ongoing monitoring post-deployment, particularly in compliance with FDA and other regulatory approvals. This includes a well-documented review and monitoring process for the AI framework, ensuring it adheres to regulatory standards and maintains the highest level of integrity and safety in clinical applications. Moreover, conducting simulation tests that replicate typical scenarios encountered in clinical practice provides an effective method for evaluating machine learning performance in real-world settings.

The question of which pathologist’s grading is the most accurate remains debatable, and the true prognostic significance of varying gradings on the same patient is still unclear and whether other clinical parameters like tumor stage and PSA levels mitigate the effect of the varying gradings, with only partial insights gained from existing studies. Additionally, exploring the prognostic impact of discordance among pathologists is challenging due to inherent biases. These include variables such as the individual’s perceptual ability, daily workload, prior knowledge, and personal experience, which cannot be easily quantified in a time-dependent manner. For example, a pathologist with five years of experience in a high-volume, non-academic institution, dealing with a variety of PCa cases from different hospitals, may encounter a higher number of heterogeneous PCa cases annually than a pathologist who has ten years of experience working exclusively in a university hospital. Notably, university hospitals take on a more significant role in academic research and are more involved in the development of the grading system, often through collaborative consortium mechanisms with pathologists from different universities and the majority consensus mechanism. In clinical practice, grading is, however, not performed under a consensus mechanism, which means that the consensus reached in a collaborative consortium may not accurately reflect the decision-making process in a clinical setting. Similarly, when comparing AI with pathologists, a more controlled test environment is essential to ensure that bias factors, including selection biases, are minimized.

This study has several limitations. First, this study did not evaluate the correlation between the Gleason grade and survival outcomes given that the follow-up period for an appropriate survival analysis is less than 10 years according to the 2016 WHO grading system^30^. Second, the AI-based digital twin undergoes constant enhancements through iterative improvements in both content and technical aspects. Third, the current study covered only the major spectra of prostate cancer pathology, and the core goal of this study was to evaluate AI under different test conditions reflecting clinical routine and to demonstrate the useability of AI as a digital twin to determine issues associated with the current grading system. Future work will focus on automating tumor staging and pathological descriptions. Finally, all models for GP4 detection were trained using extensive image datasets, including but not limited to Gleason Pattern 4, with the objective of capturing the entire spectrum of GP4 variations as described in the methods section. However, we did not precisely categorize the training set for variations of Gleason Pattern 4. Instead, our emphasis was on creating a high-quality, detailed test set to confirm its coverage of the established GP4 variations. We must also note that while GP4 variation plays a limited role in clinical decision-making, its importance lies in ensuring that the test set encompasses the common patterns of GP4. It would be interesting to explore the significance of underestimating Gleason Pattern 4 (GP4) on the prognosis of outcomes in men who have undergone radical prostatectomy, highlighting the necessity for future studies to determine the extent of this impact.

## Conclusions

Our digital twin concept facilitates trouble-shooting challenges in digital pathology and clinical practice for prostate cancer pathology.

## Methods

### Image database

We utilized publicly available histology images of diagnostic slides provided by The Cancer Genome Atlas (TCGA)^31^ and images of tissue microarrays from a previous study^6^ for model development. For model evaluation under different test conditions (from external datasets), we collected a total of 2540 images of H&E-stained diagnostic histology slides, micrographs, or tissue microarray spots of paraffin-embedded prostate tissues from 709 patients. These slides were obtained by different institutions and scanned using different types of scanners. Table 2 provides a summary of the image datasets for each test condition.

**Table 2:** The description of the cohort utilized to run the test conditions (external validation set).

Test condition	Data description or project source (number of cases), image size	Number of images/patches	Scanner vendor	Objective magnification level
Prostate cancer detection on the H&E slides				
Slide condition or sample dimension				
Old slides (~ 20 years)				
Patch images	McNeal’s anatomy study^32^ (15 cases), ~ 512 × 512 µm	11,862^+^	Philips	10×
Recent slides (< 6 years)				
TMA Spots (Smallest sample dimension)	Stanford TMA database (339 cases), 2048 × 2048 pixels spot images	1129	Leica	20×
All whole mount slides of each single case^+++^ (Largest sample dimension)	Radical prostatectomy (46 cases), whole-mount slide	368	Leica	20×
Tumor volume estimation				
All slides of each single case^+++^	Radical prostatectomy (46 cases), whole-mount slides	368	Leica	20×
Sorting slides according to prostate cancer presence				
Tissue sampling method				
Radical prostatectomy specimens	Radical prostatectomy (136), whole-mount slides	1080	Leica	20×
Dissected pelvic lymph nodes	Lymph node dissection (50 lymph nodes), whole slides	19	Leica	20×
Obduction Cystprostatectomy specimens	The Genotype-Tissue Expression project^33^ (40 cases), whole slides^++^	40	Leica	40×
Radical prostatectomy specimens	PAI-WSIT project^34^ (18 cases), whole slides^++^	60	Hamamatsu	40×
**Gleason pattern detection and ISUP grading**				
Tissue sampling method				
Biopsy cores	The International Society of Urological Pathology image library^35^, 2048 × 2048 pixels (72dpi) micrograph, (137 cases)	137	Olympus (micrograph)	20×
Radical prostatectomy specimens	Radical prostatectomy (136), whole-mount slides	1080	Leica	20×
Very limited tissues with prostate cancer	Patches from 594 random regions with Gleason patterns 3–5 and HGPIN in 24 whole-mount WS images (24 cases), 512 × 512 µm	3840 (20×) 1128 (10×)	Leica	20× 10×
Detection of ductal morphology				
Patch images	Patches from 38 random regions from 2 WS images of 2 cases with ductal adenocarcinoma plus 218 random regions with Gleason pattern 3–5 in 9 WS images of 9 cases, 512 × 512 µm	2112	Leica	10×
Detection of cribriform pattern				
Patch images	Patches from 32 random regions with Cribriform patterns of 5 cases and 199 random regions with noncribriform prostate cancers and Gleason patterns 3–5 in 9 whole-mount WS images (9 cases), ~ 512 × 512 µm	928	Leica	10×
Detection of vessels				
Patch images	Patches from 642 random regions with blood vessels on 22 WS images (22 cases) and 478 random regions with Gleason patterns 3–5 on 20 WS images (20 cases), 512 × 512 µm	4608	Leica	10×
Detection of nerve structure				
Patch images	Patches from 628 random regions with nerves or ganglions on 22 WS images (22 cases) and 216 random regions with Gleason patterns 4–5 (8 slides, 8 cases), 512 × 512 µm	1280	Leica	10×
Detection of inflammatory cell infiltration				
Patch images	Patches from 123 random regions with inflammatory cell infiltration on 19 WS images (19 cases) and 216 random regions with Gleason patterns 4 and 5 (8 slides, 8 cases), ~ 512 × 512 µm	768	Leica	10×
HGPIN detection				
Patch images	Random 32 regions from 10 WM images (10 cases); 40 random regions with intraductal adenocarcinoma from 4 WM images (4 cases); 19 random regions with benign prostatic hyperplasia from 4 WM images (4 cases), ~ 512 × 512 µm	2687	Leica	10×
Integration into an electronic pathology report platform				
Radical prostatectomy specimens	136 radical prostatectomy specimens, complete representative whole-mount slides per case	1028 (Median: 8 per case)	Leica	20×

All histological slides were stained with hematoxylin and eosin. HGPIN: high-grade prostatic intraepithelial neoplasia. + the whole-slide images were tiled into small image patches ++whole slides with a portion of the prostatic slice (2.3 times smaller than the prostatic slice). TMA: tissue microarray. ^+++^Given that a single whole-mount (WM) slice roughly corresponds to 20–30 biopsy cores and because of the time- and labor-intensive effort required for high-precision annotation of WM images for prostate cancer, we randomly selected 46 cases with a total of 368 WM images (~ 7,360–11,040 biopsy core images or ~ 894,240 nonoverlapping tiles (dimensions: 512 × 512 pixels) at 10 × and ~ 1 µm per pixel for patch-level performance evaluation. * The images were obtained at different sites. The negative control groups for the detection of ductal morphology, the cribriform pattern, vessels, nerve structure, and HGPIN are described in the sections below.

### Image processing

Whole-slide images are gigapixel images and are difficult to process in one step. Therefore, we tiled whole-slide images into small patches after identifying the foreground prostatic tissue using image thresholding according to Otsu’s method^36^. We obtained a final patch size of 512 × 512 pixels at 10 × by a pixel mapping of 1 µm per pixel. For Gleason pattern detection, the 20 × objective magnification (~ 256 × 256 µm) was also evaluated to identify the optimal magnification level for the agreement between vPatho and the pathologist.

A patch was considered to be positive for PCa when more than one percent of the patch was positive. A patch was positive for high-grade prostatic intraepithelial neoplasia (HGPIN), Gleason patterns, ductal morphology, the cribriform pattern, nerves, or vessels when at least 5% of the patch was positive.

Spatial annotation data curated by pathologists were used to label these patches. For patchwise evaluation, each histological image has a corresponding mask that incorporates the demarcated lesion areas and has dimensions equal to the dimensions of the original image. Both the image and the mask were tiled using the same grid.

In contrast, the spot images from the TMA were first downsized to achieve a 10 × magnification; then, prostatic tissue was identified by applying image thresholding according to Otsu’s method, and the boundary was determined using the contour detection algorithm provided by the OpenCV framework^37^. After that, the region of interest was divided into tiles of 512 × 512 pixels (512 × 512 µm).

Each TMA spot (each spot was captured at 20 × objective magnification) was previously labeled for PCa presence based on the pathologist’s judgment and whether it originated from cancer lesions in prostatectomy specimens. Accordingly, each patch was labeled based on the spot label.

### Quality assessment

The quality assessment of slides by technical assistants and pathologists is an integral component of the standard operating procedure of accredited pathology institutions^38^. In clinical settings, when a slide is not suitable for pathological evaluation, a better slide is prepared from the same tissue block (prostatectomy specimens are embedded in blocks) for pathological evaluation. We anticipate that similar standards for image quality assessment will become routine in future digital pathology workflows. However, the implementation of a quality management system for histology slides in a clinical setting is beyond the scope of the current study. We consequently refer the reader to the relevant literature.

Although all histology images passed internal review prior to inclusion in the current study, we implemented a blurriness and illuminance assessment tool for patches.

The blurriness of each patch was estimated using the variance of the Laplacian^39^; we first established a reference range (95% confidence interval: 112–124) for blurriness detection on 800 patches randomly generated at 10 × objective magnification from 8 diagnostic H&E WS images available in TCGA-PRAD (100 patches extracted from random regions for each WS image). These slides did not contain visible blurriness or illuminance imbalance. If a patch had a variance outside this range, image sharpening^40^ was applied to reduce the blurriness of the patch. A second blurriness assessment was then made, and if the variance of the patch was still outside the reference range, the patch was excluded from the detection task.

In parallel, we assessed the relative illuminance^41^ of each patch and corrected its illuminance if the relative illuminance was outside the reference range (144.4–165.3). We determined the reference range on 800 patches previously utilized to identify the reference range for blurriness. For illuminance correction, we applied automated contrast limited adaptive histogram equalization (CLAHE)^42^ in combination with a modified version of a reference-free Macenko approach for stain color optimization^43^. Our aim with the illuminance correction was to reduce the illuminance deviation from the reference range. Finally, we evaluated the impact of correcting for blurriness and illumination on the detection performance for spots with prostate cancer using Stanford’s tissue microarray with 1129 spots.

### Model architecture

**Figure 6:**
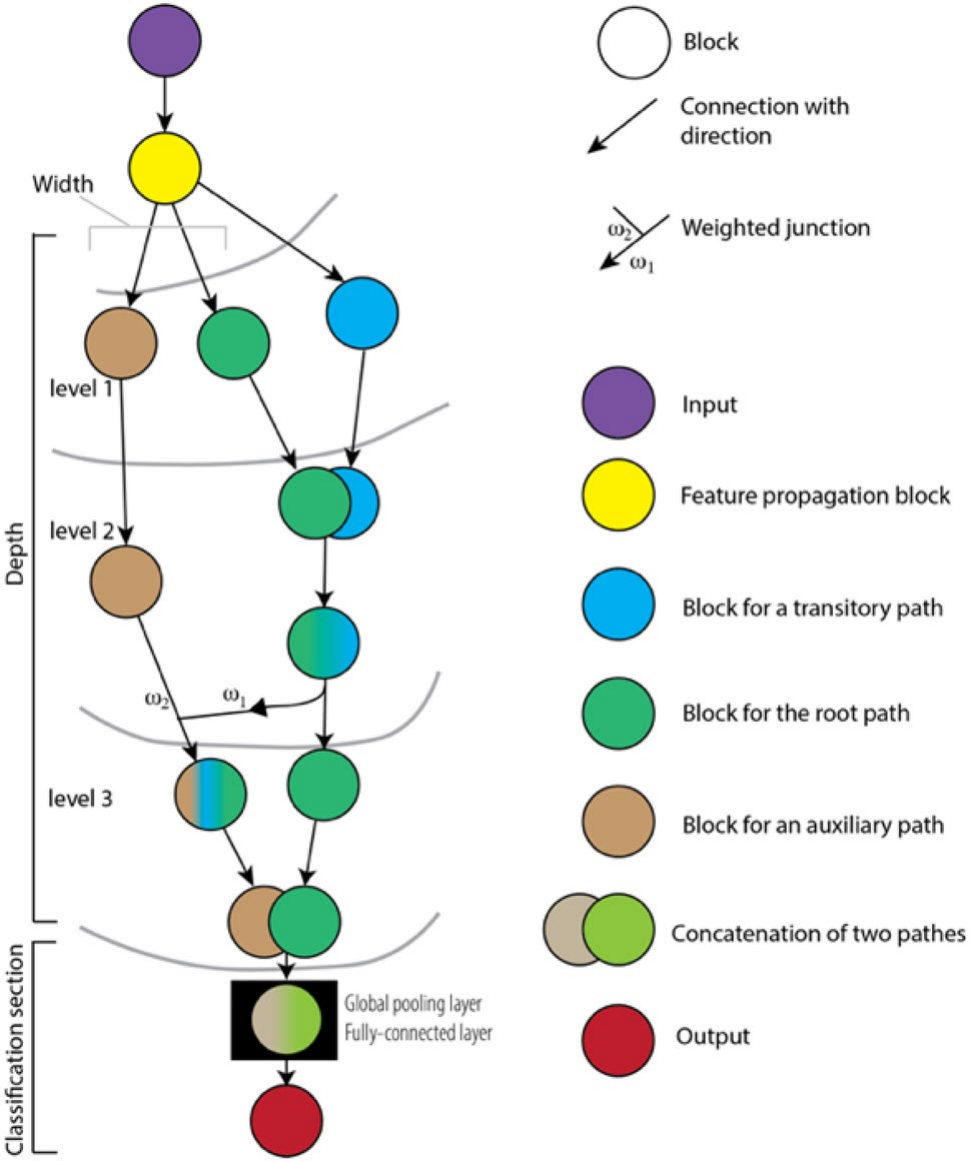
Summarizes the concept of the PlexusNet architecture. A block consists of multiple neural network layers. Four architecture block types are available: VGG, Inception, residual, or soft attention block. The major hyperparameters for the graph definition are the depth (number of levels), with a minimum depth of 2; the number of end-to-end paths (width); the number of transitory “short” paths; and junctions that intersect between two end-to-end paths. Here, the example PlexusNet architecture has a depth of 3 levels, a width of 2, and a single transitory path and weighted junction between two end-to-end paths. The position of the weighted junction between two paths before the global pooling layer is determined randomly. For all the PlexusNet models, all the final feature maps of the end-to-end paths are concatenated before being fed into the global pooling layer. The depth of a transitory path is determined randomly, and the transitory path concatenates with the root path (by default, the first end-to-end path is considered the root path) at the same level as the weighted junction. The position randomization for weighted junctions or the depth for transitory paths has no impact on the model performance, while the number of weighted junctions has an impact on the model performance. For simplified model development, we unified the block type for all paths.

**Figure 7:**
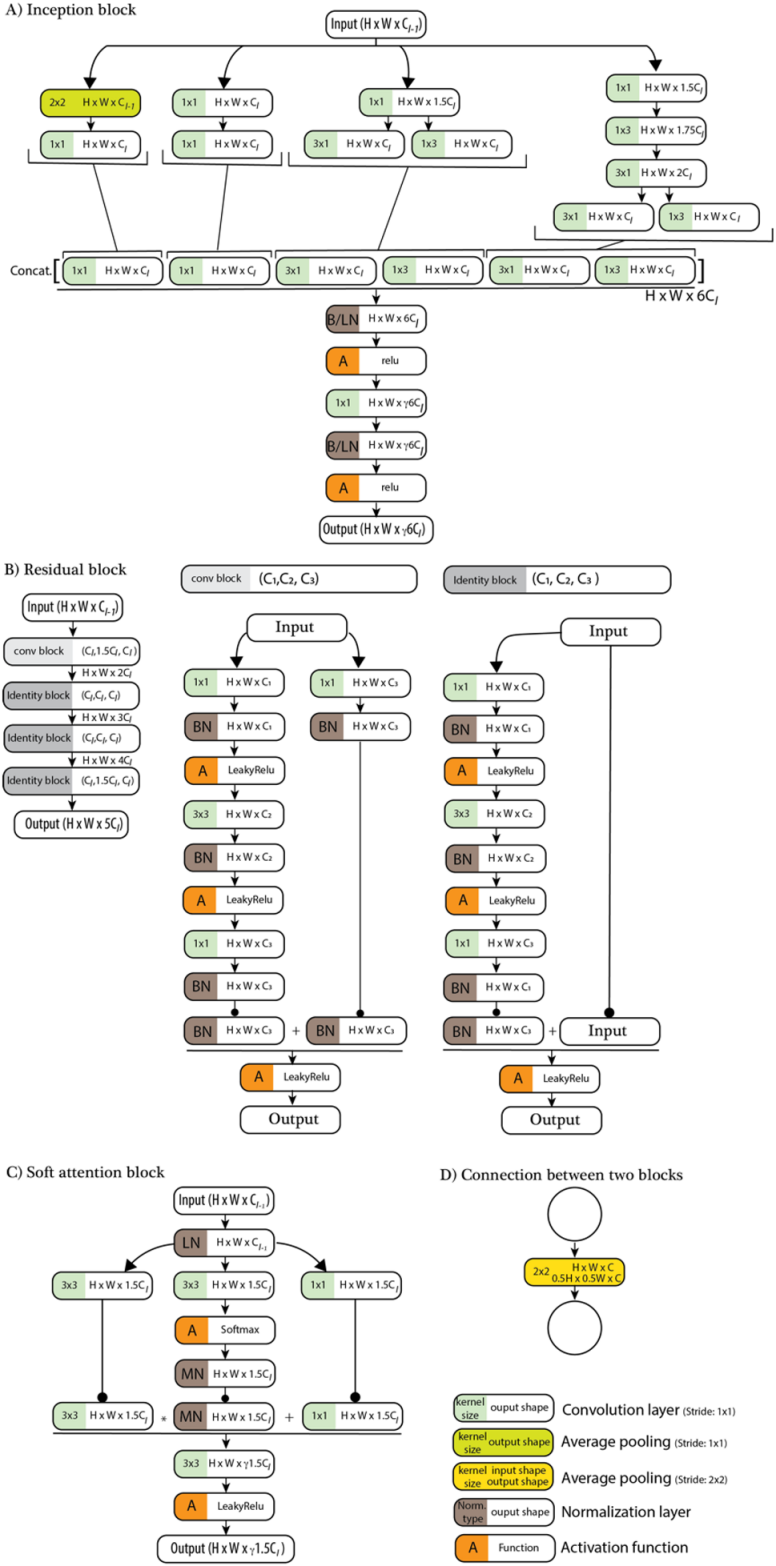
Shows the structures of the (**A**) inception, (**B**) residual, and (**C**) soft attention blocks used in the present study. In the PlexusNet architecture, (**D**) two consecutive blocks are connected by an average pooling that reduces the width and heights of the feature maps of the next block by half. When we calculate the channel numbers, the “round half up” approach is used to convert the channel numbers to integer numbers. BN: batch normalization; MN: max normalization; LN: layer normalization; B/LN: either batch or layer normalization. γ is the compression rate used to reduce the channel information, similar to the compression ratio in DenseNet^49^; l: the level index. H: Height; W: width; C: Channel. The subscript of the level definition for H and W was ignored to emphasize that H and W did not change during tensor processing in each block.

**Figure 8:**
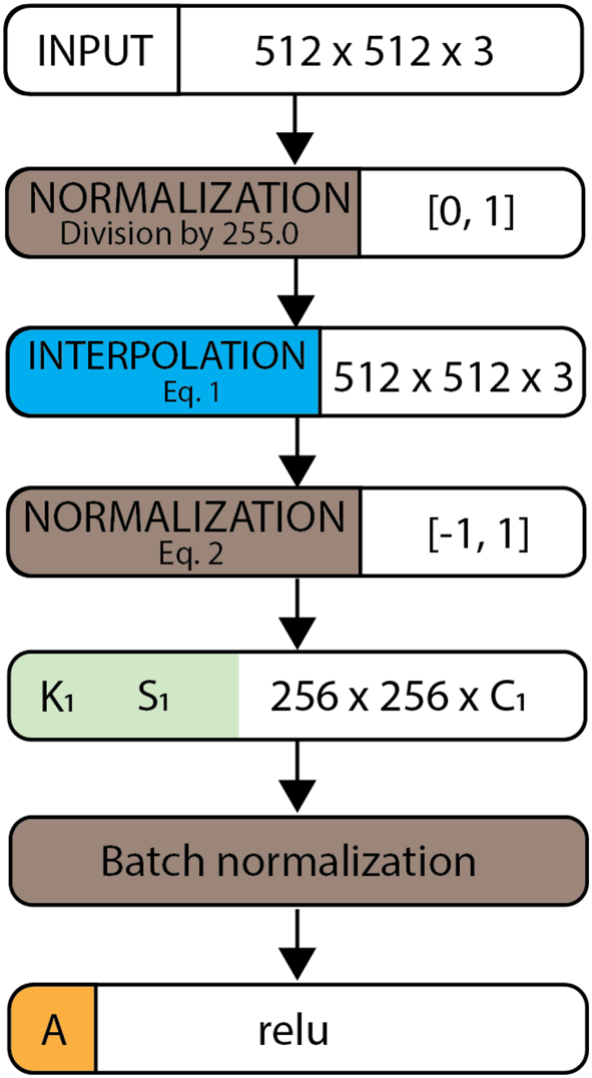
Represents the layers for the feature population block that populate the channel number from 3 to C_1_ using the convolution layer with the size of the convolution weight kernel (K_1_) and a stride (S_1_). The default value for stride S_1_ is 2 × 2 pixels to move K_1_. The dimension unit is given in pixels.

We utilized a novel convolutional neural network called PlexusNet^44^ that supports neural architecture search (NAS)^45^ and uses ResNet^46^ and Inception blocks^47^ as well as standard convolutional blocks (VGGs). Taking inspiration from a previous study, we also included quasisoft attention blocks^48^ (we removed “quasi” from the block description for convenience). The PlexusNet architecture is a directed acyclic multigraph. The complexity of this graph is primarily determined by its depth (i.e., the number of levels), extent of branching (i.e., end-to-end paths or width) and number of weighted junctions (i.e., cross edges) between two end-to-end paths that give rise to the multigraph property. A transitory “short” path was randomly defined to populate feature maps of an end-to-end path at a random level (Fig. 6). The resulting feature maps (i.e., channels) for all the end-to-end paths were concatenated, and their feature dimensions were subsequently reduced via global pooling (either maximum or average pooling). The classification section of PlexusNet fully connects and weights the dense features to feed into the final layer to estimate the confidence scores for pathology in patches of histology images. Figure 6 provides a summary of the PlexusNet architecture concept, and Figs. 7 and 8 illustrate the different block types used in the PlexusNet architecture. Table 3 summarizes the hyperparameter configurations for each finding.

**Table 3:** Summarizes the architecture design for the different findings.

Architecture															
Finding	Block type	Depth	Width	Junction	Short path	C_1_	K_1_	Apply crop center on input (256 × 256)	Initial filter factor per path	Global pooling	No. of Ch. for 1^st^ FC layer	Output function (no. of cat.)	Parameter capacity	Supervised contrastive learning^53^	Transformer^48^ (no. of blocks. header number)
Prostate Cancer	Inception	7	2	3	1	32	5 × 5	No	2	Max	96	Softmax (2)	178,342	No	No
Gleason pattern 3	Inception	4	2	3	1	32	5 × 5	No	2	Max	60	Softmax (2)	58,879	No	No
	Inception	4	2	3	1	32	5 × 5	No	2	Max	60	Softmax (2)	58,879	No	No
Gleason pattern 4	Inception^+^	4	2	3	1	6	5 × 5	No	2	Avg	60	Sigmoid (1)	49,122	Yes	No
	Inception^+^	4	2	3	1	6	5 × 5	No	2	Avg	60	Sigmoid (1)	49,122	Yes	No
	Inception^+^	4	2	3	1	32	5 × 5	No	2	Avg	60	Softmax (2)	50,201	No	No
	Soft attention	6	2	3	1	8	3 × 3	Yes	2	Max	20	Softmax (2)	51,732	No	No
	Soft attention	6	2	3	1	8	3 × 3	Yes	2	Max	20	Softmax (2)	51,732	No	No
ResNet	5	2	3	1	16	5 × 5	No	4	Avg	44	Softmax (2)	206,638	No	No	
Gleason pattern 5	Soft attention	5	2	3	1	16	5 × 5	No	2	Avg	24	Softmax (2)	48,590	No	No
	Soft attention	5	2	3	1	16	5 × 5	No	2	Avg	24	Softmax (2)	48,590	No	No
Ductal morphology	Inception	4	2	3	1	16	5 × 5	No	2	Max	60	Softmax (2)	53,683	No	No
Cribriform pattern	Inception	3	2	3	1	32	5 × 5	No	2	Max	48	Softmax (2)	34,033	No	No
HGPIN	Inception	5	4	2	2	16	5 × 5	No	4	Max	160	Softmax (2)	412,322	No	No
Vessel	Inception^+^	5	2	3	1	16	5 × 5	No	2	Max	72	Softmax (2)	183,448	No	Yes (3,4)
Nerve	Inception^+^	5	2	3	1	16	5 × 5	No	2	Max	72	Softmax (2)	171,997	No	Yes (3,4)
Inflammatory cell infiltration	Inception^+^	5	2	3	1	16	5 × 5	No	2	Max	72	Softmax (2)	171,277	No	Yes (3,4)
**Total**											**1012**		**1,878,587**		

By combining all the models listed here, the novel architecture design achieved a total parameter capacity markedly lower than the parameter capacities of a single ResNet-18 model (~ 11 million) or the 2nd version of a single MobileNet model (~ 2.0 million parameters)^50^. These models combined a total of 1,012 features in the fully connected layers (in comparison, a single RestNet-18 model had 512 features in the fully connected layers). Given the compactness of our models, we could assign all 17 models to a single GPU card (VRAM 24 GB) for the pathology report generation task. For each Gleason pattern, we applied an ensemble model that weighted the predictions of the models equally. The weighted prediction scores ranged between 0 and 1. *Note*: The variation in the parameter capcity despite having the same hyperparameter configuration is due to the variation in the block number (depth) of the short path. A number of models with different configurations were tested to achieve the final model configuration via the grid search and trial-and-error approaches.

*Ch.* Channel, *conv.* Convolution, *C*_*1*_ channel number of the first convolution layer, *K*_*1*_ kernel size of the first convolution layer, *act.* activation, *no.* number, *cat.* Category, *FC* fully connected; +  Layer normalization^51^ is applied instead of batch normalization^52^. Transfomer blocks were added prior to global pooling; since the output of the convolutional layer was batch size × height × width × channel, we reshaped the output to batch size × height ∗ width × channel before feeding into the transformer block; the output from the transformer^48^ was reshaped back to the dimension of the convolutional layer before global pooling was applied. For all the models, a patch dimension of 512 × 512 × 3 is applied.

Significant values are in bold.

We used Eq. (1) to justify the contrast and interpolation of the input images, as this step improved the classification performance for prostate cancer detection by 10.0% (95% CI 9.2–11.2%) compared to that of a convnet model without this interpolation function.

1 $$\widehat{X}=-2{e}^{-{(2X}^{2})}[\mathrm{ cos}\left({90 \omega }_{1}\right)+x \,{\text{sin}}\left({90 \omega }_{2}\right)]$$

$\widehat{X}$ is the output of the equation, where ${\omega }_{1}$ and ${\omega }_{2}$ are the trained weights (scalar), ${{\omega }_{1}, \omega }_{2 } \in [-\mathrm{1,1}]$ and X is a matrix that represents an image batch defined by $\mathrm{X }= \left\{\mathrm{x }\right| \forall x \in X, 0 \le x \le 1\}$; and cos $\left({90 \omega }_{1}\right)$ and sin $\left({90 \omega }_{2}\right)$ terms are the interpolation functions. The optimal weights ${\omega }_{1}$ and ${\omega }_{2}$ are determined during model training.

2 $$\widetilde{X} =2\frac{\widehat{X}-{\text{min}}(\widehat{X})}{{\text{max}}\left(\widehat{X}\right)-{\text{min}}(\widehat{X})}-1$$

**Figure 9:**
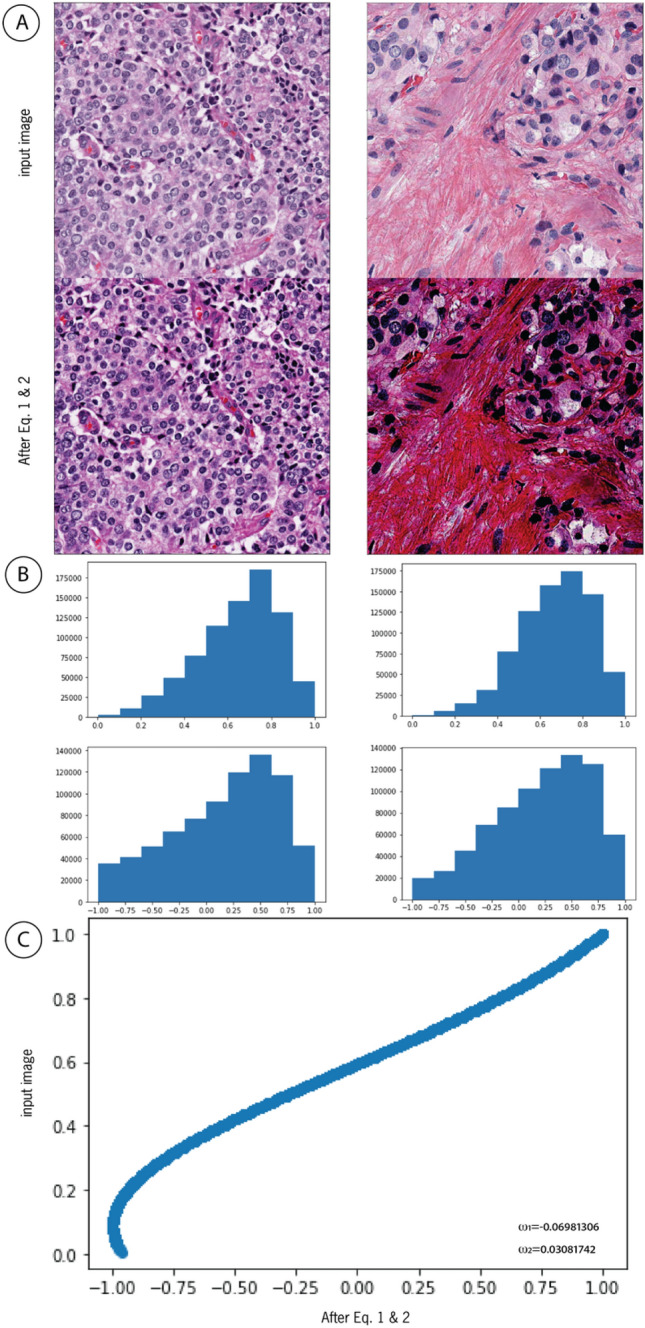
(**A**) shows the same image before and after applying Eqs. (1) and (2) using ${{\omega }_{1}, \omega }_{2}$ provided in (**C**) for comparison. (**B**) Histograms of the same image before and after applying Eqs. (1) and (2). The application of Eq. (1) remarkably improves the image contrast, and (**B**) interpolates the input image according to ${{\omega }_{1}, \omega }_{2}$. PlexusNet-based models learn the optimal values ${{\omega }_{1}, \omega }_{2}$ during model training to determine the optimal nonlinear interpolation of the input image to solve a classification problem. The purpose of this approach is to contrast the semantic content of the input image to increase the likelihood of capturing meaningful features in the deep convolutional neural network layers that consequently impact the classification performance; ${{\omega }_{1}, \omega }_{2}$ given in (**C**) originated from the prostate cancer detection model. The example patch image has dimensions of ~ 512 µm × 512 µm at 10 × objective magnification. (**C**) Values of the input image were normalized using min–max normalization and correlated with the values from Eqs. (1) and (2).

This equation performs [− 1, 1] feature scaling, where $\widetilde{X}$ is the normalized output and $\widehat{X}$ is the batch input that is interpolated by Eq. (1). Figure 9 illustrates the results of Eq. (1) on the input image.

We considered the PlexusNet architecture because this architecture facilitates the development of small models (see parameter capacity in Table 3) for accurate binary classification tasks comparable to those of large state-of-the-art models, as shown in supplementary file 1. It is worth mentioning that the cumulative parameter capacity of the models we considered for vPatho is remarkably below the parameter capacity of a single ResNet 18 model (18 million trainable parameters), a frequently used model architecture. Moreover, the parallel use of these models is feasible on a single GPU card with 24 GB when we have a batch size of 16 patches (dimension: 16 × 512 × 512 × 3).

### Datasets for model development

For training, we followed a data-efficient strategy in which we predefined and determined the proportion of the pathological content of the training set. We followed a trial-and-error approach to determine the optimal proportion of the pathological content of the training set for each detection task. We mitigated the imbalanced classification conditions of the training set by oversampling the underrepresented positive findings and applying image augmentation with a 50% probability to vary the content visualization of the patches. Tables 4, 5, 6, 7, 8, 9 and 10 summarize the data compositions for model development.

**Table 4:** Nonoverlapping patch number and cancer proportion resulting from splitting 200 whole-slide images (WSIs) at 10 × objective magnification by 512 × 512 pixels (1 pixel corresponds to ~ 1 µm) obtained from the Cancer Genome Atlas image library for prostate cancer (PRAD).

Finding	Random data splitting		
	Fold 1	Fold 2	Fold 3
Training set (case number = 195)			
Patches			
Noncancer tissues, n (%)	44,240 (53.99)	43,530 (53.35)	43,975 (54.03)
Prostate cancer, n (%)	37,69 (46.01)	38,069 (46.65)	37,411 (45.97)
Total, n (%)	81,937 (100.00)	81,599 (100.00)	81,386 (100.00)
Average cancer pixel proportion in a patch labeled with prostate cancer (median)	78.8% (100.0%)	78.9% (100.0%)	78.9% (100.0%)
In-training optimization set (case number = 5)			
Patches			
Noncancer tissues, n (%)	1769 (72.0)	2479 (88.7)	2034 (67.6)
Prostate cancer, n (%)	689 (28.0)	317 (11.3)	975 (32.4)
Total, n (%)	2458 (100.0)	2796 (100.0)	3009 (100.0)
Average pixelwise cancer proportion in a patch labeled with prostate cancer (median)	76.7% (100.0%)	64.9% (72.0%)	75.7% (97.0%)

All the slides were scanned at 40 × objective magnification. We randomly selected 200 WSIs for model development, where 195 WSIs were considered for training and 5 WSIs were considered for in-training optimization. These numbers were fixed when curating different folds by random data splitting. All the images were stained with H&E.

**Table 5:** The patch number and the cancer proportions in the external datasets curated from 60 whole-mount histology images.

External dataset for model comparison (60 whole mount images)	
Finding	Patches
Noncancer tissues, n (%)	142,135 (81.95)
Prostate Cancer, n (%)	31,286 (18.05)
Total, n (%)	173,421 (100.00)
Average pixelwise cancer proportion in a patch labeled with prostate cancer (median)	80.6% (100%)

One whole-mount image covers the whole prostatic slice and provides patches on average 6 times more than a single whole-slide image of the Cancer Genome Atlas for prostate cancer.

**Table 6:** The number of patches generated from 641 tissue microarray spot images (2240 × 2240 pixels, scanned at 20 × objective magnification) provided by Arvaniti et al. with spatial annotation for prostate cancer and Gleason patterns prepared by two pathologists^6^.

Finding	Magnification level	Patch number, n (%)
Noncancerous tissues	5×	532 (35.9)
	10×	3325 (35.9)
	20×	4166 (35.9)
Gleason pattern 3	5×	380 (25.7)
	10×	2375 (25.7)
	20×	2932 (25.7)
Gleason pattern 4	5×	328 (22.2)
	10×	2049 (22.2)
	20×	2867 (22.2)
Gleason pattern 5	5×	240 (16.2)
	10×	1500 (16.2)
	20×	1732 (16.2)

The overlap between two patches was predefined to be 50%

**Table 7:** Constitution of the training sets on the basis of Table 4, which aims to cover the variation in the magnification levels for model development.

Finding/models	Considered datasets with magnification levels as training set	Augmentation features (Augmentation probability: 50%)
GP3		Random brightness and contrast Random image compression rates (variable image resolution) Random flip (horizontal and vertical) Random rotation (between -90 and 90) Random hue saturation value Random Gaussian noise Random clip limits in the Contrast limited adaptive histogram equalization^54^
Model 1	5×, 10×, 20×	
Model 2	10×, 20×	
GP4		
Model 1	10×, 20×	
Model 2		
Model 3		
Model 4		
Model 5		
Model 6		
GP5		
Model 1	10×	
Model 2		

Various approaches for patch augmentation were applied to increase the variation in patch appearance to increase the likelihood of obtaining more generalizable models.

**Table 8:** The optimization set for Gleason pattern detection used to select the best models according to the best area under the curve (AUROC).

Finding	Case, n (%)	Regions, n (%)	Patches, n (%)
GP3	12 (21.4)	32 (6.0)	923 (11.1)
GP4	9 (16.1)	166 (31.0)	1318 (15.9)
GP5	20 (35.8)	216 (40.2)	5397 (65.0)
Normal tissue	11 (19.6)	106 (19.8)	466 (5.6)
HGPIN	4 (7.1)	16 (3.0)	204 (2.4)

This optimization set was curated from The Cancer Genome Atlas images with demarcation of 536 prostate cancer heterogeneous regions representing different Gleason patterns and benign tissues in 35 patients (~ 15 regions per patient) by a team of a senior pathologist and a prostate cancer researcher. These patches (512 × 512 pixels) were curated at the 10 × magnification level, resulting in a total of 8308 patches. The “Normal tissue” category covers the prostatic epithelium, stroma, and atrophy. HGPIN stands for high-grade prostatic intraepithelial neoplasia and is the presumed precursor of prostate cancer.

**Table 9:** The training sets used to develop patch-level classification models for the cribriform pattern, ductal morphology, high-grade intraprostatic intraepithelial neoplasia (HGPIN), vessels, nerves, and inflammatory cell infiltration.

Finding	Patches				
	Regions, n (case, n)	Magnification level	Positive, n (%)	Negative, n (%), (regions, n; cases, n)	List of negative findings (%)
Cribriform pattern	117 (13)	10×	546 (24.5)	1686 (75.5) (672; 29)	Gleason pattern 3 (45.0) Nerves (35.3) Normal glandular tissues (11.4) Gleason pattern 4 No Cribriform pattern (8.3)
Ductal morphology	83 (19)^+^	10×	278 (12.7)	1795 (87.3) (218; 21)	Gleason pattern 3 (51.4) Gleason pattern 4 (48.6)
HGPIN^++^	40 (4)	10×	248 (3.0)	5009 (97) (1564,37)	Gleason pattern 3 (9.2) Gleason pattern 4 (13.3) Cribriform pattern (11.6) Nerves (16.1) Vessel (12.7) Ductal adenocarcinoma. (8.9) Normal tissues (5.4) Inflammatory cell infiltration (22.3) Perineural invasion. (0.5)
Blood vessels	232 (23)	10×	375 (10.3)	3280 (89.7) (955; 42)	Gleason pattern 4 (28.2) Gleason pattern 3 (22.8) Nerves (18.1) Cribriform pattern (16.8) Ductal adenocarcinoma (8.0) Normal glandular tissues (6.1)
Nerves	344 (20)	10×	389 (11.2)	3088 (88.8) (545; 31)	Gleason pattern 4 (28.9) Gleason pattern 3 (23.9) Cribriform pattern (18.5) Blood vessels (14.6) Ductal adenocarcinoma (8.0) Normal glandular tissues (6.0)
Inflammatory cell infiltration	418 (25)	10×	430 (10.5)	3663 (89.5) (867; 35)	Gleason pattern 4 (24.7) Gleason pattern 3 (20.0) Nerves (16.5) Cribriform pattern (15.5) Blood vessels (12.6) Ductal adenocarcinoma (7.0) Normal glandular tissues (3.6)

A total of 42 patients and 1723 regions were considered. For all the findings, we oversampled the positive patches to overcome class imbalance. + 10 additional images were collected from the internet because ductal adenocarcinoma is a rare type of prostate cancer. Given that benign prostatic hyperplasia may have a glandular structure with a large lumen, we explicitly excluded patches from the lumen. Gleason patterns and normal glandular tissues further included stromal components. Normal tissues cover the ductus deferens and epithelial and stromal components from the peripheral and central zones of the prostate. Gleason pattern 5 was not included in the training set because this pathology was not detected in the optimization set to identify the ideal model. The initial HGPIN model was retrained on a training set that included an additional 4143 patches with prostatic hyperplasia from 19 lesions of 4 patients. Here, we used a learning rate of 1e-6 to avoid a complete distortion of the initial weights^55^.

**Table 10:** The optimization sets used to select the best models for high-grade intraprostatic intraepithelial neoplasia, vessel, nerve, and inflammatory cell infiltration.

Finding	Regions, n (case, n)	Magnification level	Positive, n (%)	Negative, n (regions, n; cases, n)	List of negative findings (%)
Cribriform pattern	87 (12)	10×	197 (32.9)	402 (67.1) (197, 25)	Gleason pattern 3 (41.0) Gleason pattern 4 without cribriform pattern (9.4) Nerve (36.7) Normal glandular tissues (12.9)
Ductal morphology	36 (1)	10×	59 (3%)	1906 (97%) (496, 42)	Gleason pattern 3 (8.3) Gleason pattern 4 (11.4) Gleason pattern 5 (56.1) Nerve (8.6) Blood vessel (6.8) Cribriform pattern (6.9) Normal glandular tissues (1.9)
HGPIN	16 (7)	10×	204 (2.5)	8104 (97.5) (581, 35)	Benign Prostatic Hyperplasia (5.8) Gleason pattern 3 (11.4) Gleason pattern 4 (16.2) Gleason pattern 5 (66.6)
Blood vessel	96 (18)	10×	123 (6.3)	1820 (93.7) (447,40)	Gleason pattern 3 (9.6) Gleason pattern 4 (10.9) Gleason pattern 5 (57.8) Nerve (8.2) Cribriform pattern (7.9) Normal glandular tissues (2.2) Ductal adenocarcinoma (3.4)
Nerve	147 (17)	10×	157 (8.0)	1812 (92.0) (392,41)	Gleason pattern 3 (10.2) Gleason pattern 4 (12.7) Gleason pattern 5 (56.8) Cribriform pattern (6.7) Vessel (6.3) Normal glandular tissues (3.1) Ductal adenocarcinoma (4.2)
Inflammatory cell infiltration	109 (22)	10×	161 (7.8)	1904 (92.2) (511, 42)	Gleason pattern 3 (10.0) Gleason pattern 4 (11.4) Gleason pattern 5 (54.1) Nerve. (7.3) Cribriform pattern (6.5) Vessel (5.5) Normal glandular tissues (1.7) Ductal adenocarcinoma (3.5)

These datasets were curated from the PRAD-TCGA histology image dataset. We emphasize that we added images representing Gleason pattern 5 as an unseen pathological finding. A total of 42 additional patients were considered for the optimization set. For the HGPIN, we increased the patch number to increase.

For precursor detection, cancer morphology detection and mesenchymal structure detection, we intentionally defined Gleason pattern 5 and benign prostatic hyperplasia as unseen pathological findings and included patches of these findings in the optimization datasets. The aim of this strategy was to increase the likelihood of detecting the best and best-fit models.

### Comparison with state-of-the-art model architectures

As an exploratory analysis to determine the optimal model architecture and to justify our model architecture selection, a comparison of our novel model architecture with the state-of-the-art model architectures was conducted on 60 randomly selected whole-mount images from Stanford to detect tiles with prostate cancer (a patch 512 × 512 pixels and corresponds to ~ 512 × 512 µm at 10 × objective magnification). We compared the accuracy of these methods on nonoverlapping patches generated from these images, as patches represent the smallest data unit on which different detection tasks depend. The models were trained using categorical cross-entropy loss with a batch size of 16 and optimized using “ADAM” with a default configuration^56^ and a learning rate of 1e-3. The patch augmentation incorporated random rotation, JPEG compression rates for random image resolution, flipping and color shifting as well as zooming. For all procedures, we utilized the same random seed (seed = 1234) to ensure that patch augmentation was similarly applied for all models. To account for variability in development set portioning, we partitioned the development set threefold and repeated our evaluation three times on the held-out test set. At each time point, we evaluated the area under the receiver operating characteristic curve (AUROC), expected classification error, and Brier score for patches per slide and then determined the mean and 95% confidence interval (CI) using 100,000 bootstrapped slide resamplings. The expected classification errors (ECEs) and Brier scores provide insights into the model goodness of fit and the model calibration. The lower the ECE or Brier score is, the better the goodness of fit and calibration of the model. On the basis of our evaluation, we determined that our novel model “PlexusNet” achieved performance comparable to that of state-of-the-art models widely used in medical imaging, while its parameter capacity was 150- or 85-fold lower than that of ResNet-50v2 or VGG16; moreover, the per-batch training duration for PlexusNet was at least two times shorter (223 ± 54 ms per batch vs. 630 ± 67 ms per batch for RestNet-50v or 528 ± 68 for VGG-16) on a single graphics processing unit (GPU) (NVIDIA™ Titan Volta with 12 GB) under similar data input/output conditions (one training process at a time and running processes relevant for the operating system). The results of the comparison analysis are summarized in Supplementary file 1.

### Hyperparameter configuration for model training

Table 11 provides the hyperparameter information used during model training. Models with the lowest AUROC in the optimization cohort were considered. For supervised contrastive learning, we selected the model with the lowest loss value in the steps for contrastive feature learning^53,57^.

**Table 11:** The final hyperparameter configuration determined according to the trial-and-error approach and grid search that resulted in examining 100 models.

Model	Loss function	Batch size	Optimization algorithm	Best epoch** (Max epochs)	Initial learning rate	AUROC *
GP3						
Model 1	Categorical cross entropy	16	For the first 100 epochs: ADAM For the remaining epochs: Weighted Stochastic gradient descent (wSGD) with decoupled weight decay^58^ for the remaining epochs (Weight decay = 1e-6, Decay steps = 1000, Decay rate = 0.5, momentum = 0.9)	70 (200)	For ADAM: 1e-3 For wSGD: 3e-5	0.934
Model 2				49 (100)		0.887
GP4						
Model 1 (SCL)	Max. margin contrastive loss for SCL^53^ Binary cross entropy for classification	16	[Contrastive feature learning] ADAM with cosine decay for learning rate (decay step: 1000) 16 [Classification] For the first 100 epochs: ADAM 16 For the remaining epochs: Weighted Stochastic gradient descent (wSGD) with decoupled weight decay^58^ (Weight decay = 1e-4, Decay steps = 50, momentum = 0.9) We also applied exponential decay to the learning rate (Decay steps: 50, decay rate: 0.5)	41 (200)^+^	[Contrastive feature learning] 1e-5 [Classification] For ADAM: 1e-3 For wSGD: 3e-5	0.781^+^
Model 2 (SCL)				110 (200)^+^		0.816^+^
Model 3	Categorical cross entropy			90 (200)		0.801^+^
Model 4				191 (400)^+^		0.805^+^
Model 5				393 (400)^+^		0.821^+^
Model 6				298 (300)		0.758
GP5						
Model 1	Categorical cross entropy	32	ADAM A class weight was applied instead of oversampling approach. The class weight is the inverse class proportion 0.7927398 for positive class (i.e., Gleason pattern 5) 0.2072602 for negative class	192 (300) ^+^	1e-3	0.996
Model 2				289 (300) ^+^		0.994
HGPIN	Categorical cross entropy	*16*	ADAM	31 (50)	1e-3	0.982
Cribriform pattern	Categorical cross entropy	32	ADAM	476 (600)	1e-3	0.943
Ductal morphology	Categorical cross entropy	64	Stochastic Gradient decent with the cycle polynomial decay learning rate (Decay step: 12,550, momentum = 0.0, power = 2, max. learning rate of 9.9725e-9)	28 (200)	1e-3	0.948
Blood vessel	Categorical cross entropy	128	ADAM	100 (200)	1e-3	0.940
Nerves	Categorical cross entropy	128	ADAM	105 (200)	1e-3	0.921
Inflammatory cell infiltration	Categorical cross entropy	128	ADAM	100 (100)	1e-3	0.926

**The epochs of models that we considered final models during the development setting. The AUROC was determined for the optimization cohort. Models were selected based on their AUROC

^+^Highlights that we consider two models originating from the same training setting. The reasons for considering multiple models are described in the following section.

### Ensemble learning for Gleason pattern detection

Given that Gleason patterns are heterogeneous and may have different latent appearance distributions, we applied ensemble learning to improve pattern detection accuracy and to increase the likelihood of having models robust to variation in appearance. Furthermore, we hypothesized that having multiple small models trained with different architectures and deep learning approaches provides better generalizability than does having a single small model.

### Gleason pattern 3

For Gleason 3, we trained two models with different model architecture configurations (see Table 11) on a dataset with magnification levels of 5 × , 10 × , and 20 × and one model on a dataset with magnification levels of 10 × and 20 × . The rationale behind training on such datasets is to capture magnification-invariant features related to Gleason pattern 3; specifically, we were interested in the well-differentiated glandular appearance and the connective tissue space between glandular structures, as these features have an important role in discriminating Gleason pattern 3 from 4 or 5.

### Gleason pattern 4

Because Gleason pattern 4 has a broader tumor appearance than other Gleason patterns (i.e., 3 and 5), we used six PlexusNet models with different model configurations and training schemes: one model contained 3 transformer blocks after the last convolutional layer and before the full connection step; two models of two different epochs were trained using supervised contrastive learning^53^; two models of different epochs were trained in the same training setting; and one model was trained based on multitask learning. The purpose behind considering models from different epochs or with different algorithms is to hopefully lessen the effect of the latent distribution shift that may occur during model training and to increase the likelihood of capturing informative features. In our case, we selected two models where the epoch distance between these models corresponded to the half distance to the last epoch with the best model (e.g., the best model was found at 393 epochs; accordingly, we selected the next model at 191 epochs; both models were registered due to the reduction in loss values on the in-validation set compared to the prior epoch). The optimal hyperparameters were configured using the trial-and-error method for the detection of Gleason patterns.

### Gleason pattern 5

**Figure 10:**
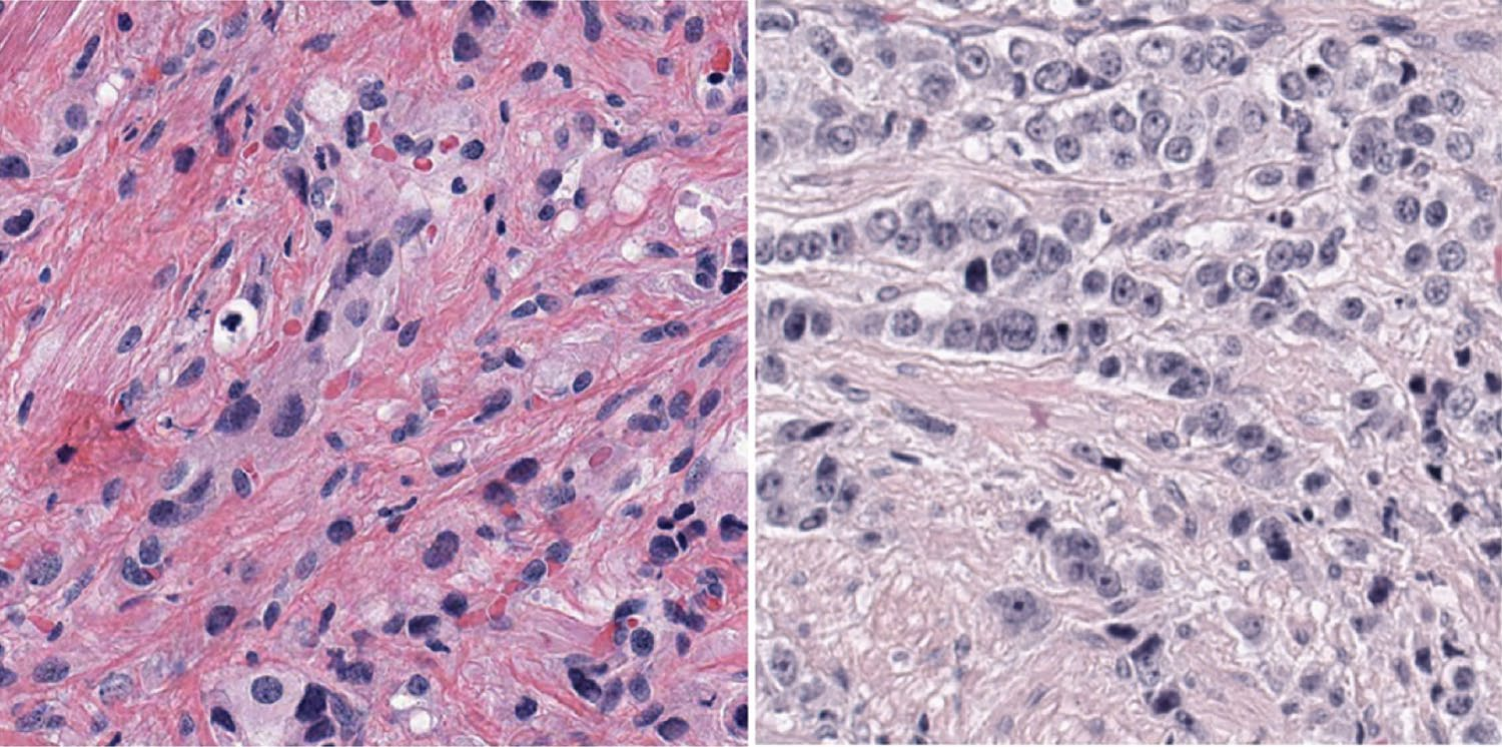
Shows two patches at 10 × objective magnification for Gleason pattern 5 (patch dimension: 512 × 512 px.).

For Gleason pattern 5, we utilized two models with soft attention blocks that generate pixelwise attention maps and trained on datasets of TMA spot images at a 10 × magnification. Our decision to use the pixelwise soft attention block was due to its superior performance for Gleason pattern 5 detection and histological characteristics of Gleason pattern 5 as part of the neural architecture search. As demonstrated in Fig. 10, the histological characteristics of Gleason pattern 5 include tumor cells infiltrating into the connective tissues (captured by local attention) and glandular structure disappearance (captured by global attention).

### Threshold selection

**Figure 11:**
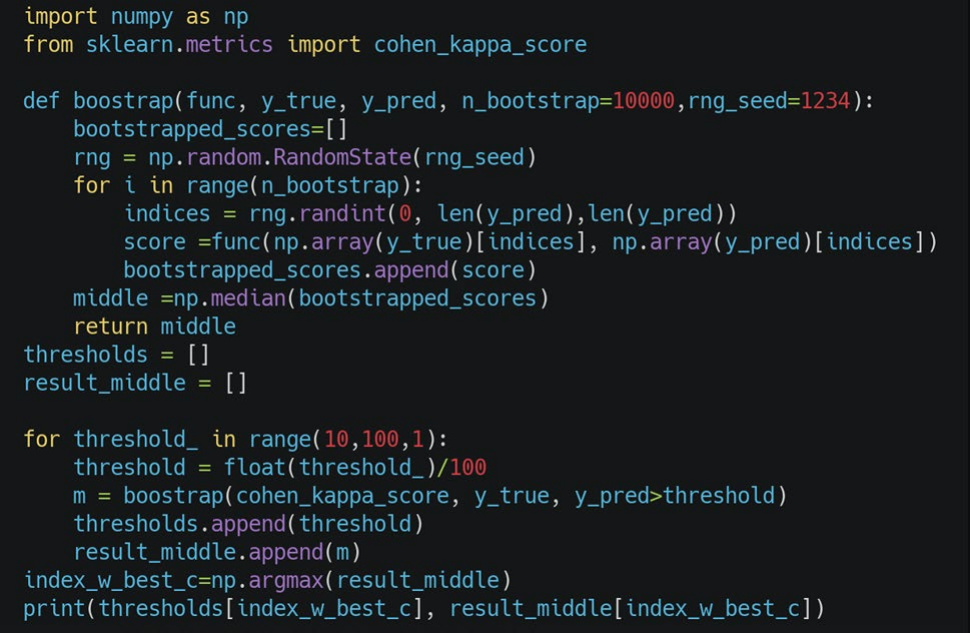
Illustrates the code snippet. This code determines the threshold with the highest Cohen kappa score. “y_true”: the ground truth labels of the patches from the optimization set, and “y_pred” is the predicted confidence score for these patches. We considered the median of the bootstrapped scores to mitigate the effects of outliers. After calculating the scores for all thresholds, the threshold with the highest Cohen kappa (index_w_best_c) was determined. The thresholds are given in the supplementary Materials and Methods section.

We applied the brute force approach to determine the threshold (operating point) with the best Cohen kappa on the in-training validation set for patch-level classification of findings. Here, we increased the initial threshold by 0.1 by 0.01 and measured Cohen kappa for interrater agreement between the annotator and vPatho at the patch level (tiles); our approach can be considered similar to that of Youden’s J statistics, where the largest J value is determined^59^. Bootstrapping with 10,000 resamplings was applied to determine the thresholds. Figure 11 provides a code snippet for threshold determination.

### Test conditions

To cover the full range of PCa evaluations performed during routine clinical practice, we defined eleven test conditions that are important for pathological evaluation and reports. The subset for each test condition was defined prior to running the test (Table 2 provides a summary of the datasets utilized for different test conditions). The eleven test conditions are as follows.

### Cancer detection on H&E-stained slides

We collected histological images that included slides archived for approximately 20 years to determine the influence of our approach on slides affected by the aging process. Spot images of a tissue microarray (TMA) and whole-mount images of prostatectomy specimens were also considered because they represent the breadth of tissue sampling for prostatic tissues.

Given that a single whole-mount (WM) image corresponds to approximately 30 stretched biopsy core images and given the time- and labor-intensive effort of obtaining high-precision annotations of WM images for prostate cancer, we randomly selected 46 radical prostatectomy specimens, for a total of 368 WM images [~ 11,040 biopsy core images with prostatic tissues or 894,240 patch images (512 × 512 pixels, 512 µm, at 10 × by a pixel mapping of ~ 1 µm per pixel] from 136 patients between 2016 and 2019.

A single pathologist (CK) annotated PCa areas on 368 slides. In contrast, each TMA spot was previously labeled for PCa presence based on the judgment of a single pathologist (RB) and whether it originated from cancer lesions in prostatectomy specimens. The cancer lesions in the historic McNeal slides were already delineated as part of a previous study investigating the tumor distribution during prostatectomy^32^. The use of different sample collections is reflective of real-world experience in clinical and research hospitals.

For the evaluation of agreement levels between automated and manual annotations of PCa, a 49% or above confidence score was used as the decision threshold for (integer) defining the presence of PCa in a patch. This threshold was deemed sufficient because of the well-calibrated DL model (see Supplementary file 1).

### Cancer detection on each H&E-stained slide

We measured the area under the receiver operating characteristic curve (AUROC) on every patch from every slide using their predicted confidence scores and their true labels for PCa presence. To account for variation in the number of patches between slides, we evaluated slide-level performance using the mean per-slide AUROC.

We also assessed Cohen’s kappa coefficient^60^ as a measurement of the degree of agreement between the model labels and the true labels made by the pathologist (CK) on image patches for each slide. Then, we determined the median per-slide Cohen kappa coefficient and its range. These test conditions reproduced the spatial PCa detection (annotation) on each slide as well.

### Cancer detection on H&E-stained slides from a single patient

In this test, all slides from the prostatectomy specimens of 46 patients with complete tumor annotations were utilized. We determined the average performance for cancer detection on patch images per patient (per-case AUROC). This test condition reflects the clinical condition of the pathology, where we collectively evaluated all the slides from a single patient.

### Spot detection of PCa on an H&E-stained TMA

This test task utilized H&E-stained histology images from 4 TMAs with a total of 1129 spots from 339 prostatectomy specimens; these images included, on average, three cores from each patient and contained TMA cores with normal tissues. Each spot image represents a single tissue core (diameter range: 0.6–1 mm). We determined the detection accuracy (i.e., sensitivity, specificity, negative and positive likelihoods, positive and negative predictive values and AUROC) for cancer detection on all spot images and the average AUROC after stratification by TMA (per-TMA AUROC). This test condition represents a research condition where the TMA evaluation of cancer presence is needed or when we have a very limited tissue amount to investigate for cancer presence during routine clinical practice or research. In parallel, we measured the impact of overlapping neighboring patches on the detection performance.

### PCa detection on old slides with weak H&E staining due to the aging process

We randomly selected 13 H&E whole slide images originating from the historic McNeal dataset^32^. These H&E slides were archived over a period of more than 20 years. From these slide images, we generated 11,862 patch images (512 × 512 pixels, 512 µm, at 10 × by a pixel mapping of 1 µm per pixel), including 3552 patch images with PCa. We applied a reference-free version of Macenko’s stain normalization algorithm for color intensity optimization of the patch images^43^ (see Fig. 1A.1).

For accuracy evaluation, we measured the area under the receiver operating characteristic curve (AUROC) on all patch images using their predicted confidence scores and their true labels for PCa presence. These test conditions replicate the challenge of cancer detection on images originating from slides with faded staining to assess the limitations of our approach.

### Tumor volume estimation

We measured the association between the predicted and true tumor volumes in 46 prostatectomy specimens with complete delineation of cancer lesions (368 slides). We utilized the well-established grid method, which was described in detail in our previous study^4^. In brief, we defined a two-dimensional space for each slide where a single patch image corresponded to a pixel (a pixel corresponded to a 512 × 512 µm dimension of a slide image at the 10 × objective magnification level). Then, we counted the total number of pixels (patch images) that were positive for prostate cancer. We also determined the background tissue using image thresholding (Otsu’s method) and excluded the white areas located outside the background tissues in all slides for each patient. After that, we determined the number of patch images generated from the background, which also affects the number of pixels. Finally, the total number of pixels with PCs was divided by the total number of pixels in the background tissues to estimate the tumor volume (TuVol%) in each patient (Fig. 1B). The coefficient of the regression score determined the correlation of TuVol% between the ground truth and the AI solution at the case level. The pairwise Welch's t test was applied to determine the significance of the difference between the ground truth and the AI solution for tumor volume estimation^61^.

### Gleason patterns and ISUP grading

This test condition involved three tasks. The first task is to determine the Gleason patterns on very limited tissues (~ 256–512 µm) suitable for laser capture microdissection^62^; the second and third tasks are to determine the ISUP grade on biopsy cores and prostatectomy specimens, respectively.

The ISUP grading system for each biopsy core was defined by the most frequent Gleason pattern for the primary Gleason pattern and the most common Gleason pattern for the secondary Gleason pattern. In contrast, the ISUP grading system for radical prostatectomy specimens considers the first and second most frequent Gleason patterns across the specimen. The second most common Gleason pattern is considered to indicate the secondary Gleason pattern if it is equal to or exceeds 5%. If the second most common Gleason pattern is less than 5%, the secondary Gleason pattern will be the same as the primary Gleason pattern, and the second most common Gleason pattern will be the tertiary Gleason pattern.

Since there are no widely agreed-upon rules for limited tissues to report ISUP grade and because the patch level is the smallest unit required to assess ISUP grade at the slide and case levels, we focused on evaluating the accuracy of Gleason pattern detection in such limited tissues. The decision thresholds of 35%, 65% and 93% for GP3, GP4 and GP5, respectively, were retrospectively determined on the development set using a brute force algorithm that identified thresholds with the best interrater reliability (Cohen’s kappa^60^). After these thresholds were fixed, the presence of the Gleason pattern was determined via patch images to measure interrater agreement.

Two test conditions were used to assess the concordance level between pathologists and vPatho for Gleason grading in the ISUP dataset (biopsy cores) and Stanford’s external dataset (radical prostatectomy). We aimed to use the same vPatho for these two different grading conditions to identify histopathological factors impacting the concordance level of the current ISUP grading system on radical prostatectomy specimens. We considered both 10 × and 20 × magnification levels to determine the magnification level with better Gleason pattern detection.

### Very limited tissue samples for laser microdissection

Nonoverlapping patch images covering GP3, GP4 and GP5 were obtained to simulate a Gleason pattern detection on tissue dimensions suitable for laser capture microdissection (Table 12). These images were generated from 47 prostate cancer regions randomly selected from 60 whole-mount slides of 10 radical prostatectomies (average 4.7 regions per radical prostatectomy). Each patch image included only one Gleason pattern owing to the time-intensive effort to annotate highly homogenous regions for each pattern. The labels of the patch images were defined based on the annotation data curated by a single pathologist, “YT”. A patch image is positive for one of the Gleason patterns when at least 10% of the patch image is positive. For each Gleason pattern, we measured the detection accuracy and interrater agreement between the pathologists and the DL models (see evaluation metrics). Finally, we repeated the evaluation at 20 × to evaluate the agreement between the DL models and the single pathologist (Table 12).

**Table 12:** Lists the numbers of patch images for each finding considered to evaluate our deep learning models for Gleason pattern detection on very limited tissue samples.

	Number of patch images (%)	
Finding	10 × objective magnification	20 × objective magnification
High-grade Prostatic intraepithelial neoplasia	250 (22.2)	845 (22.0)
Gleason pattern 3	311 (27.6)	1211 (31.5)
Gleason pattern 4	454 (40.2)	1374 (35.8)
Cribriform glands	36 (7.9)	117 (8.5)
Poorly formed glands	210 (46.3)	538 (39.2)
Fused glands	19 (4.2)	57 (4.1)
Poorly formed and fused glands	189 (41.6)	662 (48.2)
Gleason pattern 5 (Mixed)	113 (10.0)	410 (10.7)

Manual quality control was performed to ensure that these patches included only a single finding.

### Biopsy cores

We considered the reference image database for Gleason patterns (GPs) and Gleason grading provided by ISUP, an international organization responsible for the histopathological definition of Gleason grading^35^. These reference images were graded by the ISUP member team according to majority rule^35^. This database represents a unique and independent resource for evaluating the agreement between an expert panel and DL models. To determine the ISUP grade on biopsy cores, we considered reference images captured at 20 × objective magnification. By considering the different sizes of these images, we generated patches (512 × 512 µm; 1 pixel = 1 µm) for each ISUP image at 10 × objective magnification after downsizing the original histology images or (~ 256 × 256 µm; 1 pixel = 0.5 µm) at 20 × objective magnification. We populated the patch number for each ISUP image by applying 50% overlap between patches to increase the likelihood of a complete appearance of Gleason patterns in these patches with effective computational workloads. Then, we counted the positive patches for each GP. After implementing a conditional algorithm according to the ISUP grading system for the biopsy core, we estimated the ISUP grade for each biopsy core and evaluated the agreement level for tumor grading between the expert panel and our approach.

### Radical prostatectomy

A total of 136 patients who underwent radical prostatectomy were available for this task. These prostatectomy specimens were already evaluated during routine clinical practice by six different board-certified pathologists between 2016 and 2019. This cohort included patients seen during routine clinical practice. Using chart review, we acquired pathological and clinical information for each patient. The information included the tumor stage, the status of locoregional lymph node metastases, surgical margin status, the year of the pathology report, the ISUP grade and the pathologist who conducted the pathology evaluation.

After excluding the white background, all whole-mount slide images were tiled into 512 × 512 pixel patches (~ 512 µm). Then, we counted the number of positive patches for each GP. Finally, we developed and applied a conditional algorithm to grade PCa in each patient according to the International Society for Pennsylvania [ISUP] grading system for prostatectomy. The patch images did not overlap because of the greater dimension of the WM images than of the biopsy cores.

We measured the agreement between the results of a pool of 6 pathologists who evaluated these patients during routine clinical practice and our approach. We repeated our agreement evaluation after we adjusted the decision threshold from 5 to 10% for secondary and tertiary Gleason patterns since our previous study showed that eyeball judgment can underestimate the tumor size by 50% of the original size^4^.

### Sorting the slides according to cancer presence status

We wanted to determine the sorting accuracy of slides with PCa since this measurement is relevant for improving clinical workflows. Therefore, we considered different types of slides and datasets to verify the model performance in identifying slides with PCa (Table 2). These slides originated from different institutions and used different protocols for tissue preparation or from different backgrounds (i.e., lymph node tissues). Here, two positive patches (in which the tumor probability exceeded the threshold of 49%) were enough to mark the slide as positive***.*** We assessed the confusion matrix and measured the true positive rate (TPR), positive predictive value (PPV), true negative rate (TNR) and negative predictive value (NPV) to objectively evaluate the sorting accuracy.

### Detection of ductal morphology

A total of 1247 nonoverlapping patch images (512 × 512 pixels ~ 512 × 512 µm at 10 × objective magnification) covering ductal adenocarcinoma were obtained to simulate ductal morphology. These patches were generated from 38 prostate cancer regions randomly selected from 2 whole-mount slides of 2 radical prostatectomies (Table 13). Since ductal adenocarcinoma is a rare cancer morphology (approximately 0.4–0.8% of radical prostatectomies), we weighted the patch number in favor of ductal adenocarcinoma for a balanced performance evaluation. The labels of the patch images were defined based on the annotation data curated by a single pathologist, “YT”. Each patch image covered a single entity owing to the time-intensive effort to annotate highly homogenous regions. Given that cancer lesions with ductal morphology may incorporate empty spaces, a patch image was considered positive for ductal morphology when at least 40% of the patch image was positive and the white background area in the patch image was not more than 60%. For ductal morphology, we measured the detection accuracy and interrater agreement between the pathologists and the DL model.

**Table 13:** Lists the numbers of patch images used to evaluate the detection model for ductal adenocarcinoma.

Finding	Number of patch images (%)
Ductal adenocarcinoma	1247 (59.0)
Nonductal prostate cancers	865 (41.0)
Gleason pattern 3	315 (36.4)
Gleason pattern 4	435 (50.3)
Gleason pattern 5	115 (13.3)

The definition of ductal morphology primarily depends on the ductal appearance of prostate cancer, and splitting a region with ductal adenocarcinoma of the prostate into patches causes the disappearance of ductal morphology in some patches. Therefore, we specifically reviewed and included only patches that represented their corresponding finding labels. The threshold was identified and set to 90% using the development set via the same approach for threshold determination for Gleason pattern detection, as described earlier.

### Detection of the cribriform pattern

A total of 92 nonoverlapping patch images (512 × 512 pixels; ~ 512 × 512 µm at 10 × objective magnification) covering cribriform Gleason pattern 4 (n = 37) or ductal adenocarcinoma with cribriform architecture (n = 58) were obtained to simulate a cribriform pattern (Table 14). The true label of patch images was defined based on the regional delineation and its labels on histology slides made by a single pathologist, “YT”. A single finding was obtained for each region to construct a more homogenous dataset to simulate the detection performance for cribriform patterns. A patch image was considered positive for the cribriform pattern when at least 50% of the patch image was positive. As a negative control, we considered a total of 836 patients with Gleason patterns 3 to 5, with the exception of patients with Gleason pattern 4 and cribriform patterns. These images for the negative control originated from the previous test conditions for GS detection.

**Table 14:** Lists the numbers of patch images used to evaluate the detection model for ductal adenocarcinoma.

Cribriform patterns	Number of patch images (%)
Yes	92 (9.9)
No	836 (90.1)

For the cribriform pattern, we measured the detection accuracy and the interrater agreement between the pathologist and the DL model. Given that the definition of the cribriform pattern primarily depends on the distinctive appearance of holes within the cancer cell aggregation zone, we specifically reviewed these patches to ensure that the positive patches included the distinctive appearance of the hole in prostate cancer tissue prior to performing the evaluation. The threshold was identified and set to 65% in the development cohort using the same approach for threshold determination for Gleason pattern detection, as described earlier.

### Detection of nerves

A single pathologist randomly delineated 158 nerve structures on the same 60 slides of 10 patients, resulting in a total of 739 patch images with nerve components (nerves and ganglions). Furthermore, we considered 541 patches with Gleason patterns as the negative control group, as these images did not include nerve structures. A patch image was considered positive for nerve structures when at least 5% of the patch image was positive for the incorporation of small nerve structures (~ 25.6 µm). Finally, we measured the detection accuracy and interrater agreement between the pathologists and vPatho. Given the heterogeneity of the prostatectomy specimens, we specifically reviewed these patches to ensure that the patches represented their corresponding labels prior to evaluation. A probability threshold of 35% was identified on the development set and fixed for testing using the same approach for the threshold determination described earlier.

### Detection of vessels

We generated a total of 1455 nonoverlapping patch images (512 × 512 pixels; 512 × 512 µm at 10 × objective magnification) covering the general appearance of blood vessels to simulate a blood detection task. These images were generated from 150 blood vessels of various sizes randomly selected and annotated by a single pathologist on the previous 60 whole-mount slides of 10 radical prostatectomies. A patch image was considered to be positive for the blood vessel when at least 5% of the patch image was positive (a threshold of 5% was considered to cover small vessels). The negative group included 3153 patches from 50 cancer regions that were already prepared in the previous steps. Finally, the detection accuracy and the interrater agreement between the pathologist and the DL model were calculated. The probability threshold for classifying the patches was set to 50%.

### Detection of inflammatory cell infiltration

We curated 268 nonoverlapping patches with inflammatory cell infiltration (512 × 512 pixels; 512 × 512 µm at 10 × objective magnification) to simulate the detection of tissue regions with infiltrated inflammatory cells. In parallel, a negative group of 500 patch images positive for cancer was randomly generated from 216 regions. A patch image was considered positive for inflammatory cell infiltration when at least 3% of the patch image was positive since inflammatory cell infiltration can be small (~ 10 µm). Thereafter, we measured the detection accuracy and interrater agreement between the pathologists and the DL model. Given the heterogeneity of the prostatectomy specimens, we specifically reviewed these patches to ensure that the patches represented their corresponding labels. We used a probability threshold of 70.5% to classify the patches, which was determined on the development set using the same approach described earlier.

### Detection of tumor precursors

A single pathologist screened for high-grade prostatic intraepithelial neoplasia (HGPIN) lesions in 60 whole-mount slides of 10 radical prostatectomy patients and identified 32 regions with HGIPN. From these regions, we curated 250 patches to examine the detection performance for HGPIN with 50% overlap (512 × 512 pixels; 512 × 512 µm at 10 × objective magnification) to increase the likelihood of having an HGPIN appearance in these patches. While generating the patches, we manually excluded the lumen patches after running an algorithm that identified and excluded the lumen patches as background. A single patch is positive when 10% of the patch is positive for HGPIN (this threshold is arbitrary). As a negative control, we used patches with benign prostatic hyperplasia (BPH) and intraductal adenocarcinoma (IDC) because IDC is considered the differential diagnosis of HGPIN, whereas BPH and HGPIN are benign tumor-like lesions in the prostate^57,63–65^. While generating the patches, we manually excluded the lumen patches after running an algorithm that identified and flagged the lumen patches as background. The test conditions include the detection of HGPIN in limited tissues where capturing HGPIN lesions requires the use of laser microdissection. Identifying regions suitable for laser microdissection is also essential for studies investigating HGPIN lesions. We emphasize that our sample size for HGPIN patients is adequate given that HGPIN lesions are mostly limited and very small lesions are found in up to 50% of prostatectomy specimens^66^ and therefore preferably sampled using laser microdissection^67^. The probability threshold for classifying the patch according to the presence of HGPIN was fixed at 65%, which was identified using the brute-force search approach on the development set, as described in the supplementary model development section.

### Reporting the existence of relevant findings in a case

We aimed to evaluate the feasibility of integrating the results from deep learning models into pathology reports. Here, we aimed to answer two major questions:

1. How integrable are the results provided by vPatho into the pathology report for prostatectomy specimens?
2. What information needs to be considered when implementing such results in pathology reports?

To answer these questions, we selected 136 patients who underwent radical prostatectomy, and the data of these patients were obtained during routine clinical practice. Since these prostatectomy specimens were already evaluated during routine clinical practice and since the evaluation results were documented, we reviewed the pathology reports for the ISUP grade, tertiary Gleason pattern, cribriform pattern, and ductal morphology for these patients before running the analyses.

### Investigating variables indicating the degree to which the prostatectomy specimens were discordant

The following analyses were performed to answer the question of whether the current practice of grading prostatectomy specimens with ISUP has a direct effect on grade disagreement (discordance) after controlling for other confounders. We further wished to identify other independent factors associated with grade disagreement in prostatectomy specimens.

Our study involved experienced genitourinary pathologists from high-volume centers (> 200 radical prostatectomy cases per year) to compare our AI solutions. A survey revealed a significant correlation between the number of biopsies evaluated by a pathologist per week and the grade concordance level with expert review^68^. Goodman et al. concluded that the likelihood of discordance between pathology reports and expert-assigned Gleason scores is particularly elevated for small community hospitals compared to high-volume hospitals^69^. Therefore, defining expertise based on case volume instead of experience years (which is associated with a physician group who is at higher risk for medical errors due to their age^70^) is the most appropriate reflection of clinical experience.

**Figure 12:**
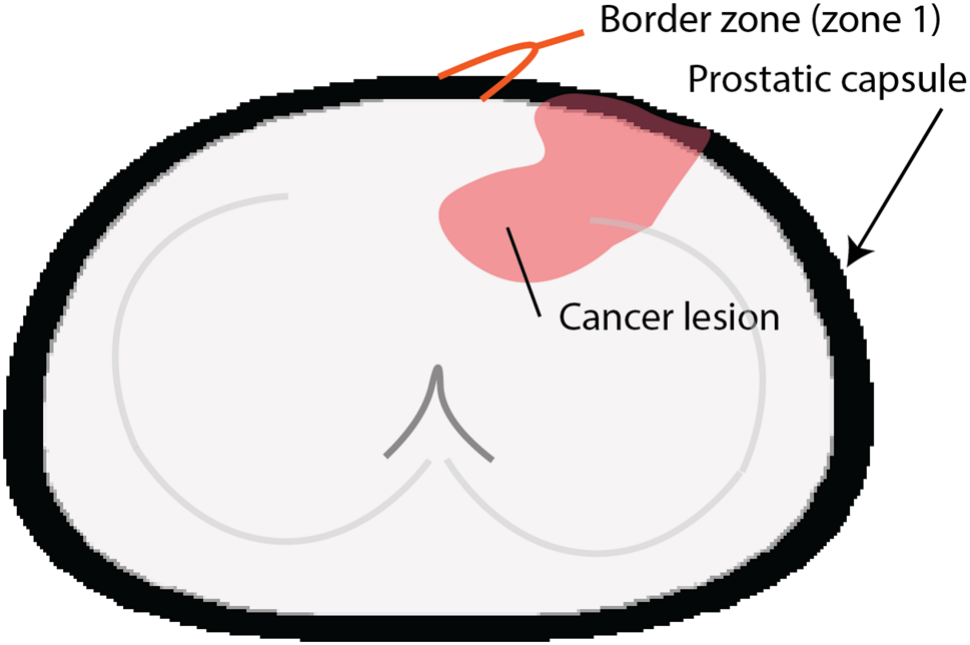
Schematically illustrates the definition of the border zone (zone 1), which comprises 10% of the prostatic slice area and is adjacent to the prostatic capsule.

We investigated the effects of these variables on the degree of grade disagreement between the pathology reports and our approach using the binomial generalized linear mixed-effects model^71^. The variables were the pathologists who conducted the pathology evaluation, TuVol% made by AI and pathologists, the proportion of positive slides (which represents the vertical tumor extent), the cancer proximity to the prostatic capsule and grades (i.e., the ISUP grade made by pathologist and by AI-assisted approach), the year of tumor grading, and the total number of slides for each case. The proximity of the cancer to the prostatic capsule was determined using a mask function that considers only cancer areas within a border zone (zone 1) covering 10% of the prostate slice, as schematically illustrated in Fig. 12, and the registration of cancer presence was performed according to Eminaga et al.^72^.

The year of tumor grading was incorporated as a representation of the variation in the staining protocol over the time and adaptation period of the recent version of the ISUP grading system in 2016. As model input, the standard scores were estimated for all the variables listed above. As a model output, we calculated the odds ratio (OR) and its 95% Wald confidence interval (WCI) for each variable.

We designed four mixed-effects models to identify the effects of these variables on grade disagreement with four goals:

1. The effect of the ISUP grade determined by pathologists on grade discordance was evaluated regardless of the pathologist who provided the tumor grade.
2. The effect of the pathologist on grading disagreement, regardless of the ISUP grade, was evaluated
3. The predictive ability of variables that are determined by the AI for grade disagreement was evaluated regardless of the pathologist who assessed the tumor grade.
4. The present study evaluated the ability of significant variables to predict cancer proximity to the prostatic capsule, tumor stage, and locoregional lymph node metastasis status in prostate cancer patients.

The hypotheses for each model were as follows:

#### H0:

There is no relationship between the variables and grade disagreement.

#### H1:

There is a relationship between one or more variables and grade disagreement.

Since multicollinearity may impact the stability of the generalized linear mixed-effects model, we assessed the variance inflation factor (VIF) of these variables to measure multicollinearity in each model. Here, we considered a VIF less than 2 to indicate negligible collinearity^29^. The intraclass correlation coefficient was calculated for the random effects to determine the proportion of variance that can be explained by the random effects^73^.

We further evaluated the impact of each variable on the goodness of fit of each model by comparing the Akaike information criterion (AIC)^74^ of the model before and after excluding the variable; the analysis of deviance tables was computed to determine the comparison significance between a model with the variable and a model without the variable. The false discovery rate (used to correct *P* values) was estimated to determine the significance of the change in AIC.

We run a Monte Carlo simulation (1000 simulations) to measure the power of the effects for all significant variables after setting the percentage of type I errors (falsely rejecting the hypothesis H0 that there is no relationship) to 10% (or 5% for each side). The function “powerSim” from the “simr” package was used to measure the power of the mixed-effects models^75^.

Given that there is collinearity in the ISUP grades between the AI and pathologists, we considered only the ISUP grades assigned by pathologists for goals 1, 2 and 4 and the ISUP grades assigned by the AI for goal 3. In goal 4, we further repeated our model evaluation after replacing the ISUP grades made by pathologists with those made by the AI. Table 15 reveals the variables for fixed effects and random effects. The “glmer” function from the lmer4 package was used for binomial logistic linear mixed-effects modeling with the default configuration^76^.

**Table 15:** Describes the variables included in each mixed-effects model.

	Variables	
Model (goal)	Fixed effects	Random effect (random intercept)
A	Tumor volume in percentage Year of the pathology report Proportion of positive slides The total number of slides Gleason grade made by the pathologists	Pathologists
B	Tumor volume in percentage Year of the pathology report Proportion of positive slides The total number of slides Pathologists	Gleason grade made by the pathologists
C	Tumor volume in percentage Year of the pathology report Proportion of positive slides Gleason grade made by AI	Pathologists
D	Tumor stage (pT) Locoregional lymph node metastases status (pN) Cancer proximity to prostate capsule Gleason grade made by the pathologists/by AI Tumor volume in percentage Proportion of positive slides	Pathologists

The general equation for the mixed-effect model is

$$1. \,y=X\beta +Z\upsilon + \epsilon$$

where y is a vector of the grade disagreement status, $X$ is a matrix of fixed variables, $\beta$ is a vector of fixed effects regression coefficients, Z is a design matrix of the random effect, $\upsilon$ is a vector of the random effect and $\epsilon$ is a vector of the residual.

Finally, we conducted model-based causal mediation analyses to measure the indirect causal mediation effect of the pathologist or another significant variable (other than ISUP grade) on grade disagreement. To simplify the mediation analyses, we defined low-grade and high-grade tumors based on the ISUP grade (ISUP grades 1 and 2 vs. ISUP grades 3–5) as a binary group instead of considering the five ISUP grades.

We further evaluated the associations of the variables with the mediators. When we investigated the factor of interest as a mediator, we selected variables that were defined based on the pathologist’s decision (tumor stage, pN status, and ISUP grading). Furthermore, we incorporated TuVol%, the proportion of positive slides and the year of tumor grading as these factors may affect the identification of WM histology slides. Such associations were assumed when the *P* value was < 0.2 (two-tailed test).

A sensitivity analysis with 1,000 bootstrap resamplings was carried out to validate the results for the causal mediation effects after iterating the mediating coefficient with different constants^77^.

The mediation and sensitivity analyses were conducted using the package “mediation”^77^; the moderation of the relationship between the independent variables and the mediator was assessed using a linear model, whereas the overall effect was estimated using a binomial logistic regression model^78^. The results of the mediation analyses are presented in flowchart diagrams.

The contingency table was evaluated using Pearson’s chi-squared test^79^. The medians were evaluated for differences between the groups using the Mann‒Whitney Wilcoxon test^80^, and the means were compared between the groups using Welch’s t test^61^. A *P* value ≤ 0.05 indicated a significant difference. The 95% Clopper–Pearson confidence interval for the proportion was estimated^81^.

### Evaluation metrics

The discrimination accuracy for each endpoint was evaluated using the AUROC. The AUROC reveals the classification performance at different thresholds; a higher AUROC indicates a better classification accuracy, where an AUROC of 1 represents the highest accuracy^82^. Using the thresholds determined on the development set, we calculated the sensitivity, specificity, and negative and positive likelihoods. Furthermore, we estimated the positive and negative predictive values for different prevalence rates (e.g., patch proportion with prostate cancer). The agreement rate was measured using Cohen kappa for binary categories or weighted quadratic kappa for more than 3 categories. The agreement rate was determined according to Cohen J., who introduced Cohen kappa and weighted kappa (Table 16)^60,83^.

**Table 16:** Lists the kappa values and the corresponding agreement levels.

Kappa	Agreement
< 0.0	Less than chance agreement
0.01–0.20	Slight agreement
0.21–0.40	Fair agreement
0.41–0.60	Moderate agreement
0.61–0.80	Substantial agreement
0.81–0.99	Almost perfect agreement

The coefficient of the regression score determined the correlation of relative tumor volumes between the ground truth and the AI solution at the case level. The pairwise Welch’s t test was applied to determine the significance of the variation between the ground truth and the AI solution for tumor volume^61^. The reported *P* values are two-tailed, and *P* ≤ 0.05 was considered to indicate statistical significance. The uncertainty (95% CI) for the area under the curve (AUROC), Cohen kappa coefficient (Cohen kappa) and weighted kappa coefficient was determined by bootstrapping with 100,000 replications^84^. The Cooper–Pearson interval was used to calculate the 95% confidence interval^81^ for detection accuracy (i.e., sensitivity, specificity, positive and negative likelihoods, and positive and negative predictive values).

### Software and hardware settings

Our analyses were performed using Python 3.6 (Python Software Foundation, Wilmington, DE) and R 3.5.1 (R Foundation for Statistical Computing, Vienna, Austria). We applied the Keras library, a high-level wrapper of the TensorFlow framework, to develop the models. All analyses were performed on a GPU machine with a 32-core AMD processor with 128 GB RAM (Advanced Micro Devices, Santa Clara, CA), 2 TB PCIe flash memory, 5 TB SDD hard disks, and a single NVIDIA Titan V GPU with 12 GB VRAM.

### Supplementary Information

Supplementary Information 1.

Supplementary Table S5.

## Data Availability

Stanford’s datasets are not publicly available due to patient privacy regulations and the internal data sharing policy. However, other public datasets are available, and their sources are given in the “image database” section (Supplementary material). A cMDX file and the cMDX viewer tool are available for demonstration purposes (https://github.com/oeminaga/cmdx_report.git), and a python package for the PlexusNet architecture is publicly available (https://github.com/oeminaga/PlexusNet.git).

## Code statement

An abstract version of the pipeline will be provided upon acceptance.
