# Supplementary material for: Critical evaluation of artificial intelligence as a digital twin of pathologists for prostate cancer pathology
Source: Sci Rep. 2024 Mar 4;14:5284. doi: 10.1038/s41598-024-55228-w (PMC10912767; doi:10.1038/s41598-024-55228-w)
Supplement: Supplementary file 1 — Supplementary Information 1. [file 41598_2024_55228_MOESM1_ESM.pdf]

Supplementary results and discussion section for the manuscript “Critical Evaluation of Artificial Intelligence as a Digital Twin of Pathologists for Prostate Cancer Pathology”

Table of Contents

SLIDE AND IMAGE QUALITY MANAGEMENT ... **2**
STAIN COLOR RESTORATION FOR CANCER DETECTION ... **3**
WHEN AND WHEN NOT TO USE VIRTUAL PATHOLOGIST ... **4**
PROSTATE CANCER ... **8**
TMA SPOT SORTING ACCORDING TO CANCER PRESENCE ... 8
SORTING HISTOLOGY SLIDE IMAGES ACCORDING TO CANCER PRESENCE ... 12
DETECTION OF LYMPH NODE METASTASES ... 15
TUMOR VOLUME ESTIMATION ... 16
GLEASON PATTERNS AND ISUP GRADING ... **17**
CLASSIFICATION PERFORMANCE ... 17
THE ERROR DISTRIBUTION CROSS GLEASON PATTERNS ... 22
ISUP GRADING FOR RADICAL PROSTATECTOMY ... 22
FACTORS ASSOCIATED WITH DISAGREEMENT IN ISUP GRADING ... **24**
TO EVALUATE THE INDICATIVE ASSOCIATION OF THE ISUP GRADING MADE BY THE PATHOLOGISTS ON THE GRADE DISAGREEMENT REGARDLESS OF THE PATHOLOGIST WHO PROVIDED THE TUMOR GRADING. ... 27
TO EVALUATE THE INDICATIVE ASSOCIATION OF THE PATHOLOGIST ON THE GRADE DISAGREEMENT REGARDLESS OF THE ISUP GRADE MADE BY THE PATHOLOGISTS ... 27
TO EVALUATE THE INDICATIVE ASSOCIATION OF VARIABLES THAT ARE DETERMINED BY AI ON THE GRADE DISAGREEMENT REGARDLESS OF THE PATHOLOGIST WHO CONDUCTED THE TUMOR GRADING THAT WE COMPARED WITH. ... 27
TO EVALUATE THE INDICATIVE ASSOCIATION OF SIGNIFICANT VARIABLES ON DISAGREEMENT WHILE CONSIDERING THE TUMOR STAGE AND THE STATUS FOR LOCOREGIONAL LYMPH NODE METASTASIS STATUS OF PROSTATE CANCER. ... 28
MEDIATION ANALYSES ... 29
DETECTION OF CANCER MORPHOLOGY ... **30**
DETECTION OF MESENCHYMAL TISSUES ... **32**
DETECTION OF TUMOR PRECURSORS ... **33**
INTEGRATING VPATHO EVALUATION RESULTS INTO AN ELECTRONIC PATHOLOGY REPORT ... **36**
ACTIVATION MAPS ... **42**
ADDENDUM TABLE ... **43**
REFERENCES ... **47**

Notice:

This supplementary results section includes results and discussion not mentioned in the major manuscript for clarity reasons. The supplementary section uses the term "classification" and "detection" as well as "discordance" and "disagreement" interchangeably given that they have the same aim in this study which is to process patches to determine the presence of pathological finding or morphological appearances.

Slide and image quality management

In this study, we conducted a review of the histology slides for quality control prior to starting the study. Our inclusion criterion was whether the H&E staining features allowed for distinguishable representation of nuclei (basophilic) and cytoplasm (acidophilic) as well as between epithelial and connective tissues. As part of the Standard Operating Procedure for clinical routine, when a slide has poor preparation quality (e.g., poorly stained, or extremely distorted tissues), a new slide is prepared and stained for pathology evaluation. Similarly, when a histology image is of poor quality (e.g., blurred), the corresponding slide is rescanned. It is worth mentioning that all deep learning models were trained on patches augmented for magnification (zooming), color contrast, image resolution (i.e., JPEG compression) and brightness to account for variation in color brightness, contrast, and image quality.

We evaluated the impact of the automated illumination correction algorithm we implemented as part of image quality control on the prostate cancer detection using the Stanford's tissue microarrays (TMA) with 1,129 spots. These spots were covering the slide areas generally evaluated during the clinical routine and consequently represent the alteration in the illumination condition of the slide surface. The correction algorithm significantly improved the detection accuracy by 73.8% (95% CI: 62.5 – 86.8) from

ROCAUC of 0.521 (95% CI: 0.521 – 0.608) to 0.982 (95% CI: 0.974 – 0.988) on TMA spots, highlighting the relevance of the illumination and stain color justification for the detection performance.

Stain color restoration for cancer detection

Aging hurts the stain quality of slides stored for a long period of time due to chemical degradation and therefore must be accounted for. Our study recognizes that available color normalization algorithms can be cost-effective solutions for reconstructing and improving the stain quality of histology images from long-term archived slides to achieve a detection performance for prostate cancer comparable to the detection performance seen on histology images of recent slides. **Table S1** and S**2** detail our models' detection performance for prostate cancer at the patch level. Positive and negative predictive values were estimated according to proportion of patches with prostate cancer as shown in **Figure S1**.

Table S1: Confusion matrix for prostate cancer detection on patches originated from histology images of slides with faded staining.

	vPatho for prostate cancer detection on artificially improved staining		
	Number of Patches, (%) (512 × 512 μm at 10x magnification)		
Finding	Positive	Negative	Total
Prostate Cancer	3351 (94.3)	201 (5.7)	3552 (30.0)
Normal	1433 (17.2)	6877 (82.8)	8310 (70.0)
Total	4784 (40.3)	7078 (59.7)	11862 (100.0)

Table S2: Detection performance of vPatho for prostate cancer on patches originated from histology images of slides with paled staining. The original staining was proceed using a reference-free version of Macenko.

Detection accuracy			
Sensitivity, % (95% CI)	Specificity, % (95% CI)	Positive likelihood ratio (95% CI)	Negative likelihood ratio (95% CI)
94.3 (93.5– 95.1)	82.8 (81.9 – 83.6)	5.47 (5.22 – 5.74)	0.07 (0.06 – 0.08)

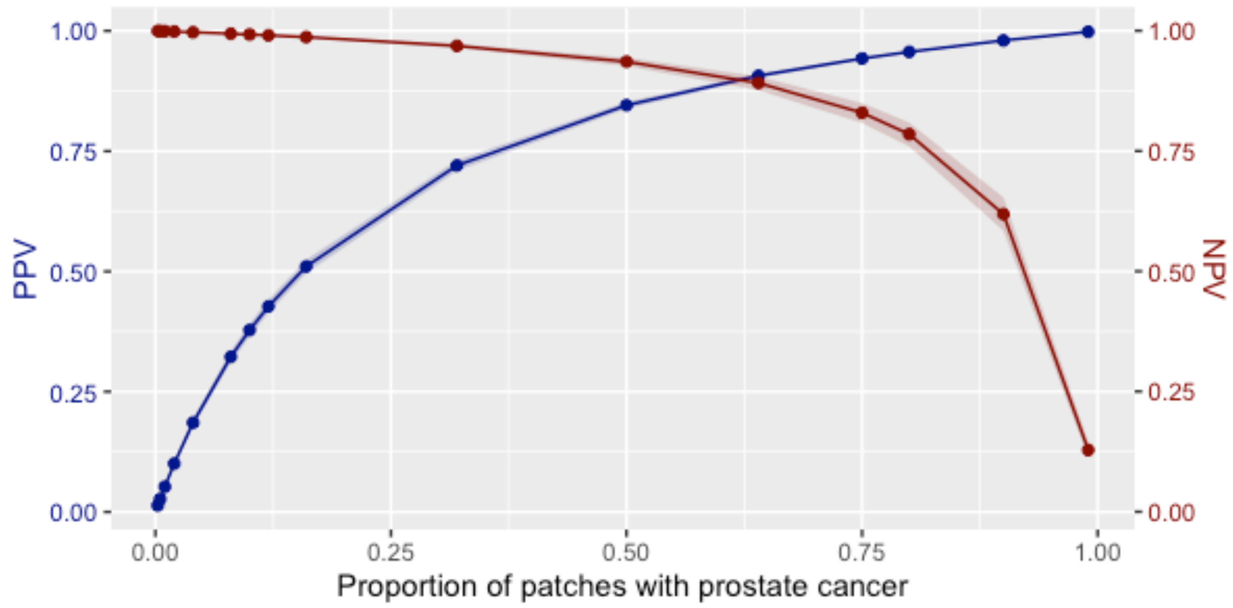

Figure S1: Estimated positive (PPV) and negative predictive values (NPV) for different proportions of patches with prostate cancer. These patches originate from histology images of slides with paled staining due to aging process.

When and when not to use Virtual Pathologist

It is essential to define the intended use cases of the artificial intelligence solution as virtual pathologist (vPatho). vPatho supports only H&E-stained histology images and can function on different versions of staining protocols for H&E. The minimum objective magnification level is 20x for vPatho to run efficiently. Images with 10x objective magnification can function as well but can cause performance degradation for patch-level classification of Gleason pattern 3 or 4, and consequently at the slide or case level. vPatho can be utilized to detect prostate cancers in slides originating from prostate and lymph node tissues, which are the relevant tissue types resulting from curative surgical treatment of prostate cancer. Furthermore, vPatho delivers sustainable performance on

tissue dimensions ranging from laser microdissection to prostatectomy specimen. However, all patches of histology images should meet the reference range for illuminance and Laplacian variance (blurriness measurement); otherwise, vPatho is not guaranteed to deliver robust performance.

vPatho covers two major histological types of prostate cancers (acinar and ductal adenocarcinoma) and indirectly intraductal adenocarcinoma; however, vPatho should be applied with caution in cases with neuroendocrine prostate cancer or other rare types of prostate cancers (e.g., squamous, or small cell) due to the lack of data for these histology subtypes.

vPatho was not assessed on prostatic tissues originating from transurethral resections with cauterization effects (a challenging condition for pathological evaluation) nor on sample tissues originating from cases with castration-resistant prostate cancer. Therefore, it is unknown whether vPatho can function on sample tissues strongly manipulated by cell lysis, endoscopic surgery, or systematic treatment.

vPatho can differentiate PCa from normal tissues, tumor precursor "HGPIN" or prostatitis (GTEX cohort) and accurately automate slide sorting. The most frequent histology morphologies for Gleason patterns are considered by vPatho. Furthermore, vPatho can help in distinguishing intraductal prostate cancer (IDC) from HGPIN with reasonable accuracy. vPatho should be applied to histology images originating from the internet (i.e., microphotograph) or paled stained images with a minimum objective magnification of 20x only after applying algorithms for staining normalization with reference image. The overlapping approach is useful for increasing the chance of more informative content in patches from prostatic tissues and consequently the detection accuracy by vPatho.

Another important benefit of vPatho is its ability to cover situations where Gleason pattern determination is not feasible at the patch level due to existence of other tissue structures between prostate cancer cells (e.g., existence of nerve, inflammatory cell infiltration and medium or large blood vessels).

Gleason pattern 4 seems to significantly impact the discordance level of ISUP grades between artificial intelligence and human readers. The proportion estimation of Gleason pattern 4 is essential in defining different ISUP grades as Gleason pattern 4 characterizes an intermediate histopathological appearance status after Gleason pattern 3 (GP3) and before Gleason pattern 5 (GP5). So, Gleason pattern 4 (GP4) as an intermediate pattern is intuitively more heterogenous and realizing a distinctive boundary definition for GP4 is more complex and challenging than the early (GP3) and advanced cancer patterns (GP5).

We found that mitigating the impact of GP4 on PCa grading is a possible way to improve concordance, per our results. Furthermore, the proportion estimation for different Gleason patterns strongly contributes to the grade discordance (See box plots in **Figure S2**). **Figure S3** provides two example cases to stress the impact of the proportion determination in causing the grade discordance between vPatho and the human observer. The main manuscript has already discussed other factors triggering the grade discordance as well as the accuracy of the computational proportion estimation compared to the current state of art approach.

The evaluation results from vPatho can be integrated into electronic pathology reports with graphical illustrations and the evaluation result can be re-used for other questions (e.g., determining the cancer proximity to the prostate capsule) and to investigate the discordance-associated factors in ISUP grading. The current vPatho version represents an alpha version (i.e., prototype) and is intended only for research

purpose and as assistance tools for pathologists who have the final decision. In the near future, we anticipate having a beta version of vPatho that will integrate the corrections made by the pathologist and will be optimized according to the institutional needs. The future work will examine the acceptance level of the suggestions made by vPatho for the pathologists to draft the final pathology report in a prospective study.

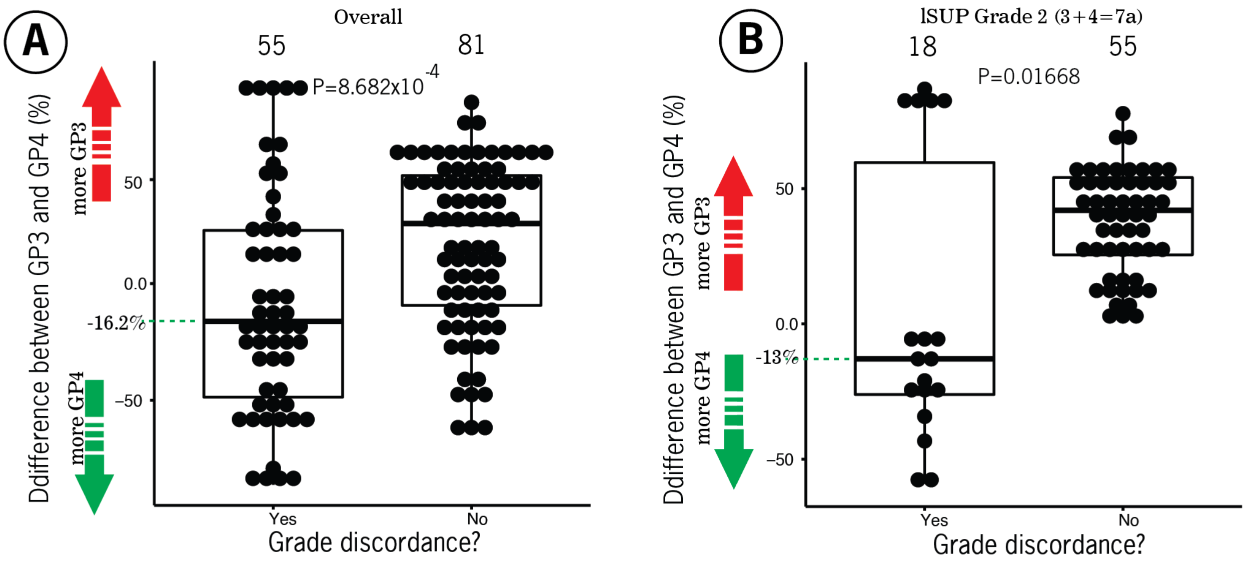

Figure S2: The box plots describe the proportion differences between Gleason patterns 3 and 4 stratified by the grade discordance status in overall cases (A) and in cases with ISUP grade 2 reported in the pathology report (B).

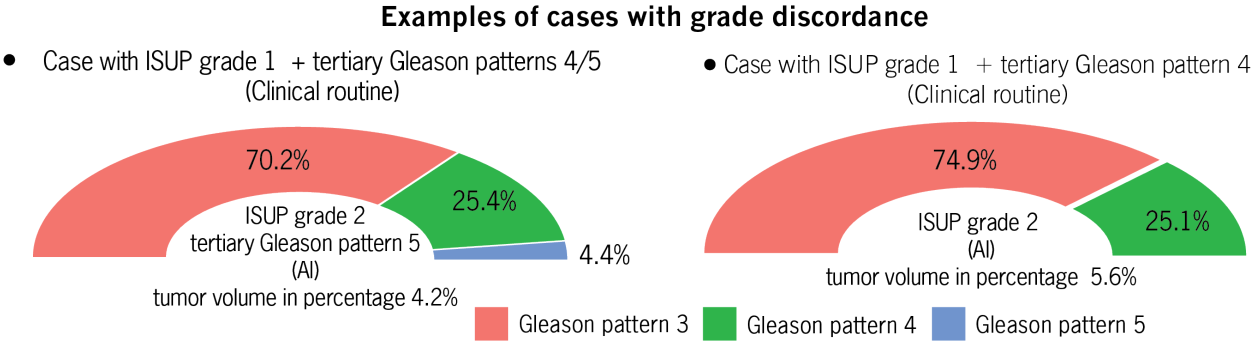

Figure S3: Two example cases with grade discordance between vPatho and the pathology reports. The proportions are estimated by vPatho (AI) and compared the resulting ISUP grade and tertiary Gleason pattern with pathology reports (Clinical routine). The tumor volume in percentage was calculated by vPatho. Despite both vPatho and pathology report determined the right Gleason patterns in these cases, the difference in ISUP grade occurred. Although Gleason pattern 4 detected by vPatho was affected by a higher false negative rate compared to other Gleason patterns 3 and 5, we found that the grade discordance between vPatho and pathology reports was associated with a higher proportion estimation by vPatho for Gleason pattern 4 compared to Gleason pattern 3 (See ***Figure S2****). This finding reveals that the proportion for Gleason pattern marked as tertiary Gleason patterns by pathologists is twice-folder higher than 10% threshold (a corrected threshold for determining the secondary Gleason pattern), indirectly highlighting the inaccurate size estimation (50% of the original size) made by the human observers (pathologists). This result is in accordance with our previous study where we showed that human observers (pathologists) significantly underestimate the size proportion (by 50% of the original tumor) compared to the computer-assisted size estimation on an independent dataset with 255 prostatectomy specimens[1].*

Prostate Cancer

TMA spot sorting according to cancer presence

We evaluated the detection performance of spots with prostate cancer on TMA while considering the correction algorithm, patch overlap and computational load. **Figure S4** summarizes AUROCs after stratifying by the correction algorithm for different patch overlapping rates.

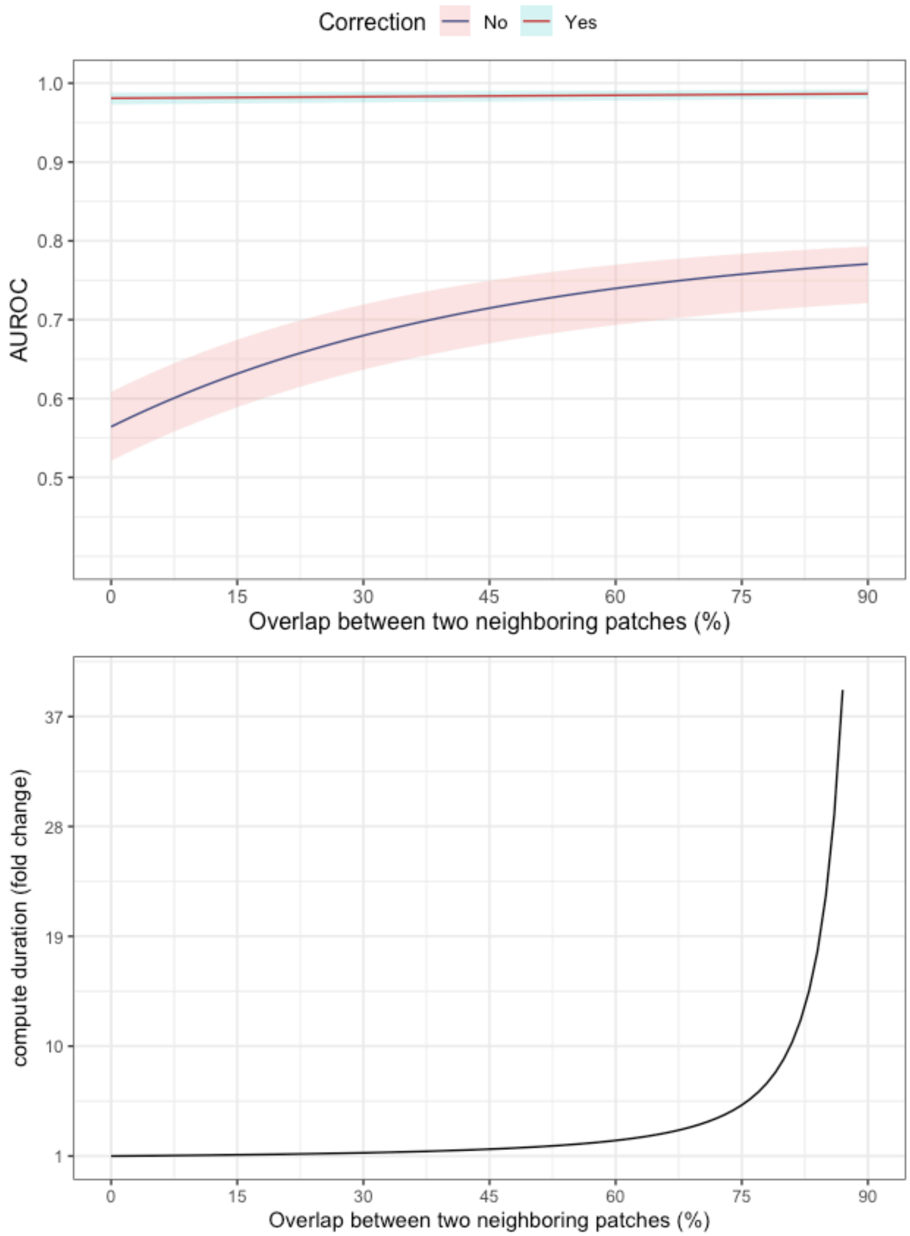

Figure S4: The AUROC performances for prostate cancer detection on held-out TMA spots stratified by the application of the illumination correction algorithm (Correction) for different overlapping rates; The correction algorithms significantly boosted the detection performance (The correction algorithm was developed using only the development set). When the illumination correction algorithm was not applied, a non-linear (asymptotic) association between the overlapping proportions and the detection accuracy was found and an exponential association between the overlapping proportion and the computing workload. However, applying only the overlapping approach provided an inferior detection performance compared to the detection performance with the correction algorithms. AUROC stands for Area Under the Receiver Operating Characteristics. The 95% confidence intervals were calculated using bootstrapping with 100,0000 times spots resampling. The lines were curated and smoothed using asymptotic regression (blue line), linear regression (red line) and exponential algorithm (black line) and four overlapping rates (i.e., 0%, 25%, 75%, 88%). The reference to calculate the fold change for compute duration was the compute duration of the non-overlapping spot detection on 1129 spots at 10x objective magnification (Patch size: ~512 x 512 µm) using a single core (AMD™ Ryzen Threadripper 3970X 32-Core 3.7 GHz Socket Desktop Processor, 128GB and 2 TB PCIe Samsung™ PCIe NVME).

Increasing the overlap rate reduced the false positive rate and was significantly associated with a better sensitivity while the specificity was marginally impacted by this increase as shown in **Figure S5**.

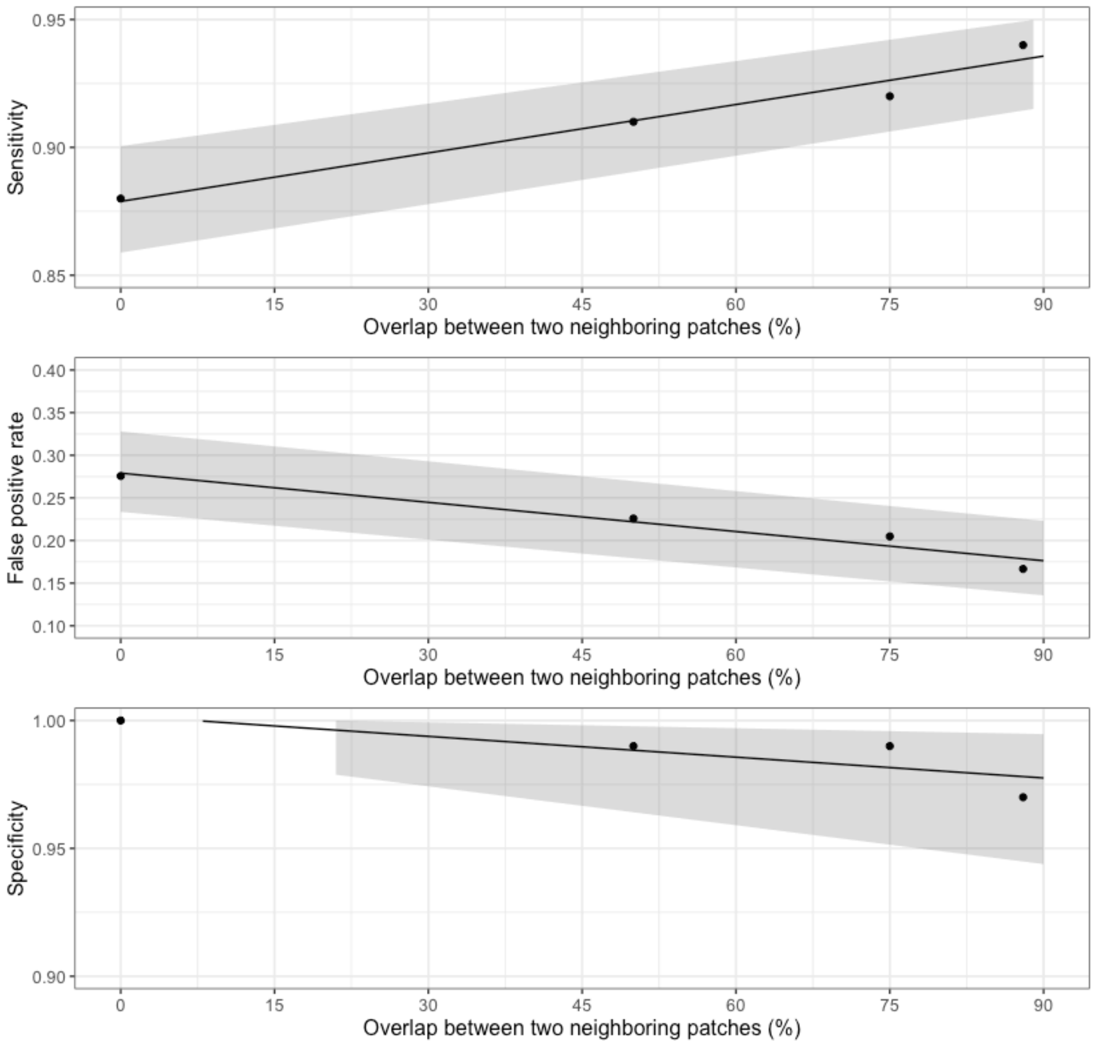

Figure S5 illustrates the associations of sensitivity, false positive rate, or specificity with the overlapping rates of patches with staining color correction. The gray area represents the 95% confidence interval calculated according to Clopper–Pearson.

Table S3 summaries the accuracy performances for the spot screening for prostate cancer on TMA. **Tables S4 to S7** reveal the confusion matrices for detection of spots with prostate cancer for different patch overlap rates**.** Positive (PPV) and negative predictive

values (NPV) are also estimated for different proportions of spots with prostate cancer as shown in **Figure S6.**

Table S3: The detection performance of vPatho for prostate cancer detection on tissue microarray (TMA) spots stratified by patch overlapping rates. CI: Confidence interval.

Patch overlapping rate	Sensitivity, % (95% CI)	Specificity, % (95% CI)	Positive likelihood ratio (95% CI)	Negative likelihood ratio (95% CI)
0%	88.2 (85.8 – 90.2)	100.0 (98.6 – 100)	Infinity	0.12 (0.10 – 0.14)
50%	91.1 (88.9 – 92.9)	98.5 (96.2 – 99.6)	61.0 (23.1 – 161.4)	0.09 (0.07 – 0.11)
75%	92.1 (90.1 – 93.8)	98.5 (96.2 – 99.6)	61.7 (23.3 – 163.3)	0.08 (0.06 – 0.10)
88%	94.0 (92.2 – 95.5)	97.0 (94.2 – 98.7)	31.5 (15.9 – 62.3)	0.06 (0.05 – 0.08)

Table S4: The confusion matrix for prostate cancer detection on tissue microarray (TMA) spots without patch overlapping.

	vPatho		
	Number of TMA spots (%)		
Finding	Positive	Negative	Total
Prostate Cancer	759 (100.0)	102 (27.6)	861 (76.3)
No prostate cancer	0 (0)	268 (72.4)	268 (23.7)
Total	759 (67.2)	370 (32.8)	1129 (100)

Table S5: The confusion matrix for prostate cancer detection on tissue microarray (TMA) spots with 50% patch overlapping.

	vPatho		
	Number of TMA spots (%)		
Finding	Positive	Negative	Total
Prostate Cancer	784 (91.1)	77 (8.9)	861 (36)
No prostate cancer	4 (1.5)	264 (98.5)	268 (64)
Total	788 (69.8)	341 (30.2)	1129 (100)

Table S6: The confusion matrix for prostate cancer detection on tissue microarray (TMA) spots with 75% patch overlapping.

	vPatho		
	Number of TMA spots (%)		
Finding	Positive	Negative	Total
Prostate Cancer	793 (92.1)	68 (7.9)	861 (36)
No prostate cancer	4 (1.5)	264 (98.5)	268(64)
Total	797 (70.6)	332 (29.4)	1129 (100)

Table S7: The confusion matrix for prostate cancer detection on tissue microarray (TMA) spots with 88% patch overlapping.

	vPatho		
	Number of TMA spots (%)		
Finding	Positive	Negative	Total
Prostate Cancer	809 (94.0)	52 (6)	861 (76.3)
No prostate cancer	8 (3.0)	260 (97)	268 (23.7)
Total	817 (72.4)	312 (27.6)	1129 (100)

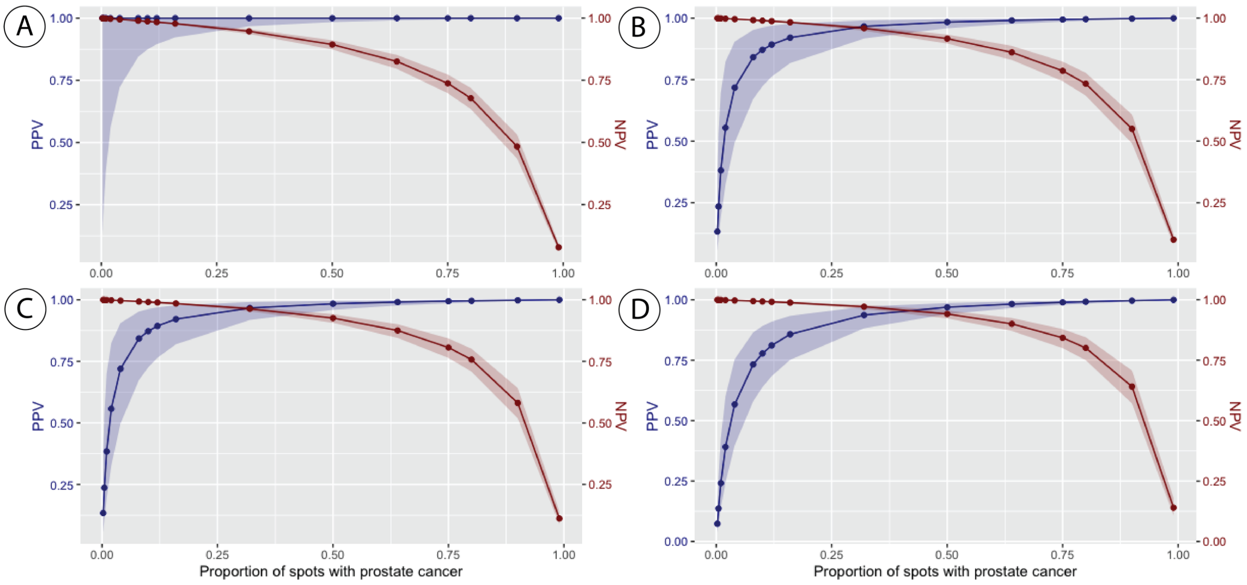

Figure S6. For (A) non-overlapped patch, (B) 50% patch overlapping (C) 75% patch overlapping and (D) 88% patch overlapping, we estimated the positive (PPV) and negative predictive values (NPV) with their 95 % confidence intervals for detection of tissue microarray spots with prostate cancer by vPatho while stratifying by different proportion rates of spots with prostate cancers.

When we normalized the detection performance on core images per TMA, the per TMA AUROC was 0.98 (95% CI: 0.97 – 0.99).

Sorting histology slide images according to cancer presence

The sorting performance on three different datasets are listed in **Table S8**; we identified that slide sorting performance according to the presence status of prostate cancer was in general acceptable on different datasets. Furthermore, the false negative rates were negligibly small, facilitating a better focus on correcting slides falsely flagged

with prostate cancer. vPatho provided demarcation of prostate cancer areas so that the reviewers could trace back the causes for false positivity. We found that the false positivity was associated with some epithelia from seminal vesicle and ductus ejaculatory.

Table S8 Slide sorting performance according to tumor presence on different datasets. TP: True positive; FP: false positive; TN: true negative, FN: false negative.

	vPatho for Slide sorting according to cancer presence				
	Number of slides (%)				
Datasets	TP	FP	FN	TN	Total
Radical prostatectomy	937 (99.0)	9 (1.0)	1 (1.2)	81 (98.8)	1028
PAI-WIST	49 (100)	0 (0)	1 (9.0)	10 (91.0)	60
GTEx Project	5 (100)	0 (0)	0 (0)	35 (100)	40
Total	991 (99.1)	9 (0.9)	2 (1.6)	126 (98.4)	1128

The overall accuracy performance of vPatho for sorting slides according to prostate cancer presence is provided in **Table S9**. The positive (PPV) and negative predictive values (NPV) for different proportions of slide with prostate cancer are illustrated in **Figure S7**. The high NPV and the low negative likelihood ratio reveal that vPatho results for negative slides (i.e., slides with no evidence for prostate cancer) were highly conclusive.

Table S9: the accuracy performance of vPatho for detection of slides with prostate cancer.

Accuracy for detection of slides with prostate cancer by vPatho			
Sensitivity, % (95% CI)	Specificity, % (95% CI)	Positive likelihood ratio (95% CI)	Negative likelihood ratio (95% CI)
99.8 (99.3 – 100)	93.3 (87.7– 96.9)	14.97 (7.96 – 28.14)	0.00 (0.00 – 0.01)

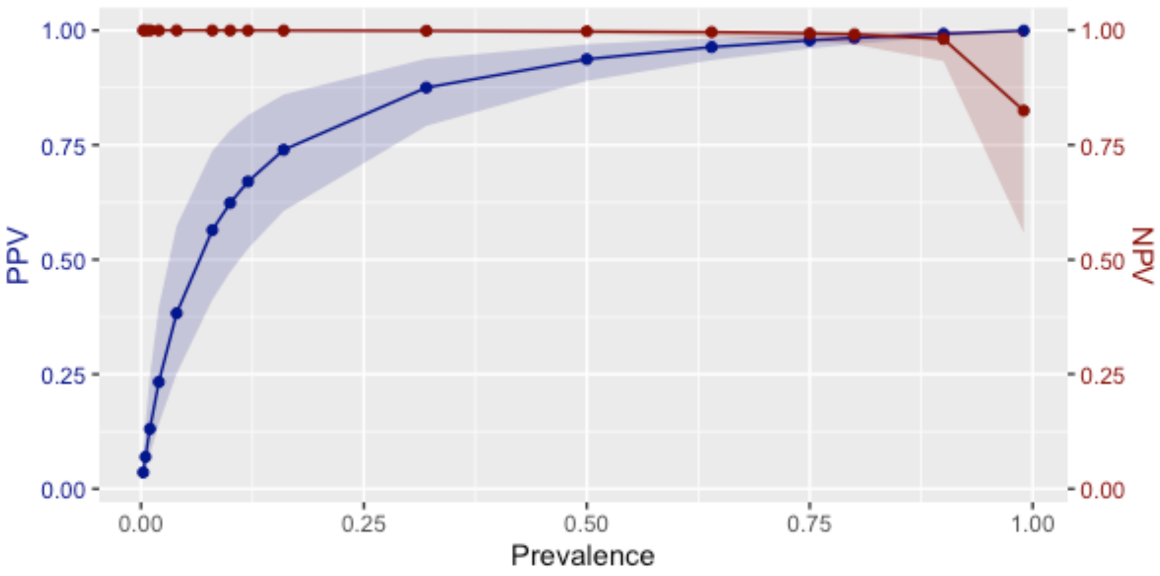

Figure S7: The estimated positive (PPV) and negative predictive values (NPV) with their 95 % confidence intervals for detection of slides with prostate cancer by vPatho stratified by different prevalence rates of slides with prostate cancers.

When we evaluated the sorting accuracy for 136 cases with radical prostatectomy, we identified 7 erroneous cases per 100 cases. The falsely sorted slides were not concentrated to particular cases, indicating the random occurrence of such errors cross the cohort. When the false sorting occurred in cases with 8 whole-mount slides on average, at least one slide of these eight slides was falsely sorted. When we looked at the causes for these errors, we found that one of 7 cases had a false negative slide whereas the remaining 6 cases had at least one false positive slide.

Overall, these findings indicate the necessity of evaluating the detection and diagnosis performance at the case level in addition to the slide level performance as the slide level performance is not reflective of the case level performance. Moreover, we identified that false positive and negative slides are randomly distributed, suggesting that evaluating a single slide per case is not sufficient to generalize the cancer detection performance to the case level. The error occurrence during slide sorting for cancer presence is more impactful at case level than at slide level. The error rate for falsely sorted

slides in affected cases was 14% (range: 11 – 29%), which is significantly higher than the overall error rate for examined slides (1%).

Detection of Lymph node metastases

Table S10 and S**11** reveals vPatho performance in detecting lymph node metastases in 50 lymph nodes. **Figure S8** illustrates the positive and negative predictive values according to different lymph node prevalence rates. Supplementary file 2 illustrates heatmaps for PCa on the lymph nodes.

Table S10: The confusion matrix for the lymph node metastases detection.

	vPatho for Lymph node metastases		
	Number of lymph nodes (%)		
Finding	Positive	Negative	Total
Positive lymph nodes	16 (89)	2 (11)	18 (36)
Negative lymph nodes	1 (3)	31 (97)	32 (64)
Total	17 (34)	33 (66)	50 (100)

Table S11: the detection performance of vPatho for lymph node metastases.

Accuracy for detecting lymph node metastases by vPatho			
Sensitivity, % (95% CI)	Specificity, % (95% CI)	Positive likelihood ratio (95% CI)	Negative likelihood ratio (95% CI)
89 (65 – 99)	97 (84 – 100)	28.44 (4.10–197.15)	0.11 (0.03 – 0.42)

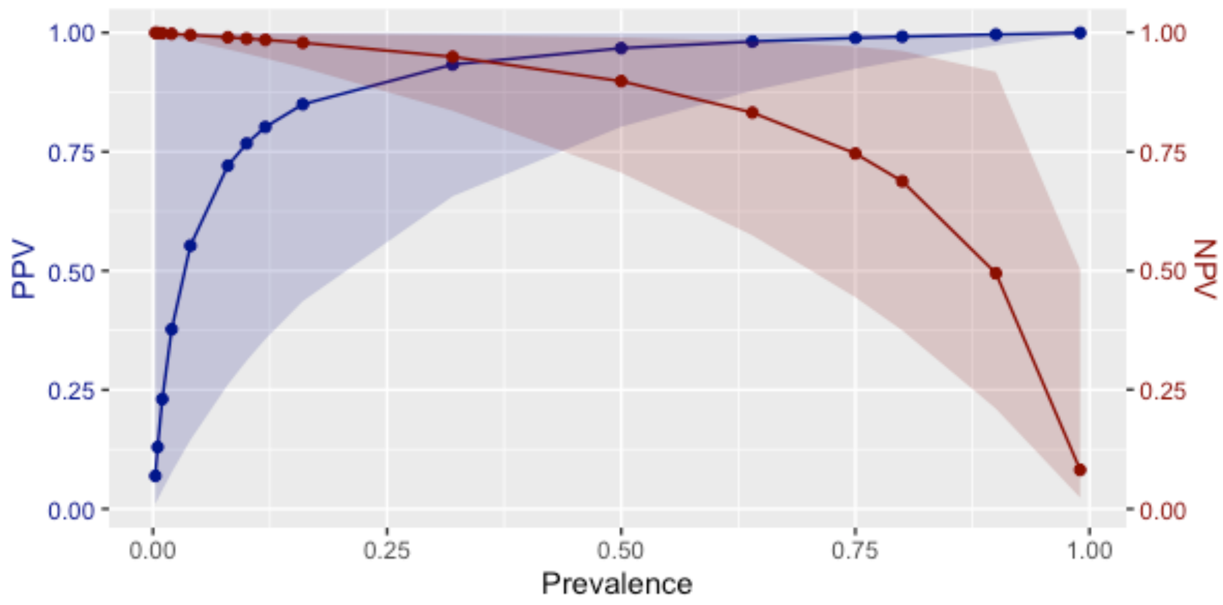

Figure S8: The estimated positive (PPV) and negative predictive values (NPV) for vPatho stratified by different prevalence rates of lymph node metastasis.

Tumor volume estimation

In the test task of tumor volume estimation, the predicted tumor volumes were strongly correlated with the ground truth, with a coefficient of determination (R^2) of 0.987 (95% CI: 0.983–0.991) in 46 cases (368 WM histology images which correspond to 7,360-11,040 biopsy core images) whose histology images had a complete annotation of the cancerous lesions. Furthermore, the paired t-test showed no significant differences between the ground truth tumor volumes and the AI-predicted tumor volumes [t-statistic: -0.499; mean difference: -1.08% (95% CI: -1.44 - 0.72%), P=0.619]. **Figure S9** show the scatter plot with regression line.

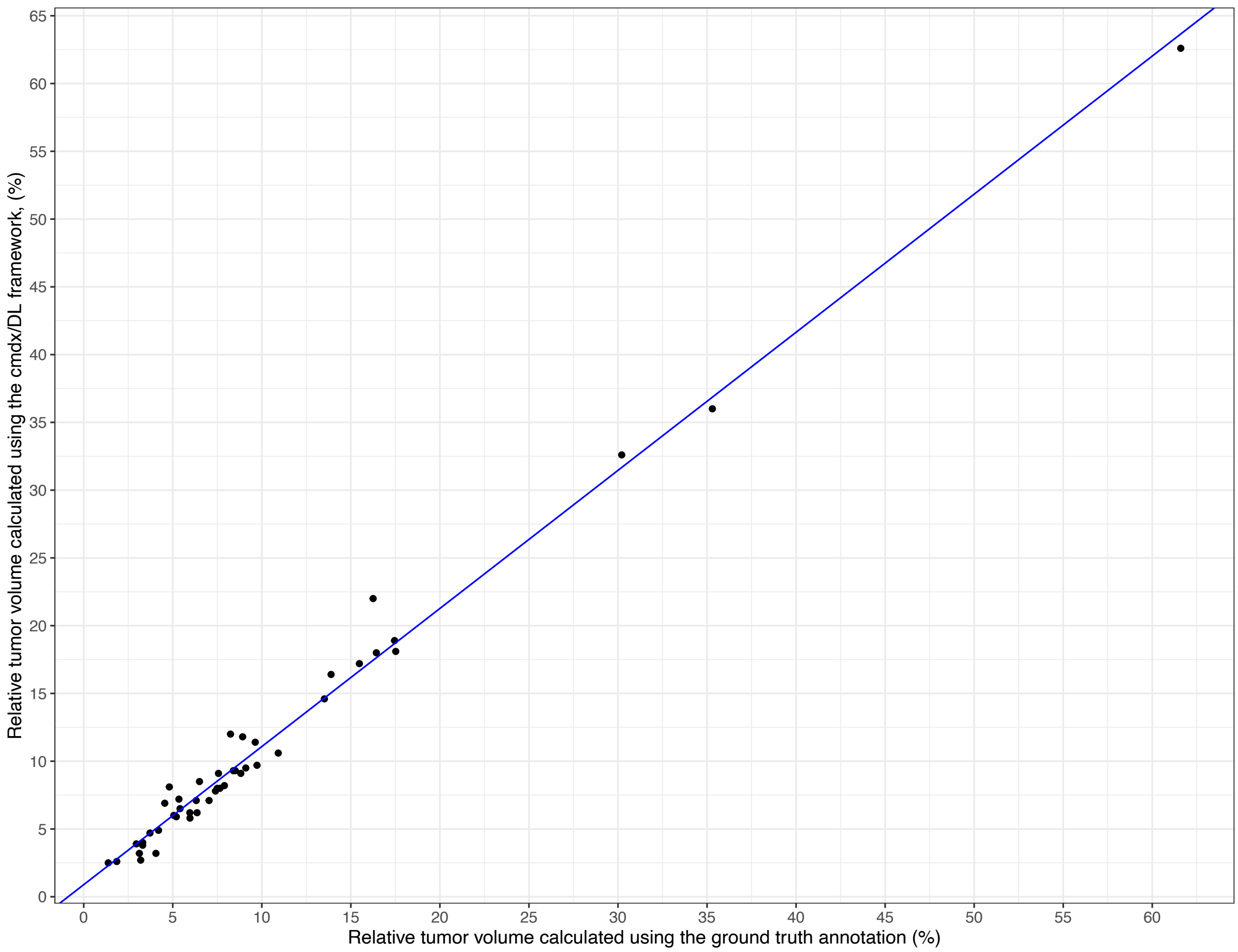

Figure S9: Scatter plot illustrates the high-level correlation of relative tumor volume (i.e., tumor volume in percentage) between the pathologist's annotation and vPatho.

Gleason Patterns and ISUP Grading

Classification performance

When we evaluated the detection of Gleason patterns on images having prostatic tissues in a dimension (*~256 or ~512 μ*m) suitable for laser microdissection, vPatho provided substantial agreement with pathologist annotations for Gleason pattern 3 (Cohen **κ**: 0.785) and 5 (Cohen **κ**: 0.772). In contrast, vPatho had a moderate agreement level with pathologist annotations for Gleason pattern 4 (Cohen **κ**: 0.562). This finding supports our observation that obtaining a substantial agreement for Gleason pattern 4 is

more challenging than other Gleason patterns given its heterogeneity and the broad histological description for this intermediate pattern.

The accuracy of vPatho depended on the magnification level. Here, we found that Gleason pattern 5 was more accurately detectable at 10x objective magnification than at 20x objective magnification. In contrast, the 20x objective magnification level provided a better accuracy for Gleason pattern 3 or 4 compared to 10x objective magnification level, emphasizing the need of deeper and fine-granular content information for a better classification performance for Gleason pattern 3 and 4. When we explored the activation maps for Gleason pattern 3 and 4, we identified that the stroma space or the space between glandular epithelial are contributing to the model decision. In contrast, Gleason pattern 5 is generally defined based on more global glandular structure disappearance as well as on the necrosis occurrence. Overall, these observations are in alignment with the clinical knowledge that the stromal space between glandular epithelia and the global glandular structure appearance are major decision factors in differentiating between Gleason patterns. The contingency tables or confusion matrices for Gleason patterns stratified by the magnification level are provided in **Tables S12 - S17**. We stratified Gleason pattern 4 into different morphologies to cover the heterogeneity conditions of Gleason pattern 4. Moreover, we incorporated HGPIN (tumor precursor) for the differential detection task.

Table S12: Confusion matrix for Gleason pattern 3 detection by vPatho on patch images at 10x objective magnification. The dimension of a patch corresponded to 512 x 512 µm.

	vPatho for Gleason pattern 3		
	Number of patch images (%) – 10x objective magnification		
Finding	Positive	Negative	Total
Gleason pattern 3	253 (81.4)	58 (18.6)	311 (27.6)
HGPIN, Gleason pattern 4 or 5	108 (13.2)	709 (86.8)	817 (72.4)
Total	361 (32.0)	767 (68.0)	1128 (100)

Table S13: Confusion matrix for Gleason pattern 3 detection by vPatho on patch images at 20x objective magnification. The dimension of a patch corresponded to 256 x 256 µm.

	vPatho for Gleason pattern 3		
	Number of patch images (%) – 20x objective magnification		
Finding	Positive	Negative	Total
Gleason pattern 3	1024 (84.6)	187 (15.4)	1211 (31.5)
HGPIN, Gleason pattern 4 or 5	167 (6.4)	2462 (93.6)	2629 (68.5)
Total	1191 (31.0)	2648 (69.0)	3840 (100)

Table S14: Confusion matrix for Gleason pattern 4 detection by vPatho on patch images at 10x objective magnification. FN: False negative; TN: true negative; FP: false positive; TP: true positive.

	vPatho for Gleason pattern 4			
	Number of patch images (%) – 10x objective magnification			
Finding	FN	TN	FP	TP
High-grade Prostatic intraepithelial neoplasia		250 (100.0)	0 (0.0)	
Gleason pattern 3		296 (95.2)	15 (4.8)	
Gleason pattern 4	264 (58.4)			190 (41.6)
Cribriform glands	27 (75.0)			9 (25.0)
Poorly formed glands	130 (61.9)			80 (38.1)
Fused glands	14 (73.7)			5 (26.3)
Poorly formed and fused glands	93 (50.0)			96 (50.0)
Gleason pattern 5 (Mixed)		82 (73.2)	31 (27.8)	
Total	534 (46.0)	628 (54.0)	46 (10.8)	380 (89.2)

Table S15: Confusion matrix for Gleason pattern 4 detection by vPatho on patch images at 20x objective magnification. FN: false negative; TN: true negative; FP: false positive; TP: true positive.

	vPatho for Gleason pattern 4			
	Number of patch images (%) – 20x objective magnification			
Finding	FN	TN	FP	TP
High-grade Prostatic intraepithelial neoplasia		775 (91.7)	70 (8.3)	
Gleason pattern 3		1043 (86.1)	168 (13.9)	
Gleason pattern 4	326 (23.7)			1048 (76.3)
Cribriform glands	40 (34.2)			77 (65.8)
Poorly formed glands	69 (12.8)			469 (87.2)
Fused glands	6 (11.5)			51 (89.5)
Poorly formed and fused glands	211 (31.9)			451 (68.1)
Gleason pattern 5 (Mixed)**		245 (59.8)	165 (40.2)	
Total	652 (24.0)	2063 (76.0)	403 (16.1)	2096 (83.9)

Table S16: Confusion matrix for Gleason pattern 5 detection by vPatho on patch images at 10x objective magnification.

	vPatho for Gleason pattern 5		
	Number of patch images (%) – 10x objective magnification		
Finding	Positive	Negative	Total
Gleason pattern 5	91 (81.4)	21 (18.8)	112 (9.9)
HGPIN, Gleason pattern 3 or 4	30 (3.0)	986 (97.0)	1016 (90.1)
Total	121 (10.7)	1007 (89.3)	1128 (100)

Table S17: *Confusion matrix for Gleason pattern 5 detection by vPatho on patch images at 20x objective magnification.*

	vPatho for Gleason pattern 5		
	Number of patch images (%) – 20x objective magnification		
Finding	Positive	Negative	Total
Gleason pattern 3	220 (53.3)	193 (46.7)	413 (31.5)
HGPIN, Gleason pattern 4 or 5	173 (5.0)	3254 (95.0)	3427 (68.5)
Total	393 (10.2)	3447 (89.8)	3840 (100)

Table S18 provides the detection accuracy for Gleason pattern 3 (GP3), 4 (GP4) and 5 (GP5). **Figure S10** illustrates the positive predictive values (PPV) and negative predictive values (NPV) for GP3, GP4 and GP5. We adjusted the optimal prevalence rate for GP3 and GP4 to GP5 by considering the patch dimension for a better comparison (a patch of GP5 ≈ 4 patches of GP3 or GP4). A minimum prevalence rate of 7.8% (95% CI: 7.5% – 8.25%) for GP3 was required to achieve PPV of at least 0.85 (95 CI%: 0.82 – 0.87); the minimum required prevalence rate was 13.3% (12.5% – 13.5%) for GP4 to achieve PPV ≥ 0.84 (95 CI%: 0.82 – 0.85), or 10% (8% – 12%) for GP5 to realize a PPV ≥0.75 (95 CI%: 0.67 – 0.83) on 1000 patches.

Table S18: The detection accuracy of vPatho on detecting Gleason pattern 3, 4, and 5.

Gleason Pattern	Sensitivity, % (95 CI)	Specificity, % (95 CI)	Positive likelihood ratio (95 CI)	Negative likelihood ratio (95 CI)
3	85.6 (83.9 – 87.9)	92.9 (91.9 – 93.4)	12.18 (10.59 – 14.01)	0.15 (0.13 – 0.17)
4	76.3 (74.6 – 0.78)	83.7 (82.1 – 85.1)	4.67 (4.26 – 5.12)	0.28 (0.26 – 0.30)
5	81.3 (72.8 – 88.0)	97.1 (95.8 – 98.0)	27.52 (19.13 –39.58)	0.19 (0.13 – 0.28)

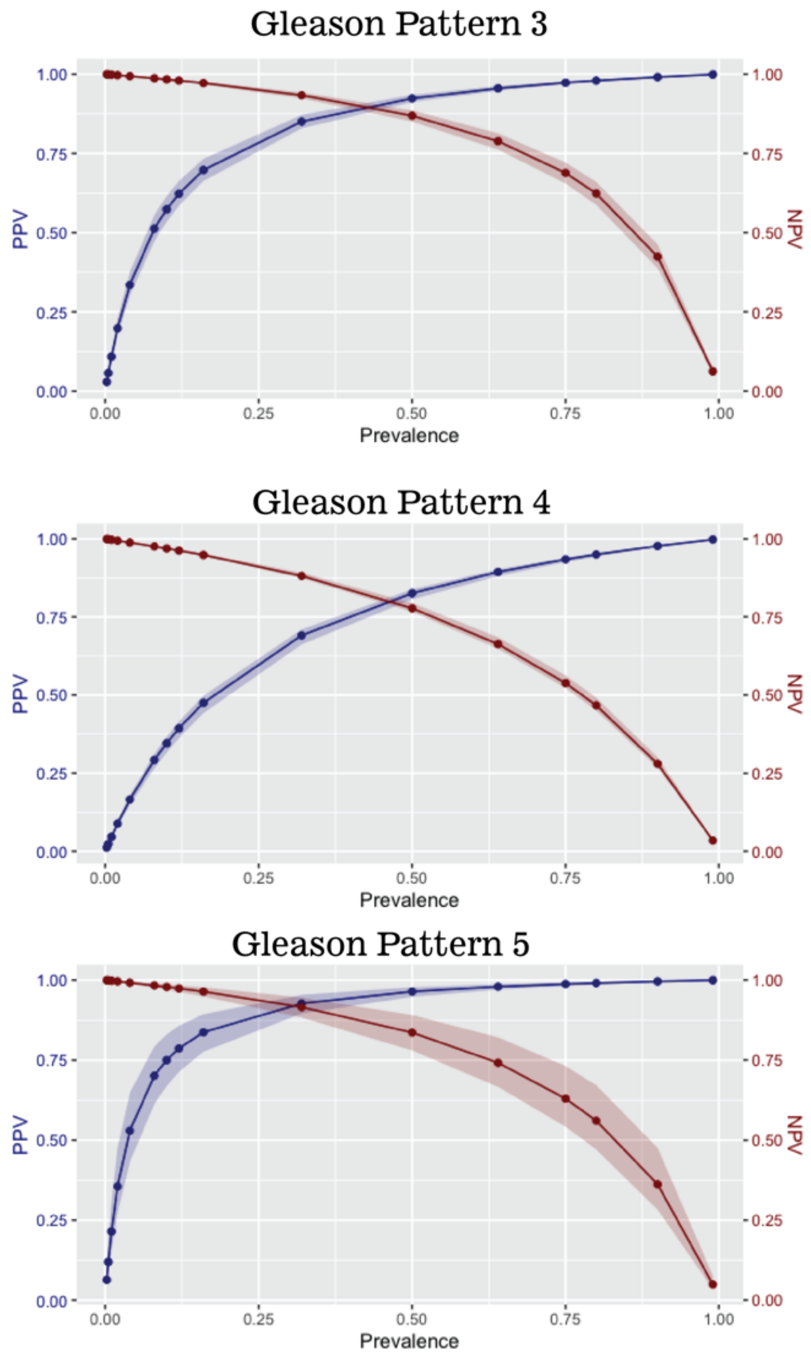

Figure S10: Estimated Positive (PPV) and negative predictive values (NPV) for different prevalence rates of Gleason pattern 3 (GP3), 4 (GP4) and 5 (GP5) on 10,000-times patch resampling with prostate cancer that have a tissue dimension of 512x512 µm for GP5 and 256x256 µm for GP3 and 4.

The error distribution cross Gleason patterns

The unequal distribution in error rates between Gleason patterns can impact the concordance levels for ISUP grading between vPatho and human observers. Therefore, we examined the false positive rates (FPR) and false negative rates (FNR) and the ratio of FPR/FNR for each Gleason pattern determined by vPatho at patch level. We found that the false positive rates were comparable between Gleason patterns with a maximum deviation of 3% (i.e., FPR for GP3: 15%; GP4: 16% and GP5: 18%); while the false negative rates and ratios of FPR/FNR for GP3 (5%) and GP5 (6%) were comparable, the false negative rate for GP4 was clearly higher (24%) and consequently the ratio of FPR/FNR was lower than those of GP3 and GP5.

Overall, these data conclude that the false positive rates for classifying all Gleason patterns are approximately similar, suggesting a negligible impact of false positive rates on estimating the proportion of Gleason patterns by vPatho. However, special attention is required for the FNR of GP4 if the overall grade discordance between vPatho and a pool of six pathologists is due to the lower proportion of GP4 compared to that of GP3 or GP5.

ISUP grading for radical prostatectomy

Since ISUP grading for radical prostatectomy depends on the proportions of Gleason patterns, we measured the correlation between the percentages of Gleason patterns estimated by vPatho and the ISUP grades defined by clinical pathologists. We found that the percentage of GP3 was significantly and inversely correlated with ISUP grades of the pathology reports (τ: -0.50; $P<0.0001$), whereas the percentages of GP4 (τ: 0.44; $P<0.0001$) and GP5 (τ: 0.43; $P<0.0001$) were significantly and positively correlated with ISUP grades of the pathology reports (**Figure S11**). This finding reveals a clinically

reasonable correlation between the results of vPatho for the pattern proportion estimation and the ISUP grade determined by clinical pathologists.

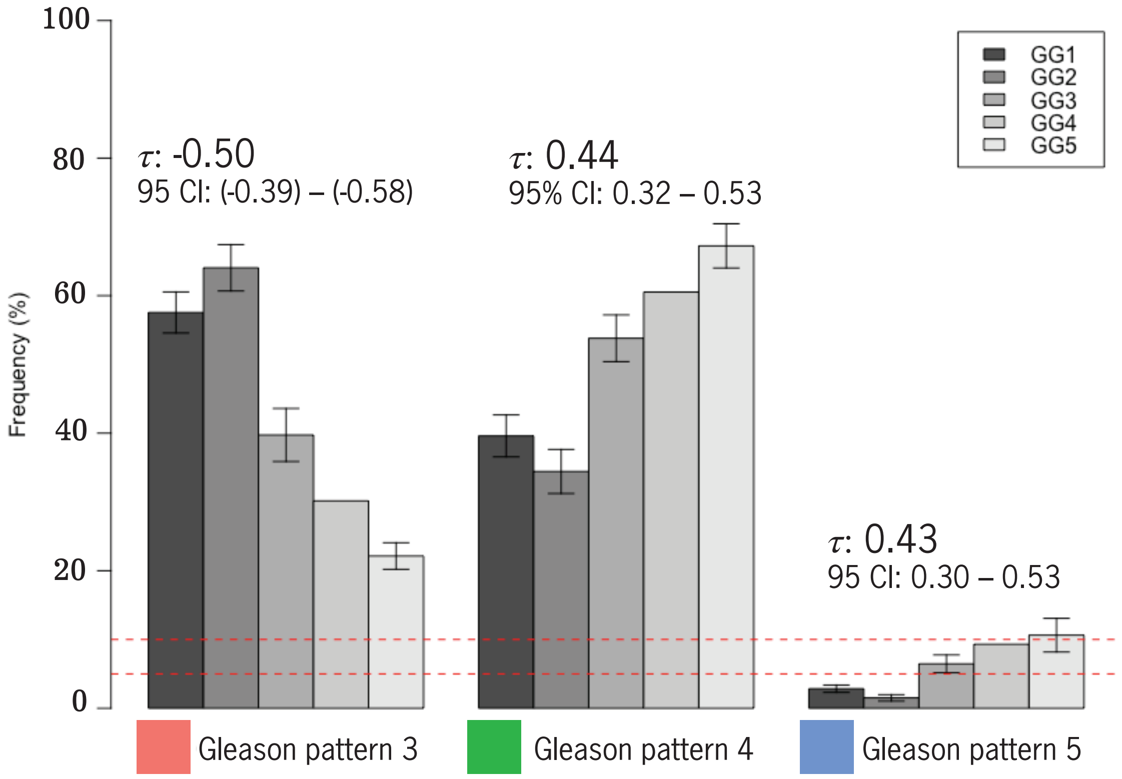

Figure S11: The frequencies of Gleason patterns (GP) after stratifying by the ISUP Grades (GG1-5). The correlation was measured using Kendall tau (τ) and the 95% confidence interval was determined using bias corrected accelerated bootstrap with 1,000 resampling. The correlation between GPs and ISUP grades is significant, and the frequencies of GP are in alignment with ISUP grades. The red lines reveal the thresholds (5% and 10%) to determine the secondary Gleason pattern for the ISUP grade on radical prostatectomy. The 5% threshold is suggested by ISUP to determine the secondary and tertiary Gleason patterns, whereas the 10% threshold is a correction of ISUP threshold based on results of our previous study[1] on an independent cohort that has increased the concordance rate between virtual pathologist and pathology reports by 20% (quadratic weighted κ*: 0.44 to 0.64).*

The threshold definition for secondary and tertiary Gleason pattern has also significantly impacted the concordance level between vPatho and a pool of pathologists that we already discussed in the main manuscript (**Figure S8**).

When we compared the percentage deviation between GP3 and GP4 after the threshold correction, the null hypothesis was that the proportion of GP4 is lower in discordance group given that vPatho had a higher false negative rate for GP4 compared to GP3 and GP5, consequently associated for discordance. Interestingly, we identified that cases with grade discordance between vPatho and a pool of pathologists had a higher GP4 percentage than cases with grade concordance, thereby rejecting the null hypothesis. This interesting finding reveals that vPatho is not the only reason for discordance and raises a serious concern about the inaccurate proportion estimation made by human observers in the current clinical practice that clearly rescinds the impact of the false negative rate of vPatho for GP4.

Factors associated with disagreement in ISUP Grading

We designed four mixed-effects logistic regression models to identify the associations of variables likely impacting the grade discordance status for radical prostatectomy. The variables are listed in **Table S19** for each model.

Table S19 summarizes the variables considered in four different models to explore for factors impacting the grade discordance status.

	Variables	
Model (Aim)	Fixed effects	Random effect (random intercept)
**A.**<br>(To evaluate the indicative association of the ISUP grading made by the pathologists on the grade disagreement regardless of the pathologist who provided the tumor grading)	-Tumor volume in percentage<br>-Year of the pathology report (tumor grading)<br>-Proportion of positive slides<br>-The total number of slides<br>-Gleason grade made by the pathologists	Pathologists
**B.**<br>(To evaluate the indicative association of the pathologist on the grade disagreement regardless of the ISUP grade made by the pathologists)	-Tumor volume in percentage<br>-Year of the pathology report (tumor grading)<br>-Proportion of positive slides<br>-The total number of slides<br>-Pathologists	Gleason grade made by the pathologists
**C.** (To evaluate the indicative association of the pathologist on the grade disagreement regardless of the ISUP grade made by the pathologists)	-Tumor volume in percentage<br>-Year of the pathology report (tumor grading)<br>-Proportion of positive slides<br>-Gleason grade made by AI	Pathologists
**D.** (To evaluate the indicative association of variables that are determined by AI on the grade disagreement regardless of the pathologist who conducted the tumor grading that we compared with)	-Tumor stage (pT)<br>-locoregional lymph node metastases status (pN)<br>-Surgical margin status (R) or capsule proximity of PCa<br>-Gleason grade made by the pathologists/by AI<br>-Tumor volume in percentage<br>-Proportion of positive slides	Pathologists

Figure S12 summarizes the odd ratios for grade discordance. The following subsections will provide and discuss the results from each mixed effect model.

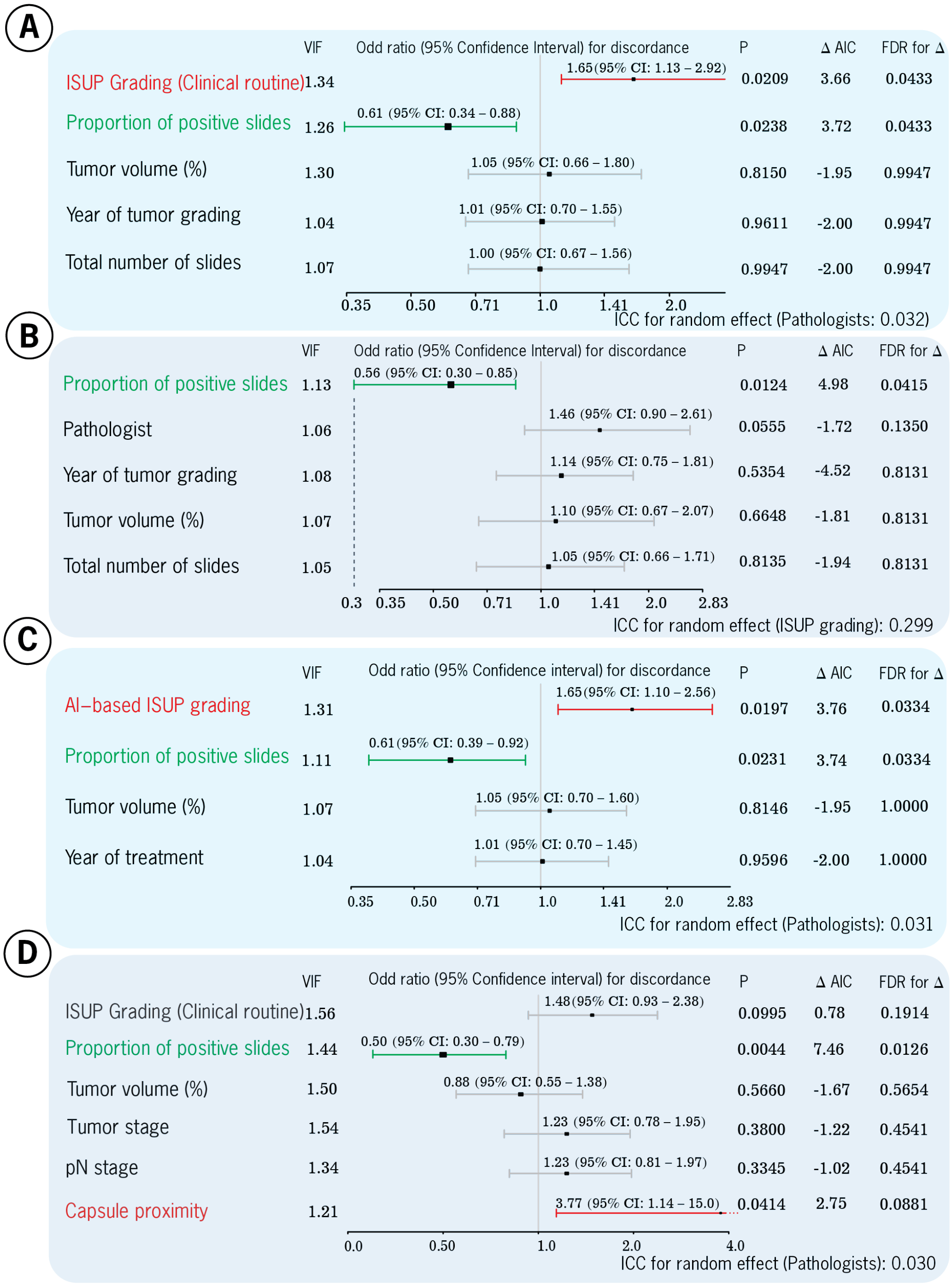

Figure S12 summarizes the results from four mixed-effects logistic regression models to determine factors associated with the grade discordance. Year of treatment is equal to the year of grading. AIC: Akaike information criterion is used to measure the goodness of fit; ΔAIC: the change in AIC after excluding the corresponding variable (Higher is better); FDR: false discovery rate; The statistical significance was defined when FDR is <0.1 and P<0.05; pN stage: locoregional lymph node metastasis; ICC: Intraclass correlation coefficient that measures the random effect on the odd ratio estimation. The random effect of pathologists (individuum) was negligible minimal while the random effect of ISUP grading made during clinical routine was weak. VIF: Variance inflation factor was applied to diagnose collinearity, VIF below 2.0 suggests that collinearity is negligible low.

To evaluate the indicative association of the ISUP grading made by the pathologists on the grade disagreement regardless of the pathologist who provided the tumor grading.

In this multivariable model, pathologist grading was associated with grade discordance (Odd ratio -OR-: 1.65; 95% CI: 1.13 – 2.92; P=0.0209) and its exclusion from the mixed model significantly increased AIC (FDR: 0.0433), validating the associative relevance of pathologists grading for the grade discordance. Although the slide number per case was not indicative nor significant, the proportion of positive slides per case was, however, indicative, and significant for the grade discordance status (OR: 0.61; 95% CI: 0.34 – 0.88; P=0.0238; ΔAIC= 3.72 by FDR=0.0433). The year of tumor grading as well as the tumor volume in percentage (TuVol%) were not significant indicator for grade discordance status (**Figure S12A**).

To evaluate the indicative association of the pathologist on the grade disagreement regardless of the ISUP grade made by the pathologists

Regardless of the ISUP grade, the pathologist as individuum who evaluated the case during the clinical routine was not indicative for the grade disagreement with vPatho (OR: 1.46; 95% CI: 0.90–2.61; P=0.0555). The proportion of positive slides remained a significant indicator for the grade discordance status where the proportion increase for the positive slides was associated with a better grade concordance as seen in the previous model. The year of tumor grading, the total number of slides per case and TuVol% were not significantly indicative for grade discordance (**Figure S12B**).

To evaluate the indicative association of variables that are determined by AI on the grade disagreement regardless of the pathologist who conducted the tumor grading that we compared with.

vPatho grading was also significantly associated with the discordance status in addition to the proportion of positive slides; parallelly, the indicative values for year of tumor grading and TuVol% remained unchanged (**Figure S12C**).

To evaluate the indicative association of significant variables on disagreement while considering the tumor stage and the status for locoregional lymph node metastasis status of prostate cancer.

We found that ISUP grading (regardless of whether it was made by pathologists or vPatho) was no longer a significant indicator for the grade discordance status when we considered pT and pN as well as the proximity of PCa to the prostate capsule. Interestingly, we found the PCa proximity to the prostate capsule is a significant indicator for the discordance status (OR: 3.77; 95% CI: 1.14 – 15.04; P= 0.04135) in addition to the proportion of positive slides (OR: 0.50; 95% CI: 0.30 – 0.79; P=0.00441) as shown in **Figure S12D**. When we replaced the PCa proximity with the surgical margin status determined by the pathologist, we identified that a positive surgical margin was not significantly indicative for the grade discordance (OR: 1.952; 95% CI: 0.849 – 4.55; P=0.10). Another interesting observation is the absence of the correlation between the surgical margin status and the capsule proximity of PCa (Kendall tau correlation coefficient: 0.05; P=0.5884), revealing that the definition of PCa nearness to the prostatic capsule (not to be confused with capsule infiltration) is not associated with the positive surgical margin. To measure the association between the positive surgical margin and grade discordance in cases with positive capsule proximity of PCa, correlation and dependency analyses were conducted. As post-hoc analysis for cases with positive PCa proximity to the prostatic capsule, a Kendall tau correlation coefficient of 0.206 reveals a weak but a significant concordance between grade discordance and the positive surgical margin (P=0.0269); the Fisher's exact test (1,000 replicates used in the Monte Carlo simulation test) identified a significant dependency between grade discordance and positive surgical margin (OR:

2.44; 95% CI: 1.023 – 5.97, P=0.0419). **Table S20** provides the 2x2 contingency table on cases with positive capsule proximity of PCa.

Table S20 shows a contingency table between surgical margin status and grade agreement between virtual pathologist and a pool of 6 pathologists in cases with positive capsule proximity of PCa (116 of 136 cases). The rate of positive surgical margin is in alignment with its incidence rate reported in large datasets.

**Grade agreement**	**Negative surgical margin**	**Positive surgical margin**	**Total, n (%)**
**Concordance, n (%)**	51 (63.8)	15 (41.7)	66 (56.9)
**Discordance, n (%)**	29 (36.2)	21 (58.3)	50 (43.1)
**Total, n (%)**	80 (69.0)	36 (31.0)	116 (100)

Mediation analyses

To determine the next associated (indirect) factors to pathologist as individuum or the positive slide proportion (percentage of slides with prostate cancer) identified by the previous section, we utilized the median analyses. In multivariate analyses, we found that the year of grading was the next predictor with the lowest adjusted P-value and associated with pathologist as individuum, reflecting a real practice in clinical routine where different pathologists rotate for prostatectomy evaluation. Moreover, the tumor volume in percentage was positively associated with the positive slide proportion for prostate cancer, revealing the intuitive association of the tumor extent with the vertical tumor spread. Overall, these findings support our notion that the study cohort is representative for clinical routine and tumor extent. **Figure S13** provides a summary of the analyses.

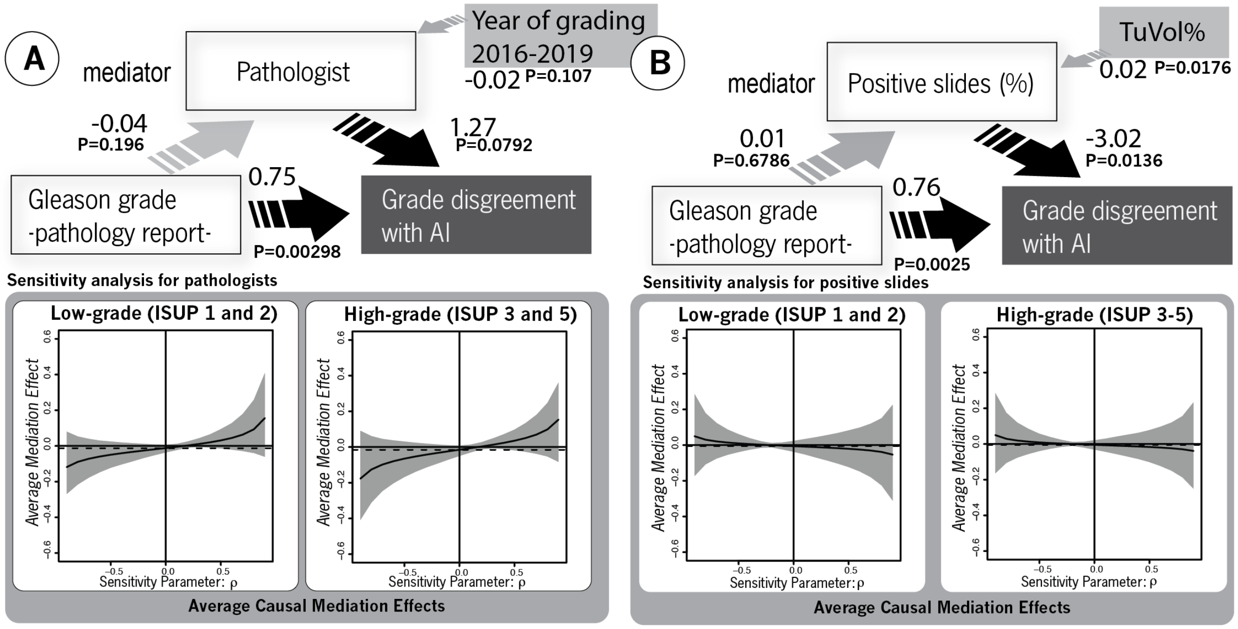

Figure S13: Median analyses was conducted to determine the direct and indirect associations. For pathologist as mediator (A), the ISUP grading is not significantly predictive for the pathologist who conducted the grading; the year of tumor grading was the first predictor for the pathologist with the lowest adjusted P-value. The sensitivity analysis further reveals that the effect of pathologist as individuum on the grade discordance does exist but of limited significance and robustness (influenced by other factors as the course of the continuous black line in the plot for sensitivity analyses reveals) and not altered by the grade levels (low-grade vs. high-grade). As conclusion, the tumor grade made by pathologists (not at individuum level) is significantly associated with grade discordance (disagreement). (B) For the positive slide proportion (i.e., proportion of slides with prostate cancer), ISUP grade was not predictive for the positive slide proportion; the tumor volume in percentage (TuVol%) was significantly associative with the positive slide proportion. The sensitivity analysis reveals the effect robustness of positive slide proportion and its independency of ISUP grade levels (low-grade vs. high-grade). Overall, the positive slide proportion remained to be indicative for the grade discordance status and TuVol% is associated with the positive slide proportion. The average direct and indirect (median) effect estimates, and corresponding adjusted P values are provided. P<0.05 was considered as significant to achieve Power of 85% (Power was estimated based on Monte Carlo Simulation on 1,000 resampling).

Detection of cancer morphology

Cancer morphology can be a sign for aggressive PCa. We thus evaluated vPatho for detecting cribriform pattern and ductal morphology, both of which are known for being prognostic parameters for advanced prostate cancer[2,3]. Here, the test condition simulated a research environment, where the lesions of interests were fixed prior, to identify regions (512x512 μm) with the desired cancer morphology (i.e., cribriform, and ductal morphology) suitable for laser microdissection. Such test conditions also reflect human review of regions of interests. We found that the agreement levels for cribriform pattern (Cohen $\boldsymbol{\kappa}$ = 0.710; 95% CI: 0.641 – 0.772) and ductal morphology (Cohen $\boldsymbol{\kappa}$ = 0.771;

95% CI: 0.751 – 0.797) were substantial, emphasizing a reasonable agreement level between vPatho and the pathologist. **Table S21** provides the detection accuracy for cribriform pattern and ductal morphology.

Table S21: The contingency table for cribriform pattern and ductal morphology. TN: true negative, FP: false positive, TP: true positive. NPV: Negative predictive value.

Test	vPatho			
	Number of patch images (%) – 10x objective magnification			
Finding	FN	TN	FP	TP
Cribriform pattern	24 (2.9)	812 (97.1)	24 (26.0)	68 (74.0)
Ductal morphology	136 (15.0)	769 (85.0)	96 (8.0)	1111 (92.0)

The specificity and sensitivity for cribriform pattern were 97.1% (95% CI: 95.8 – 98.1) and 73.9% (95% CI: 63.7 – 82.5) on patches with prostatic sample dimension of 512 × 512 μm at 10x magnification. For ductal morphology, the sensitivity and the specificity were equal (89%; 95% CI: 87 – 91%). The positive likelihood ratio was 25.75 (95% CI: 17.04 – 38.89) for cribriform pattern and 8.03 (95 % CI: 6.64 – 9.70) for ductal morphology, whereas the negative likelihood ratio was 0.27 (0.19, 0.38) for cribriform pattern and 0.12 (95% CI: 0.10, 0.14) for ductal morphology.

Given that the prevalence of cribriform pattern or ductal morphology within the prostate cancer sample dimension of 512 × 512 μm at 10x magnification is unknown in the population, we assumed different prevalence rates to determine the positive (PPV) and negative predictive values (NPV) shown in **Figure S14**. We found that prevalence rates lower than 10% had the lowest PPVs and the highest NPV. Our results indicate that cribriform pattern was well detected when the prevalence rates were between 4% and 32%. In contrast, our results also show that the detection of ductal adenocarcinoma is challenging and requires further improvement, given that ductal adenocarcinoma is a

rare type of prostate cancer. Its frequency in radical prostatectomy specimens is estimated to be between 0.08% and 13%[3,4]. Having a high NPV for ductal adenocarcinoma or cribriform patterns in very low prevalence rates (<5%) can be beneficial to exclude trivial tissue areas with high certainty and consequently optimize the screening procedure made by the pathologists (i.e., reducing the tissue area to screen).

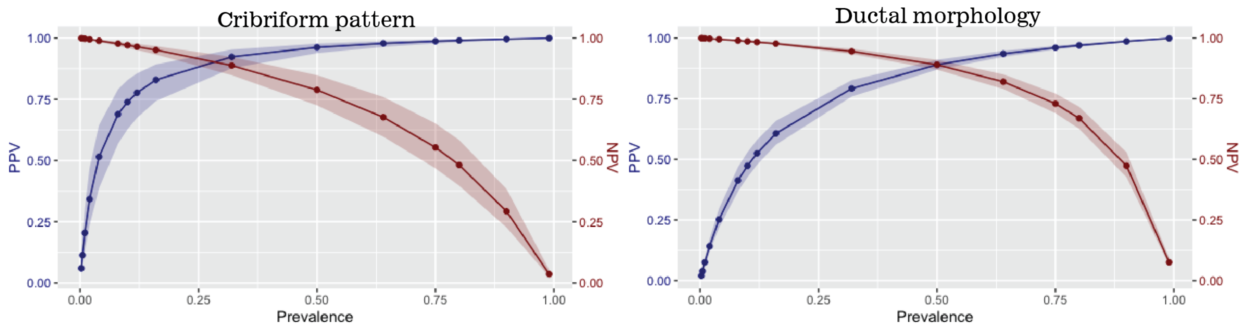

Figure S14: Estimated positive (PPV) and negative predictive values (NPV) with 95% confidence intervals for different prevalence rates of the presence of cribriform pattern and ductal morphologies in 1000 patches. A single patch represents a prostatic tissue with prostate cancer and a dimension of 512×512 μm at 10x objective magnification.

Detection of mesenchymal tissues

Examining mesenchymal tissues like nerve, vessels and inflammatory cell infiltration are an integral part of the pathology evaluation. Therefore, we examined vPatho to detect these tissues by defining a test condition that screens for these tissues in regions within the prostate randomly defined by pathologist. Tissue dimension (512 μm) suitable for microdissection was considered. Inflammatory cell infiltration was accurately detected in 90.8% of the patches, thereby achieving AUROC of 0.951 (95% CI: 0.937 – 0.963) and Cohen **κ** of 0.81 (95% CI: 0.776 – 0.850, almost perfect agreement). The detection precision of nerve structure was high, but with moderate agreement level, denoting a sufficient accuracy for screening purposes where a high precision is desired. In contrast, the vessel detection was a challenging task due to the similarity between

tunica media of the large vessels and the smooth muscle and connective tissues surrendering the prostate glandular structure. Interestingly, tiny vessels were also visible in all false positive patches. Moreover, we found that false negative patches were associated with having incomplete vessel structure. These observations indicate a high detection sensitivity of our approach for the general vessel appearance and highlight the relevance of the vessel size in influencing the ground truth and consequently the performance. **Table S22** summarizes the confusion matrices for mesenchymal tissues.

Table S22 the detection accuracy for mesenchymal tissues. TN: true negative, FP: false positive, TP: true positive, NPV: negative predictive value.

Test	vPatho			
Finding	Number of patch images (%) – 10x objective magnification			
	FN	TN	FP	TP (Precision)
Blood vessels	513 (16.2)	2656 (83.8)	497 (34.5)	942 (65.5)
Inflammatory cell infiltration	41 (7.9)	477 (92.1)	23 (9.2)	227 (90.8)
Nerve and ganglion	216 (30.2)	499 (69.8)	42 (7.4)	523 (92.6)

Detection of tumor precursors

We examined the detection of the tumor precursors for prostate cancer (High-grade Prostatic Intraepithelial Neoplasia, HGPIN) against benign prostatic hyperplasia (BPH) and intraductal adenocarcinoma (a rare type of prostate cancer). Given that the agreement level for intraductal adenocarcinoma (IDC) was reported to be moderate and usually misinterpreted as HGPIN[5], we considered only lesions that were described in the pathology reports and confirmed by two geographically independent pathologists. We identified that vPatho could distinguish between BPH and HGPIN (Cohen **κ**: 0.856; 95% CI: 0.824 – 0.884; outstanding concordance) as well as between HGPIN and IDC (Cohen **κ**: 0.747; 95% CI: 0.686 – 0.802; substantial concordance), highlighting the potential usage

of AI in differentiating HGPIN from IDC and BPH. Interestingly, vPatho was successful in distinguishing between HGPIN and IDC although the development set did not consider IDC, indicating the classifier robustness of vPatho for HGPIN. **Table S23** is the confusion matrices for the detection of HGPIN and **Table S24** provides the differentiation accuracy.

Table S23 the detection accuracy for High-grade prostatic intraepithelial neoplasia against intraductal adenocarcinoma and benign prostatic hyperplasia. FN: false negative, TN: true negative, FP: false positive, TP: true positive.

Test	High-grade prostatic intraepithelial neoplasia detection			
	Number of patch images (%) – 10x objective magnification (512x512 μm)			
Differential finding (negative finding)	FN	TN	FP	TP
Intraductal adenocarcinoma	41 (25.5)	120 (74.5)	4 (1.9)	209 (98.1)
Benign prostatic hyperplasia	41 (1.7)	2302 (98.3)	11 (5.0)	209 (95.0)

Table S24 the differentiation accuracy for HGPIN vs. IDC (Intraductal adenocarcinoma) and HGPIN (high-grade prostatic intraepithelial neoplasia) vs. BPH (benign prostatic hyperplasia).

	Accuracy for distinguishing HGPIN from differential findings			
Differential finding	Sensitivity, % (95 CI)	Specificity, % (95 CI)	Positive likelihood ratio (95 CI %)	Negative likelihood ratio (95 CI %)
IDC	83.6 (78.4 – 88.0)	96.8 (91.9 – 99.1)	25.92 (9.87 – 68.07)	0.17 (0.13 – 0.22)
BPH	83.6 (78.4 – 88.0)	100 (99.0 – 100)	175.79 (97.24 – 317.78)	0.16 (0.12 – 0.22)

Given that the exact prevalence of HGPIN in prostatic tissues with a dimension of 512 ×512 μm is unknown in the population, the positive (PPV) and negative predictive values (NPV) for HGPIN were estimated at patch level while considering different patch proportions for HGPIN (**Figure S15**). Distinguishing HGPIN from IDC required to have 16% patches showing HGPIN to achieve PPV of 83.3% (95% CI: 64.8 – 94.9%) or NPV of 96.8% (95% CI: 95.7 – 97.7%). Moreover, HGPIN could be accurately differentiated from BPH (PPV: 89.0%; 95% CI: 79.4 – 93.9) when 4% of patches included HGPIN.

Figure S16 illustrates the similarities between these findings and their differences as well. The activation maps for these findings are also illustrated in **Figure S17**.

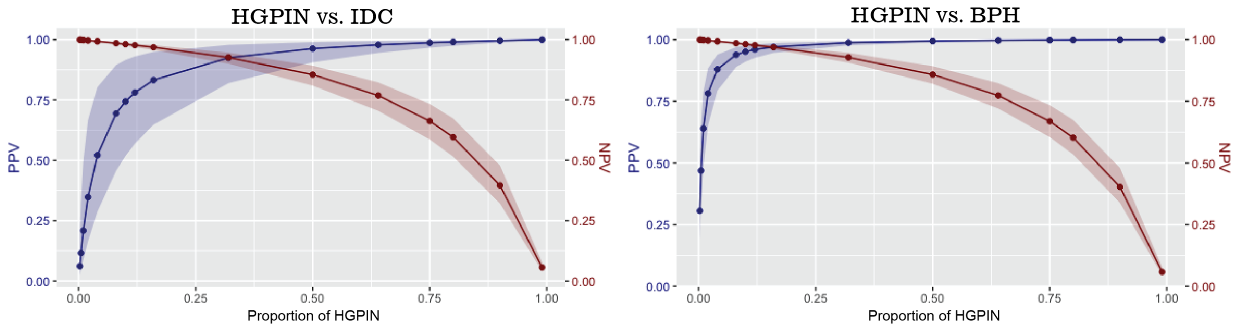

*Figure S15: Estimated positive (PPV) and negative predictive values (NPV) with 95% confidence intervals for different patch proportions of HGPIN in relation to intraductal prostate cancer (IDC) or benign prostatic hyperplasia (BPH) on 10,000 bootstrap resampling of patches. A single patch represents a prostatic tissue with a dimension of 512×512 µm at 10x objective magnification. IDC and BPH are differential findings of HGPIN (See **Figure S16**).*

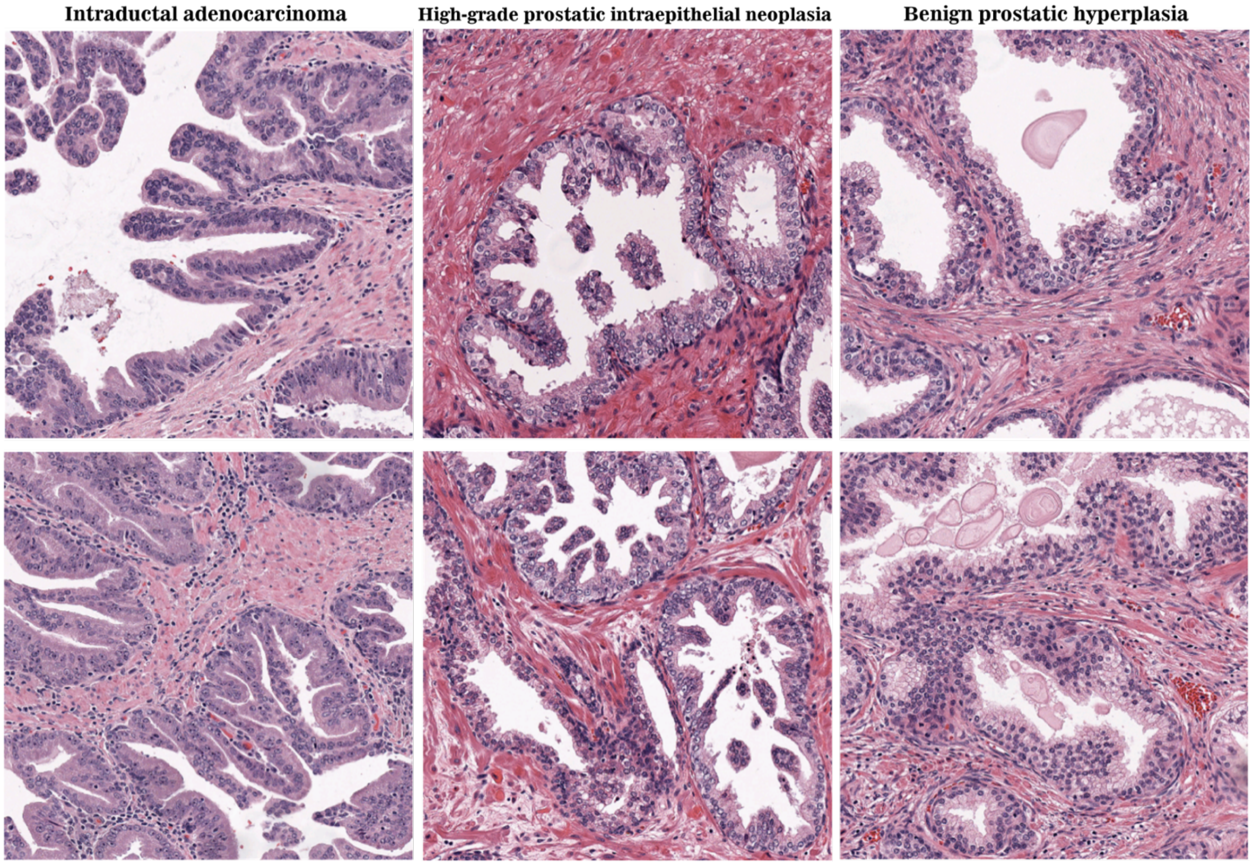

Figure S16 illustrates the fine-grained differences between intraductal adenocarcinoma, HGPIN (high-grade prostatic neoplasia), and benign prostate hyperplasia (BPH). All images were captured at 10x objective magnification level (Width = Height = ~512 µm).

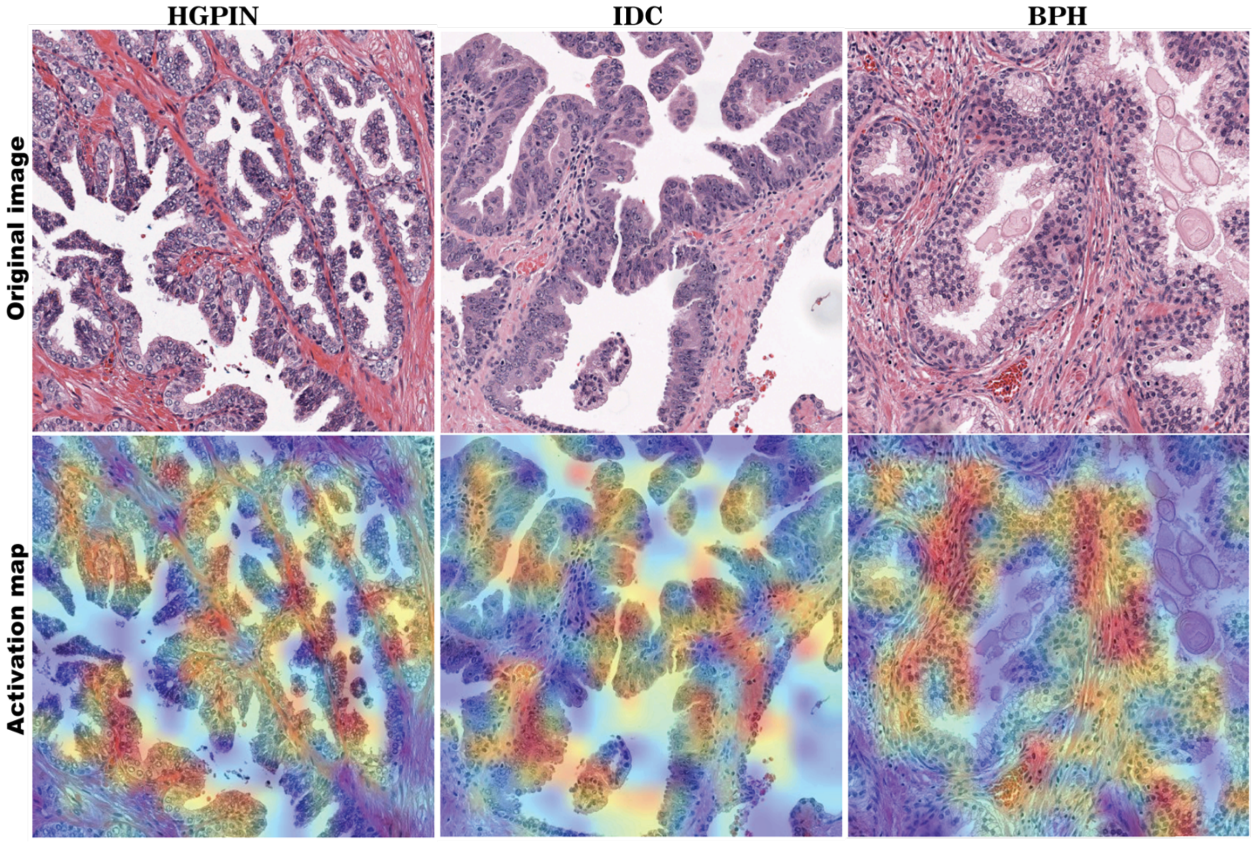

Figure S17 shows the activation maps for HGPIN (High-grade prostatic intraepithelial neoplasia), IDC (intraductal adenocarcinoma) and BPH (benign prostatic hyperplasia). The activation maps reveal which components of the images contributed to the vPatho decisions. All images were captured at 10x objective magnification level (Width = Height = 512 µm). All these images were correctly classified by vPatho (HGPIN vs. others).

Integrating vPatho evaluation results into an electronic Pathology Report

How integrable are the results provided by deep learning into the pathology report for prostatectomy specimens?

We conceptualized a graphical user interface (GUI) that includes an overview graphical illustration of cancer lesions with the corresponding findings. The intuition was that the GUI provides a better summary of the vPatho evaluation in alignment with a previous study revealed that an abstract pathology report with a graphical presentation of tumor extent was associated with positive user experience and a better data quality

and information exchange [1,6]. Moreover, the pathology report was directly reusable for quality control and research.

We proposed a decision-tree algorithm to process patches of histology images of a single case as illustrated in **Figure S18**. This decision-tree algorithm considers a real clinical situation where the Gleason pattern determination is limited due to the presence of nerve structures or inflammatory cell infiltration[7]. The decision thresholds are the same thresholds used in the previous test conditions. Using the decision tree algorithm has reduced the false negative rate for Gleason pattern 4 having cribriform patterns from 34.2% to 26% (8.2% error reduction) at the patch level. Furthermore, the false positive rate was reduced by 10% for Gleason pattern 4 having cribriform patterns.

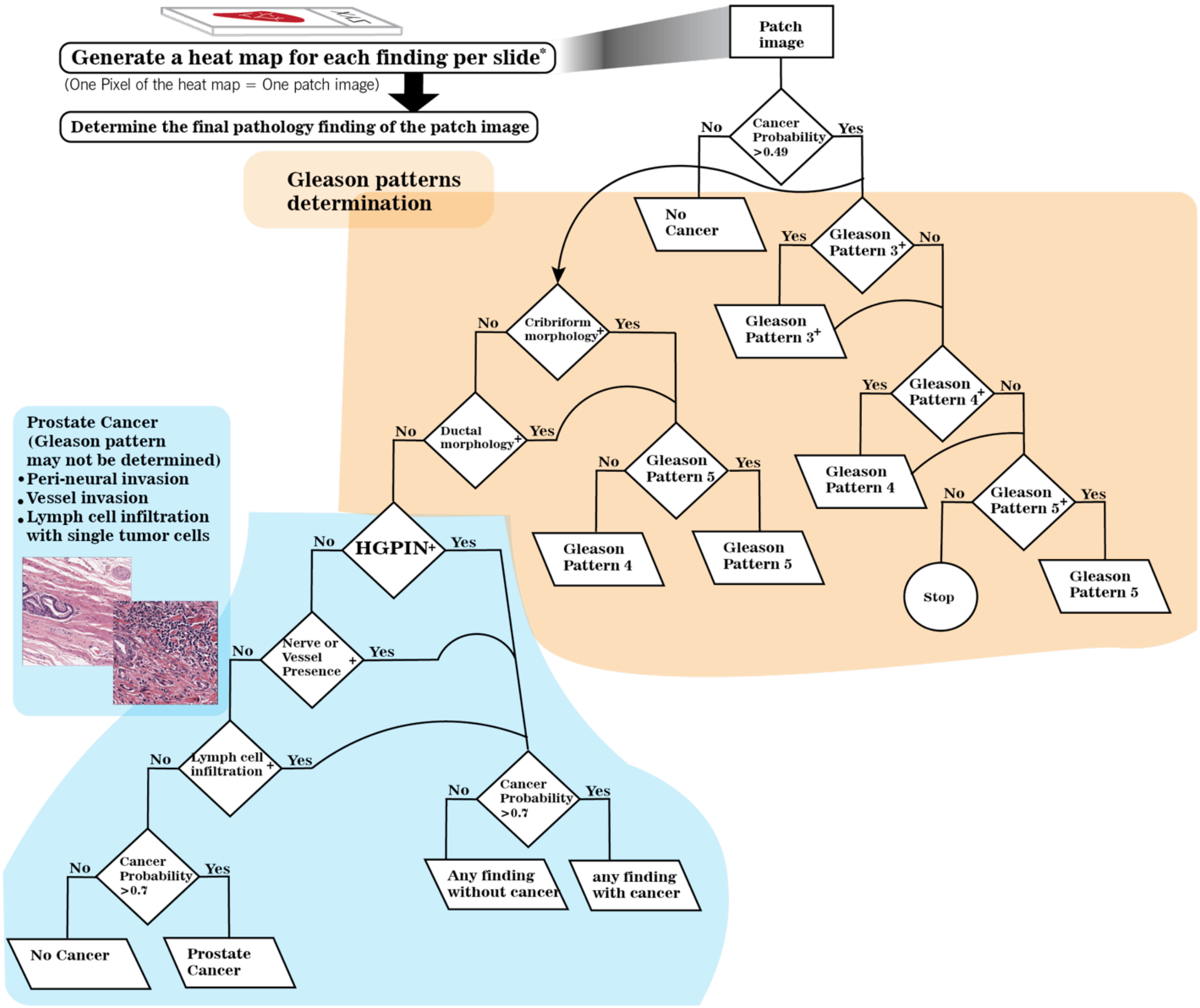

Figure S18 illustrates the decision tree algorithm that we implemented to process patch images that also considers a clinical scenario preventing the Gleason pattern determination at the patch level due to the presence of nerve structure or inflammatory cell infiltration. + the thresholds already described in the supplementary "material and method" section.

We developed a pipeline that combines all components of vPatho and processes the whole-mount images of each case and stores the results into an electronic pathology report inspired from our previous study on the electronic documentation and the graphical report for radical prostatectomy specimens[1]. Here, we utilized the paperless cMDX (Clinical Map Document based on XML) reporting concept for radical

prostatectomy specimens that has been validated for reporting pathology reports in clinical routine[6] and is in use over a period of at least 10 years in a high-volume prostate cancer center. The cMDX documentation system facilitates the extraction of clinical and pathological data collected in the clinical routine, with respect for privacy regulations[6]. Furthermore, cMDX documentation architecture supports the topographical analyses and finding search that we applied to determine the capsule proximity of prostate cancer[8]. Instead of the schematic diagram, we implemented a slide overview with demarcation of the prostate cancer lesions that can be clicked by the clinicians or pathologists to review the representative histology images at 2–5x objective magnification of the corresponding lesions. Furthermore, findings identified in each lesion can be seen on the graphical user interface (GUI). Finally, the access to the gigapixel histology images and the corresponding annotation information generated by vPatho is feasible through GUI.

Our electronic cMDX reporting system provided significant file compression; 136 cMDX reports occupied 163 gigabytes, whereas the corresponding gigapixel histology images required 3.5 terabytes of storage space (95.3% data reduction). The system also provides fast processing speeds using a single GPU and multithread CPU processing (32 threads). Complete image annotation for 136 cases with 8 slides per case on average was processed, and each case required 34±8 minutes, including the time cost for input/output access that can degrade the processing speed. **Figure S19** shows the annotation results.

What information are integrated into the electronic pathology reports?

The user interface provides information on the presence of PC, the annotation of Gleason patterns 3, 4, and 5, the presence of cribriform or ductal morphology, and the tumor volume. **Figure S20** provides an overview of the user interface for viewing the

cMDX reports with integrated results from our approaches for Gleason patterns, ISUP grading and the lesion annotation. As with the original cMDX report editor, the pathologist can manually enter the tumor grade according to the Gleason grading system [7,9], the tumor stage using the Union for International Cancer Control (UICC) tumor-node-metastasis (TNM) staging system [10], and the surgical margin status. **Two example cMDX reports** and **the viewer tool are provided on GitHub (https://github.com/oeminaga/cmdx_report.git)**.

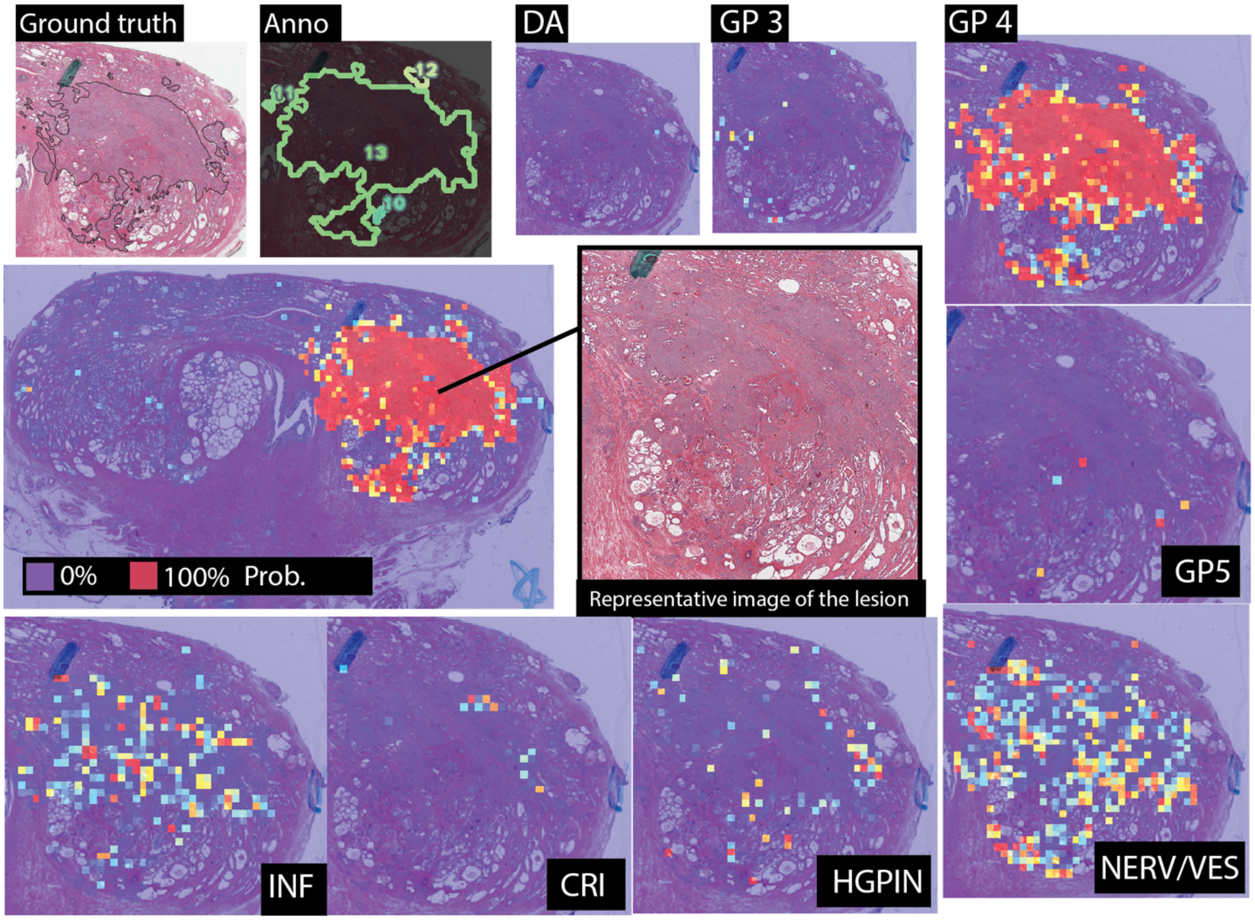

Figure S19 *(A): The ground truth and the corresponding annotations provided by vPatho for prostate cancer and other findings integrated into the electronic pathology report. The generation of the heatmap was repeated for other histological annotations, and the heatmap for prostate cancer was used to automate the determination of the lesion boundary (Anno). PCA: prostate cancer; DA: ductal morphology; CRI: cribriform morphology; Gleason pattern 3 (GP3), 4 (GP4), or 5. Signatures of high-grade prostatic intraepithelial neoplasia (HGPIN); automated segmentation of the tumor lesion (Anno). An example cMDX report for a case is available on GitHub. (B) Activation map for different histological findings and signatures of HGPIN. The activation map is useful to explain the components of the image that led to the assignment of the histological label. A high-resolution for the ground truth and the heatmap for the prostate cancer is provided in* ***Supplemental file 3****.*

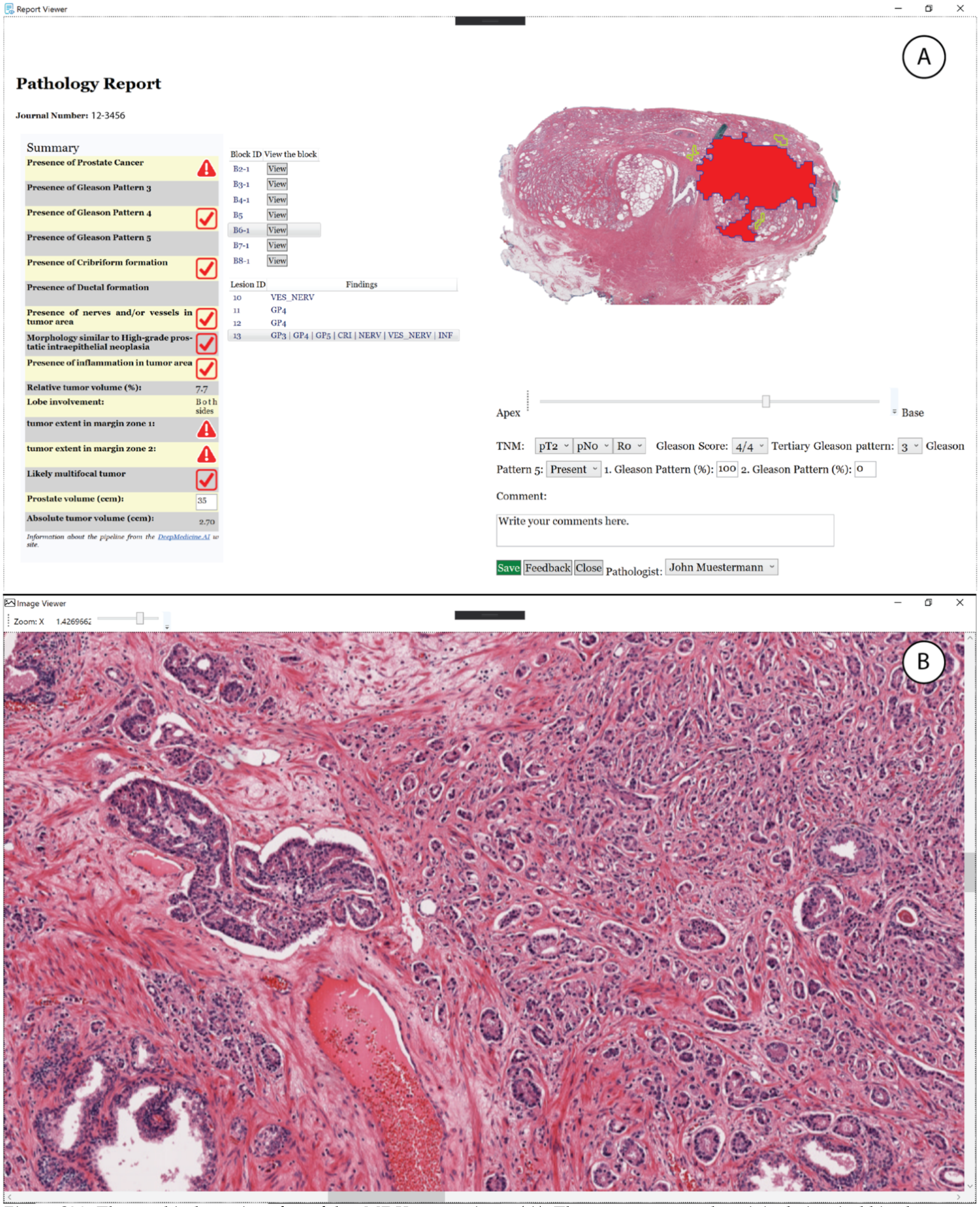

Figure S20: The graphical user interface of the cMDX report viewer (A). The user can access the original gigapixel histology images if these images are available on local storage. By clicking on the lesion (marked with red), a second window showing the region appears (B). The user can zoom in or out and scroll through the lesion. The original input data were altered to protect patient privacy. The cMDX report viewer and two example cases can be obtained from [*https://github.com/oeminaga/cmdx_report.git*](https://github.com/oeminaga/cmdx_report.git)

Activation maps

Figure S21 provides the attention maps for Gleason pattern, ductal morphology, cribriform and mesenchymal tissues considered by this study. These activation maps highlight the components that most likely contribute to the vPatho decision.

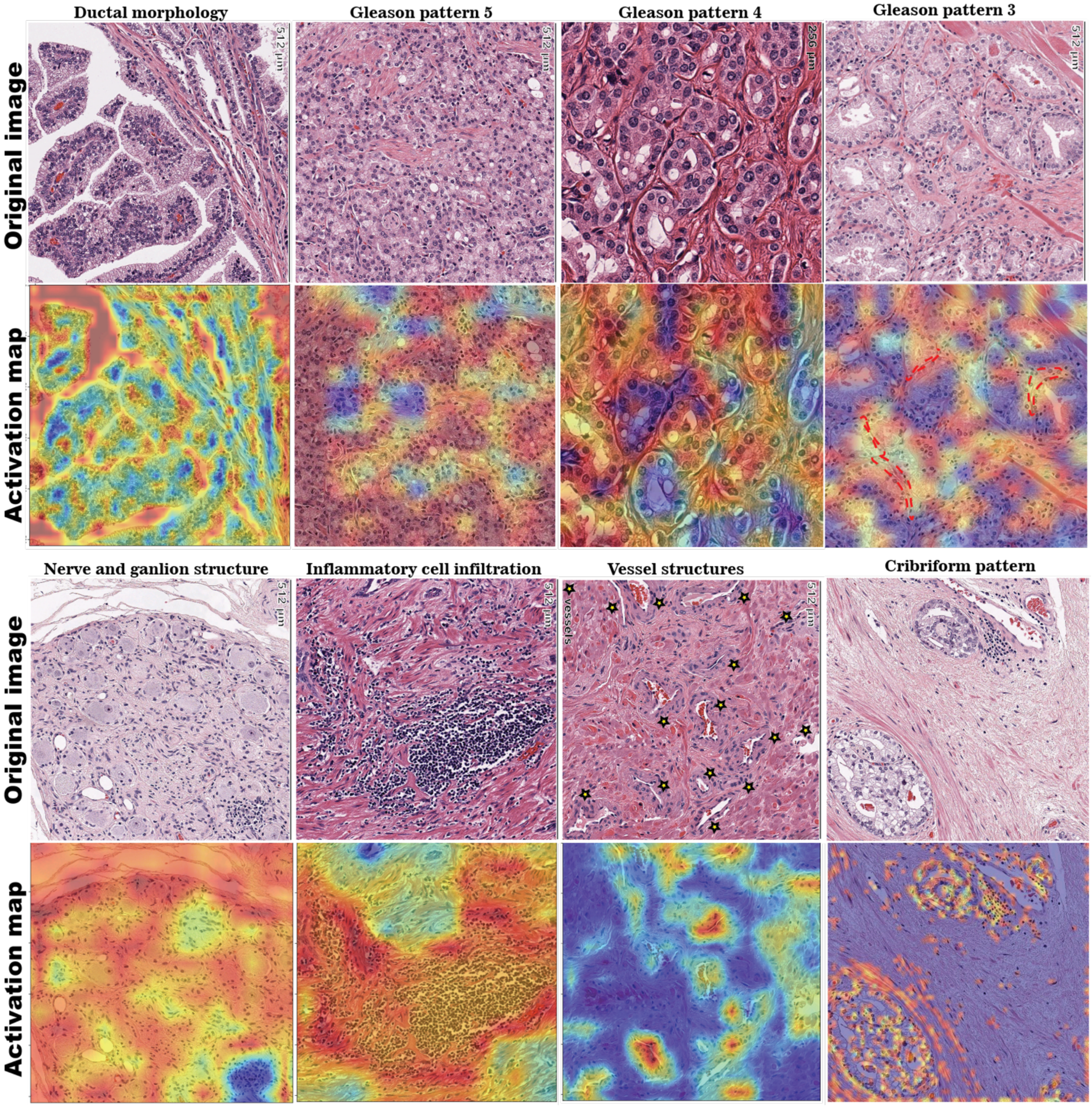

Figure S21: Activation maps for different findings considered by vPatho. Stars highlight the blood vessel.

Addendum table

Table S25: Detection performance of slides with prostate cancer per case (prostatectomy specimen).

Case	No of slides, n	True positive, n	False positive, n	True negative, n	False negative, n	Error rate, %
1	5	5	0	0	0	0
2	7	7	0	0	0	0
3	11	11	0	0	0	0
4	9	9	0	0	0	0
5	4	4	0	0	0	0
6	10	10	0	0	0	0
7	12	12	0	0	0	0
8	8	8	0	0	0	0
9	9	9	0	0	0	0
10	8	8	0	0	0	0
11	6	6	0	0	0	0
12	7	7	0	0	0	0
13	5	5	0	0	0	0
14	7	5	2	0	0	29
15	9	9	0	0	0	0
16	8	7	0	0	1	13
17	10	10	0	0	0	0
18	8	8	0	0	0	0
19	6	6	0	0	0	0
20	3	3	0	0	0	0
21	9	7	1	1	0	11
22	8	8	0	0	0	0
23	7	6	0	1	0	0
24	6	6	0	0	0	0
25	8	8	0	0	0	0
26	8	8	0	0	0	0
27	4	4	0	0	0	0
28	3	3	0	0	0	0
29	9	9	0	0	0	0
30	9	9	0	0	0	0
31	5	4	0	1	0	0
32	9	7	0	2	0	0
33	6	5	0	1	0	0

**34**	7	7	0	0	0	0
**35**	9	8	0	1	0	0
**36**	10	7	0	3	0	0
**37**	6	6	0	0	0	0
**38**	8	8	0	0	0	0
**39**	6	5	0	1	0	0
**40**	8	6	0	2	0	0
**41**	10	10	0	0	0	0
**42**	8	6	0	2	0	0
**43**	9	8	0	1	0	0
**44**	6	6	0	0	0	0
**45**	6	6	0	0	0	0
**46**	4	3	0	1	0	0
**47**	6	6	0	0	0	0
**48**	9	9	0	0	0	0
**49**	8	8	0	0	0	0
**50**	7	7	0	0	0	0
**51**	8	7	0	1	0	0
**52**	9	9	0	0	0	0
**53**	8	7	0	1	0	0
**54**	9	5	0	4	0	0
**55**	5	5	0	0	0	0
**56**	5	5	0	0	0	0
**57**	7	7	0	0	0	0
**58**	6	4	1	1	0	17
**59**	6	6	0	0	0	0
**60**	7	5	0	2	0	0
**61**	4	4	0	0	0	0
**62**	8	8	0	0	0	0
**63**	7	5	0	2	0	0
**64**	8	8	0	0	0	0
**65**	10	10	0	0	0	0
**66**	8	7	1	0	0	13
**67**	9	8	0	1	0	0
**68**	7	7	0	0	0	0
**69**	6	3	0	3	0	0
**70**	9	7	1	1	0	11
**71**	8	7	0	1	0	0
**72**	9	9	0	0	0	0

**73**	6	6	0	0	0	0
**74**	7	6	0	1	0	0
**75**	9	8	0	1	0	0
**76**	3	3	0	0	0	0
**77**	8	6	0	2	0	0
**78**	8	8	0	0	0	0
**79**	11	11	0	0	0	0
**80**	7	6	0	1	0	0
**81**	11	11	0	0	0	0
**82**	6	6	0	0	0	0
**83**	9	8	1	0	0	11
**84**	9	9	0	0	0	0
**85**	8	8	0	0	0	0
**86**	7	7	0	0	0	0
**87**	8	7	0	1	0	0
**88**	10	10	0	0	0	0
**89**	7	7	0	0	0	0
**90**	8	8	0	0	0	0
**91**	6	6	0	0	0	0
**92**	8	8	0	0	0	0
**93**	11	7	0	4	0	0
**94**	9	8	0	1	0	0
**95**	14	11	0	3	0	0
**96**	8	7	0	1	0	0
**97**	7	5	0	2	0	0
**98**	6	6	0	0	0	0
**99**	9	7	0	2	0	0
**100**	8	8	0	0	0	0
**101**	7	7	0	0	0	0
**102**	10	7	0	3	0	0
**103**	7	7	0	0	0	0
**104**	7	7	0	0	0	0
**105**	9	9	0	0	0	0
**106**	6	6	0	0	0	0
**107**	9	5	0	4	0	0
**108**	9	8	0	1	0	0
**109**	14	12	0	2	0	0
**110**	5	3	0	2	0	0
**111**	8	7	0	1	0	0

**112**	9	8	0	1	0	0
**113**	9	9	0	0	0	0
**114**	5	5	0	0	0	0
**115**	7	6	0	1	0	0
**116**	10	10	0	0	0	0
**117**	3	3	0	0	0	0
**118**	9	6	1	2	0	11
**119**	8	7	0	1	0	0
**120**	10	10	0	0	0	0
**121**	3	3	0	0	0	0
**122**	5	4	0	1	0	0
**123**	8	8	0	0	0	0
**124**	7	6	0	1	0	0
**125**	7	7	0	0	0	0
**126**	6	6	0	0	0	0
**127**	5	5	0	0	0	0
**128**	8	7	0	1	0	0
**129**	6	5	0	1	0	0
**130**	3	3	0	0	0	0
**131**	8	8	0	0	0	0
**132**	9	8	0	1	0	0
**133**	7	4	0	3	0	0
**134**	5	5	0	0	0	0
**135**	11	11	0	0	0	0
**136**	8	5	1	2	0	13

References

1. Eminaga O, Hinkelammert R, Semjonow A, et al. Clinical map document based on XML (cMDX): document architecture with mapping feature for reporting and analysing prostate cancer in radical prostatectomy specimens. *BMC Med Inform Decis Mak.* 2010;10:71.
2. Tom MC, Nguyen JK, Luciano R, et al. Impact of Cribriform Pattern and Intraductal Carcinoma on Gleason 7 Prostate Cancer Treated with External Beam Radiotherapy. *J Urol.* 2019;202(4):710-716.
3. Seipel AH, Delahunt B, Samaratunga H, Egevad L. Ductal adenocarcinoma of the prostate: histogenesis, biology and clinicopathological features. *Pathology.* 2016;48(5):398-405.
4. Bock BJ, Bostwick DG. Does prostatic ductal adenocarcinoma exist? *Am J Surg Pathol.* 1999;23(7):781-785.
5. Montironi R, Scarpelli M, Cheng L, Lopez-Beltran A, Zhou M, Montorsi F. Do Not Misinterpret Intraductal Carcinoma of the Prostate as High-grade Prostatic Intraepithelial Neoplasia! *European Urology.* 2012;62(3):518-522.
6. Eminaga O, Abbas M, Hinkelammert R, et al. CMDX(c)-based single source information system for simplified quality management and clinical research in prostate cancer. *BMC Med Inform Decis Mak.* 2012;12:141.
7. Epstein JI, Egevad L, Amin MB, et al. The 2014 International Society of Urological Pathology (ISUP) Consensus Conference on Gleason Grading of Prostatic Carcinoma: Definition of Grading Patterns and Proposal for a New Grading System. *Am J Surg Pathol.* 2016;40(2):244-252.
8. Eminaga O, Semjonow A, Eltze E, et al. Analysis of topographical distribution of prostate cancer and related pathological findings in prostatectomy specimens using cMDX document architecture. *J Biomed Inform.* 2016;59:240-247.
9. Gleason DF, Mellinger GT. Prediction of prognosis for prostatic adenocarcinoma by combined histological grading and clinical staging. *J Urol.* 1974;111(1):58-64.
10. Brierley JD, Gospodarowicz MK, Wittekind C. *TNM Classification of Malignant Tumours.* Wiley; 2016.
